# Supplementary material for: Multicentre phase III randomised controlled trial to evaluate effectiveness of the Both EARS (BEARS) virtual reality training package to maximise hearing abilities in children and young people with bilateral cochlear implants: the BEARS protocol
Source: BMJ Open. 2026 Oct 27;16(10):e128310. doi: 10.1136/bmjopen-2026-128310 (PMC13723878; doi:10.1136/bmjopen-2026-128310)
Supplement: Supplementary file 2 [file bmjopen-16-10-s002.pdf]

Docusign Envelope ID: 5CA44ABD-F65C-8DE8-80E3-4572550DF9E0

BEARS

BEARS
Both Ears

Both **EARS** training package (BEARS) to maximise hearing abilities in older children and teenagers with bilateral cochlear implants

Version	V7.0
Date	14th July 2026
Sponsor	Guy's and St Thomas' NHS Foundation Trust
Comprehensive Clinical Trials Unit Trial Adoption Group #	CTU/2019/330
Trial registration	NCT05808543
REC #	23/YH/0046

The Chief Scientific Investigator and Chief Investigator and the R&D (sponsor office) have discussed this protocol. The investigators agree to perform the investigations and to abide by this protocol. Roles and responsibilities of all investigators have all been outlined in a separately approved document.

The investigator agrees to conduct the trial in compliance with the approved protocol, EU GCP, the UK Data Protection Act (2018), the Trust Information Governance Policy (or other local equivalent), the Research Governance Framework (2005' 2nd Edition; as amended), the Sponsor's SOPs, and other regulatory requirements as amended.

Name	Dr Deborah Vickers
Role	Chief Scientific Investigator
Signature	DocuSigned by: 3F1655C1B25F485...
Date	27-Jul-2026

Name	Professor Dan Jiang
Role	Chief Investigator
Signature	Signed by: 6FF49BD2EB784A0...
Date	27-Jul-2026

Authorisation: Senior Operations Staff

Name	Mr James Blackstone
Role	Head of Clinical Operations
Signature	DocuSigned by: 4BCBCE549E8E46B...
Date	27-Jul-2026

Authorisation: Senior Statistician

Name	Prof Nick Freemantle
Role	Senior Statistician
Signature	DocuSigned by: 19AD28D4AC4F48B...
Date	27-Jul-2026

Docusign Envelope ID: 5CA44ABD-F65C-8DE8-80E3-4572550DF9E0

Table of Contents

Table of Contents 3

General information 11

Sponsor 11

Funding 11

Trial Registration 11

Trial Administration 11

Coordinating Centre 11

Structured trial summary 13

Roles and responsibilities 17

Protocol contributors 17

Role of trial sponsor and funders 17

Trial Team 17

Trial Management Group 18

Programme Steering Committee 19

Data Monitoring and Ethics Committee 19

Trial Diagram 20

Abbreviations 21

Glossary 22

1 Background 25

1.1 Rationale 25

1.2 The BEARS project 26

1.3 The programme's proposed logic model to patient benefit 26

1.4 Games optimisation 27

1.5 Development of outcome measures 27

1.6 The clinical trial 28

1.7 Explanation for choice of comparators 28

1.8 Objectives 28

1.9 Trial Design 29

1.10 Risk Benefit Assessment 29

2 Selection of Sites/Investigators 30

2.1 Site Selection 30

2.1.1 Study Setting 30

2.1.2 Site/Investigator Eligibility Criteria 30

2.1.2.1 Principal Investigator's (PI) Qualifications and Agreements ... 30
2.1.2.2 Resourcing at site ... 30
2.2 Site approval and activation ... 31
3 Selection of Participants ... 32
3.1 Participant Inclusion Criteria ... 32
3.1.2 Guidance on categorising a severe to profound sensorineural hearing loss ... 32
3.2 Participant Exclusion Criteria ... 32
3.2.1 Person of Childbearing Potential ... 33
3.3 Recruitment ... 33
3.4 Re-enrolment and Co-enrolment Guidance ... 34
3.5 Screening Procedures and Informed Consent/Assent ... 35
3.5.1 E-Consent procedure ... 37
3.6 Randomisation of Participants ... 37
4 Trial Intervention ... 39
4.1 Introduction ... 39
4.2 Arm A ... 39
4.2.1 Both EARS training package (BEARS) and Usual Care ... 39
4.2.2 Regulatory Status ... 40
4.2.3 Management (Dispensing & Storage) of intervention equipment ... 41
4.2.4 Interruptions and Discontinuations of the Trial Intervention ... 41
4.3 Arm B ... 42
4.3.1 Usual Care ... 42
4.3.2 Interruptions and Discontinuations ... 42
4.4 Concomitant Care ... 43
4.5 Blinding Considerations ... 43
4.5.1 Other Considerations ... 43
4.5.1.1 Qualitative sub-study ... 43
4.6 Protocol Intervention Discontinuation ... 43
4.7 Compliance and Adherence ... 44
4.7.1 Withdrawal of Consent/assent ... 44
4.8 Eligibility Criteria for Individuals Performing the Outcome Assessments ... 44
5 Assessments & Follow-Up ... 45
5.0 Introduction ... 45
5.1 Outcomes ... 45

5.1.1 Primary Outcome ... 45
5.1.2 Secondary Outcomes ... 45
5.1.2.1 SSiN-VA test outcomes ... 45
5.1.2.2 Spatial ASL test outcomes ... 45
5.1.2.3 British Picture Vocabulary Scale (BPVS) test outcome ... 45
5.1.2.4 VFS-C questionnaire outcome ... 46
5.1.4 Exploratory Analyses ... 46
5.1.4.1 Age effects ... 46
5.1.4.2 Retention of training effects ... 47
5.1.4.3 Impact of degree of balance between ears ... 47
5.2 Participant Pathway ... 48
5.3 Participants randomised from 1st November 2025 ... 50
5.3.1 Participant pathway for those randomised from November 2025 ... 51
5.4 Participant Transfers ... 51
5.5 Early Stopping of Follow-up ... 52
5.6 Loss to Follow-up ... 52
5.7 Post-trial Care ... 52
6 Safety reporting ... 53
6.1 Definitions ... 53
6.1.1 Adverse Events ... 53
6.2 Notifiable Adverse Events (NAEs) ... 54
6.2.1 Pregnancy ... 54
6.3 Investigator responsibilities ... 55
6.3.1 Investigator Assessment ... 55
6.3.1.1 Seriousness ... 55
6.3.1.2 Severity or Grading of Adverse Events ... 55
6.3.1.3 Causality ... 55
6.3.1.4 Expectedness ... 56
6.3.2 Notifications ... 56
6.3.2.1 Notifications by the Investigator to CCTU ... 56
6.2.3.1.1 SAEs ... 57
6.3.2.1.2 NAEs ... 57
6.3.2.1.3 Other AEs ... 57
6.3.2.2 Approach to be Used for Notifications by the Investigator to CCTU ... 57

6.3.2.3 Notifications of CI device issues to CCTU 58

6.4 CCTU responsibilities 58

6.4.1 Urgent Safety Measures 58

7 Quality Assurance & Control 59

7.1 Risk Assessment 59

7.2 Central Monitoring at CCTU 59

7.3 Monitoring 59

7.3.1 Direct access to Participant Records 59

7.3.2 Confidentiality 59

7.4 Source Data 60

7.5 Data Collection and Transfer Methods 61

7.6 Data Management 61

7.7 Data Storage 62

7.8 Data Archiving 62

7.9 Quality Issues 62

8. Statistical Considerations 64

8.1 Sample Size 64

8.2 Assignment of Intervention 64

8.2.1 Randomisation 64

8.2.2 Sequence generation 64

8.2.4 Allocation Implementation 65

8.2.5 Blinding 65

8.3 Statistical Methods 65

8.3.1 Statistical Analysis Plan 65

8.3.2 Interim Analyses 65

8.3.3 Statistical Methods – Outcomes 65

8.3.4 Additional Analyses - Subgroup 66

8.3.5 Additional Analyses - Adjusted 66

8.3.6 Additional Analyses - QoL instrument measurement properties 66

8.3.7 Analysis Population and Missing Data 66

9 Economic Evaluations 67

9.1 Health Economic Analysis Plan 67

9.2 Within-trial analysis 67

9.2.1 Outcomes 67

9.2.1.1 Resource Use ... 67
9.2.1.2 Health related Quality of life ... 67
9.2.3 Cost Data ... 68
9.2.3.1 Cost of the BEARS intervention ... 68
9.2.3.2 Cost of Healthcare resource use ... 68
9.2.4 QALYs ... 68
9.2.5 Analysis results ... 68
9.2.5.1 Primary analysis ... 68
9.2.5.2 Missing data ... 68
9.2.5.3 Sensitivity analysis ... 68
9.2.5.4 Secondary analyses ... 68
9.2.6 Additional analyses ... 68
10 Regulatory & Ethical Issues ... 69
10.1 Compliance ... 69
10.1.1 Regulatory Compliance ... 69
10.1.2 Site Compliance ... 69
10.1.3 Data Collection & Retention ... 69
10.2 Ethical Approvals ... 69
10.2.1 Ethical Considerations ... 69
10.2.2 Ethics Committee Approval ... 70
10.3 Competent Authority Approvals ... 70
10.4 Other Approvals ... 70
10.5 Trial Closure ... 71
11 Indemnity ... 72
12 Finance ... 73
13 Oversight & Trial Committees ... 74
13.1 Trial Management Group ... 74
13.2 Programme Steering Committee ... 74
13.3 Data Monitoring and Ethics Committee ... 74
13.4 Trial Sponsor ... 74
14 Patient & Public Involvement ... 75
14.1 Potential Impact of PPI ... 75
14.2 Identifying PPI Contributors ... 75
15 Publication & Dissemination of Results ... 76

15.1 Publication Policy ... 76
15.1.1 Trial Results ... 76
15.1.2 Authorship ... 76
15.1.3 Reproducible Research ... 76
16 Data Sharing ... 77
16.1 Post Project ... 77
17 Sub Studies ... 78
17.1 Qualitative Research ... 78
17.1.1 Background/rationale ... 78
17.1.2 Study design ... 78
17.1.3 Aims and objectives ... 78
17.1.4 Research Questions ... 79
17.1.5 Theoretical framework ... 79
17.1.6 Participants and setting ... 79
17.1.7 Sampling: in-depth interviews ... 79
17.1.8 Sample identification and recruitment: in-depth interviews ... 80
17.1.9 Data collection procedures ... 80
17.1.9.1 In-depth interviews ... 80
17.1.9.2 Qualitative online survey ... 81
17.1.10 Data analysis ... 81
17.1.10.1 Interview data ... 81
17.1.10.2 Qualitative survey data ... 82
17.1.10.3 Mixed methods integration of qualitative and quantitative data ... 82
17.1.11 Qualitative data management ... 82
17.1.11. Qualitative interview data ... 82
17.1.11.2 Qualitative text-based survey data ... 82
17.1.12 Ethical considerations ... 83
17.1.13 Patient and Public Involvement ... 83
17.1.14 Reporting and dissemination ... 83
17.2 Process Evaluation research Protocol ... 83
17.2.1 Background ... 83
17.2.2 Aims ... 84
17.2.3 Objectives ... 84
17.2.4 Process Evaluation Outputs ... 84

17.2.5 Study Design 84

17.2.6 Research Aims 84

17.2.7 Theoretical framework 85

17.2.8 Sample identification and recruitment 85

17.2.9 Quantitative Data Collection and Analysis 86

17.2.9.1. Overview of Data Collection 86

17.2.9.2 Data Collection from Clinicians, Caregivers and Participants 86

17.2.9.2.1 Clinicians 86

17.2.9.2.2 Caregivers 87

17.2.9.2.3 Participants 87

17.2.10 Data Collection and Data Transfer 87

17.2.11 Data Analysis Plans 87

17.2.12 Qualitative Data Collection 88

17.2.12.1 Case study sites 88

17.2.12.2 Participant and caregiver semi-structured interviews 88

17.2.12.3 Participant qualitative online survey 88

17.2.12.4 Clinician semi-structured focus groups/interviews 89

17.2.13 PE Qualitative Data Management 89

17.2.14 Qualitative Data Analysis 89

17.2.15 Mixed Methods Integrated Interpretation 89

17.2.16 Reporting and Dissemination 90

17.3 BEARS Include Program Development Grant project 90

17.3.1 Background/rationale 90

17.3.2 Study design 90

17.3.5 How this related to Process Evaluation (17.2) 91

17.3.6 Work plan 91

17.3.6.1 WP1: Establish the demographic characteristics of deaf children with cochlear implants in the UK: a scoping review 93

17.3.6.1.1 Method 93

17.3.6.1.2 Inclusion criteria 93

17.3.6.2 WP2: Establish the sociodemographic characteristics of participants recruited to the BEARS clinical trial, and use QRI-Two methods to identify hidden challenges and optimise recruitment number and diversity 93

17.3.6.2.1 Method 93

17.3.6.3 WP3: Explore barriers and facilitators to families taking part in the BEARS clinical trial and how these might impact scale-up ... ***95***

17.3.6.3.1 Method ... 95

17.3.6.3.2 Theoretical framework ... 95

17.3.6.3.3 Participants and setting ... 95

17.3.6.3.4 Sampling and recruitment ... 96

17.3.6.3.5 Data collection ... 96

17.3.6.3.6 Data analysis ... 96

17.3.6.4 WP4: Amend the BEARS scale-up plan based on new learning ... 96

17.3.7 Future work plans ... 97

17.3.8 Patient and Public Involvement ... 97

17.3.9 Dissemination ... 97

17.3.10 Patient and public involvement and engagement ... 98

17.3.11 Ethics ... 98

18 Protocol Amendments ... 100

19 References ... 102

General information

This document was constructed using the Comprehensive Clinical Trials Unit (CCTU) at UCL Protocol template Version 6. It describes the BEARS trial, sponsored by GSTT and co-ordinated by CCTU.

It provides information about procedures for entering participants into the trial, and provides sufficient detail to enable: an understanding of the background, rationale, objectives, trial population, intervention, methods, statistical analyses, cost-effectiveness analyses, ethical considerations, dissemination plans and administration of the trial; replication of key aspects of trial methods and conduct; and appraisal of the trial's scientific and ethical rigour from the time of ethics approval through to dissemination of the results. The protocol should not be used as an aide-memoire or guide for the treatment of other patients. Every care has been taken in drafting this protocol, but corrections or amendments may be necessary. These will be circulated to registered investigators in the trial. Sites entering participants for the first time should confirm they have the correct version through a member of the trial team at CCTU.

CCTU supports the commitment that its trials adhere to the SPIRIT guidelines. As such, the protocol template is based on an adaptation of the Medical Research Council CTU protocol template and the Standard Protocol Items: Recommendations for Interventional Trials (SPIRIT) 2013 Statement for protocols of clinical trials [1]. The SPIRIT Statement Explanation and Elaboration document [2] can be referred to, or a member of CCTU Protocol Review Committee can be contacted for further detail about specific items.

Sponsor

Guy's and St Thomas' NHS Foundation Trust is the trial sponsor and has delegated responsibility for aspects of the trial management of the BEARS trial to UCL's CCTU. Queries relating to sponsorship of this trial should be addressed to the Guy's and St Thomas' NHS Foundation Trust Research and Development Department based at NIHR Guy's and St Thomas' Biomedical Research Centre, 16th floor, Tower Wing, Guy's Hospital, Great Maze Pond, London, SE1 9RT or via the CCTU Trial Team.

Funding

BEARS is fully funded by an NIHR Programme grant number NIHR201608. It is not expected that any further external funding will be sought.

Trial Registration

This trial has been registered with the

1. ClinicalTrials.gov Clinical Trials Register, where it is identified as NCT05808543
2. ISRCTN Clinical Trials Register, where it is identified as ISRCTN92454702

Trial Administration

Please direct all queries to the Trial Manager at UCL CCTU in the first instance; clinical queries will be passed to the Chief Scientific Investigator and Chief Investigator by the Trial Manager.

Coordinating Centre

Comprehensive Clincial Trials Unit at UCL (UCL CCTU)
Institute of Clinical Trials & Methodology

Docusign Envelope ID: 5CA44ABD-F65C-8DE8-80E3-4572550DF9E0

2nd Floor, 9 High Holborn
London
WC1V 6LJ
UK
cctu.bears@ucl.ac.uk

Docusign Envelope ID: 5CA44ABD-F65C-8DE8-80E3-4572550DF9E0

Structured trial summary

Acronym	BEARS
Short title	**B**oth **EARS** training package (BEARS) to maximise hearing abilities in older children and teenagers with bilateral cochlear implants.
Scientific Title	A phase III, unblinded, multi-centre randomised controlled trial to evaluate whether 3 months of spatial-listening training delivered via the **B**oth **EARS** training package (BEARS) in addition to Usual Care compared to Usual Care alone improves hearing abilities and quality of life and is cost effective in older children and teenagers with bilateral cochlear implants.
CCTU Trial Adoption Group #	CTU/2019/330
Sponsor R&D ID #	319903
REC #	23/YH/0046
IRAS #	319903
Primary Registry and Trial Identifying Number	ClinicalTrials.gov Clinical Trials Register: NCT05808543
Date of Registration in Primary Registry	17th March 2023
Secondary Identifying Numbers	NIHR UKCRN Portfolio
Source of Monetary or Material Support	NIHR Programme Grants for Applied Research NIHR201608
Sponsor	Guy's and St Thomas' NHS Foundation Trust (gstt.randd@nhs.net) with sponsor responsibilities delegated to CCTU, UCL.
Contact for Public Queries	ctu.enquiries@ucl.ac.uk
Contact for Scientific Queries	Professor Deborah Vickers<br>University of Cambridge<br>Department of Clinical Neurosciences<br>Herchel Smith Building for Brain and Mind Sciences<br>Robinson Way<br>CB2 0SZ<br>Dav1000@medschl.cam.ac.uk<br>01223 760683<br><br>Professor Dan Jiang<br>Guy's and St. Thomas' NHS Foundation Trust<br>2nd Floor Lambeth Wing, St. Thomas' Hospital<br>Lambeth Palace Road<br>London SE1 7EH<br>dan.jiang3@nhs.net<br>0207 188 0716<br>0207 188 7188 ext. 55042
Countries of Recruitment	UK: England
Health Condition(s) or Problem(s) Studied	Inadequate binaural hearing in young people with bilateral cochlear implants.
Intervention(s)	**Intervention:** 3 months of spatial-listening training delivered via the Both EARS training package (BEARS) comprised of 1 hour per week minimum (separated into a minimum of 2x

	30-minute sessions). This is in addition to routine rehabilitation.<br>**Control:** Usual Care (routine rehabilitation clinical care pathway as described in section 4.3.1).<br>Participants will be randomised 1:1 to the intervention or control group.
Key Inclusion and Exclusion Criteria	**Inclusion Criteria:**<br>1. Participant is a simultaneous or sequential bilateral cochlear implant user*, who either has:<br>a. Congenital severe/profound bilateral sensorineural hearing loss and have received at least one implant ≤ 36 months of age.<br>b. Progressive or acquired severe/profound bilateral sensorineural hearing loss (no age at implant restrictions for these patients)<br>2. Participant has stable programmes (defined as no longer using progressive programmes to work through).<br>3. Participant has had at least two usual care checks/clinical appointments with stable aided levels (+/- 10 dB across 500Hz-4kHz) and no progressive maps to still work through, if they have had re-implantation of internal implant devices.<br>4. Participant is aged 8-16 years, inclusive.<br>*(a bilateral CI user is defined as a patient who uses both CI processors for a minimum of 6-hours per day over a month)<br><br>**Exclusion Criteria:**<br>1. Participant (or parent/legal representative) does not speak/understand English sufficiently to undertake assessments.<br>2. Participant has an intellectual disability at a level that would prevent their ability to understand the trial the intervention or assessment questions.<br>3. Participant has a comorbid condition impacting ability to participate in intervention and/or outcome assessment.<br>4. Participant has an audiological profile impacting ability to participate in intervention and/or outcome assessments.<br>5. Participant is actively participating in other trials that may affect hearing outcomes or impact their ability to participate in the intervention.<br>6. Participant is currently or anticipated to receive treatment and/or intervention that may affect hearing outcomes or adapt implant settings/programming.<br>7. Participant is refusing to consent to trial activities/protocol.<br>8. Participant is awaiting reimplantation following device failure or infection.<br>9. Participant is a non-user of one or both implant processors (i.e., must use both processors for a minimum of 6 hours per day over a month).

	10. Participant is a fulltime boarder at a boarding school<br>11. Participant has unresolvable issues found in device checks that render one of the implants unusable.<br>12. Participant is pregnant.<br>13. Participant has a diagnosis of epilepsy or history of seizures of any kind.
Study Type	BEARS is a UK multi-centre, unblinded, interventional, randomised controlled, phase III trial
Study setting	Secondary care: hearing implant centres
Planned Date of First Enrolment	28-February-2023
Target Sample Size	272 young people (aged 8-16 inclusive) with bilateral cochlear implants (136 patients per group).
Trial Duration	2 years, 10 months and 1 days
Primary Outcome(s)	The Primary outcome for the BEARS trial is the difference between the intervention groups in speech-in-noise perception score (% correct overall task) at 3 months, accounting for the participant's baseline score. This is derived from the spatial speech in noise (SSiN-VA) test.
Key Secondary Outcomes	SSiN-VA test outcomes:<br>- Speech-in-noise perception score (% correct of the overall task) at twelve months accounting for the baseline score.<br>- Relative localisation score (% correct) at three months and at twelve months accounting for the baseline score.<br>- Average reaction time (measure of listening effort) for word identification selections at three months and at twelve months accounting for the baseline score.<br>- Average reaction time (measure of listening effort) for location shift selection at three months, and at twelve months accounting for the baseline score.<br>- Spatial index for word identification at three months and at twelve months accounting for the baseline score.<br>- Spatial index for relative localisation at three months and at twelve months accounting for the baseline score.<br><br>Spatial ASL test outcomes:<br>- Speech reception threshold at three months and at twelve months, accounting for baseline (for better ear, worse ear, and average of both).<br>- Spatial release from masking score at three months and at twelve months, accounting for baseline (for better ear, worse ear, and average of both).<br><br>British Picture Vocabulary Scale (c) test outcome:<br>- Difference between arms in vocabulary age at twelve months, accounting for baseline vocabulary age.

	Vanderbilt Fatigue Scale: Child self-report version (VFS-C) questionnaire outcome:<br>Difference between arms in listening-related fatigue score at three and 12 months, accounting for baseline.<br><br>Health Economic outcomes:<br>The economic evaluation will calculate incremental cost per quality-adjusted life-year (QALY) gained by offering BEARS and usual care, compared to usual care, from an NHS, Personal Social Services (PSS) and Local Education Provider perspective over the twelve months of the trial.
Exploratory Outcomes	Age effects:<br>No further data will be collected for the age effect analyses but the data regarding the trajectory of the children's speech-in-noise and other secondary outcome scores relative to normative age ranges for the speech measures and other measures will be assessed to determine if there is a difference in trajectory over time between the BEARS intervention and Usual care groups.<br><br>Retention of training effects:<br>No further data will be collected for the retention effect analyses. It is considered best practice in training interventions to evaluate if the training effects remain after the intervention period has finished. For this analysis fixed effects of timepoint (baseline, 3, and 12 months) and group (BEARS intervention, Usual care) and random effect of participant will be used to understand the speech-in-noise outcomes.<br><br>Impact of degree of balance between ears:<br>For this analysis, the balance app will be used to indicate the degree of symmetry across the two ears. The balance measurements at baseline, three months and 12 months will be used as an outcome to determine if there have been any changes in the balance between the ears over time.

Roles and responsibilities

These membership lists are correct at the time of writing; please see terms of reference documentation in the TMF for current lists.

Protocol contributors

Name	Affiliation	Role
Prof Deborah Vickers	University of Cambridge	Chief Scientific Investigator
Prof Dan Jiang	GSTT	Chief Investigator
Dr Caroline Clarke	UCL	Senior Health Economist
Dr Hadassa Merle Mahon	UCL	Qualitative Researcher
Ms Marie Le Novere	UCL	Health Economist
Dr Ruth Nightingale	UCL	Qualitative Researcher
Dr Giorgos Dritsakis	University of Cambridge	Post-doctoral Clinical Research Associate
Dr Bhavisha Parmar	University of Cambridge	Post-doctoral Research Paediatric Audiologist
Professor Padraig Kitterick	University of Nottingham/	Principal Hearing Researcher
Dr Adam Pedley	University of Nottingham	Research Fellow in Hearing Sciences
Dr Sarah Somerset	University of Nottingham	Health Researcher / Research Fellow
Mrs Sandra Driver	GSTT	Clinical Lead Speech and Language Therapist
Dr Fleur Corbett	Imperial College London	Developmental Scientist -Process Evaluation
Dr Nejra Van Zalk	Imperial College London	Developmental Scientist – Process Evaluation
Dr Frances Early	Cambridge University Hospitals NHS Foundation Trust	Qualitative Researcher – Process Evaluation
Ms Liz Arram	UCL CCTU	Clinical Trial Manager
Dr Leanne Crisp	UCL CCTU	Clinical Trial Manager
Mrs Lisa French	UCL CCTU	Clinical Trial Manager
Mr James Blackstone	UCL CCTU	Clinical Project Manager
Prof Nick Freemantle	UCL CCTU	Co-Investigator / Senior Statistician
Ms Kashfia Chowdhury	UCL CCTU	Oversight Statistician
Ms Norin Ahmed	UCL CCTU	Trial Statistician

Role of trial sponsor and funders

Name	Role
Guy's and St Thomas' NHS Foundation Trust	Sponsor
NIHR Programme Grant for Applied Research	Funder

Trial Team

Name	Affiliation	Role and responsibilities
Prof Deborah Vickers	University of Cambridge	Chief Scientific Investigator
Prof Dan Jiang	GSTT	Chief Investigator
Prof Nick Freemantle	UCL CCTU	Co-Investigator / Senior Statistician
Mr James Blackstone	UCL CCTU	Clinical Project Manager
Ms Liz Arram	UCL CCTU	Clinical Trial Manager

Dr Teresa Hill	UCL CCTU	Data Manager
Ms Kashfia Chowdhury	UCL CCTU	Oversight Statistician
Ms Norin Ahmed	UCL CCTU	Trial Statistician
Dr Giorgos Dritsakis	University of Cambridge	Post-doctoral Clinical Research Associate
Dr Bhavisha Parmar	University of Cambridge	Post-doctoral Research Paediatric Audiologist
Dr Marina Salorio-Corbetto	University of Cambridge	Post-doctoral Senior Research Audiologist
Mrs Sandra Driver	GSTT	Clinical Lead Speech and Language Therapist

Trial Management Group

Name	Affiliation	Role and responsibilities
Prof Deborah Vickers	University of Cambridge	Chief Scientific Investigator
Prof Dan Jiang	GSTT	Chief Investigator
Prof Nick Freemantle	CCTU	Co-Investigator / Senior Statistician
Mr James Blackstone	CCTU	Clinical Project Manager
Ms Liz Arram	CCTU	Clinical Trial Manager
Dr Teresa Hill	CCTU	Data Manager
Ms Kashfia Chowdhury	CCTU	Oversight Statistician
Ms Norin Ahmed	CCTU	Trial Statistician
Dr Caroline Clarke	UCL Priment CTU	Senior Health Economist
Dr Ekaterina Bordea	CCTU	Health Economist
Dr Lorenzo Picinali	Imperial College London	BEARS Software Lead
Dr Pádraig Kitterick	University of Nottingham	Principal Hearing Researcher
Dr Adam Pedley	University of Nottingham	Research Fellow in Hearing Sciences
Dr Sarah Somerset	University of Nottingham	Health Researcher / Research Fellow
Dr Giorgos Dritsakis	University of Cambridge	Post-doctoral Clinical Research Associate
Dr Bhavisha Parmar	University of Cambridge	Post-doctoral Research Paediatric Audiologist
Dr Marina Salorio-Corbetto	University of Cambridge	Senior Research Audiologist
Dr Hadassa Merle Mahon	UCL	Qualitative Researcher
Dr Ruth Nightingale	UCL	Qualitative Researcher
Prof Andrew Steptoe	UCL	Senior Behavioural Scientist
Prof Stuart Rosen	UCL	Expert in Speech and Hearing Science
Mrs Sandra Driver	GSTT	Clinical Lead Speech and Language Therapist
Mrs Christine Rocca	GSTT	Teacher of the Deaf
Dr Nejra Van Zalk	Imperial College London	Developmental Scientist – Process Evaluation
Dr Fleur Corbett	Imperial College London	Developmental Scientist -Process Evaluation

Dr Frances Early	Cambridge University Hospitals NHS Foundation Trust	Qualitative Researcher – Process Evaluation
Prof Helen Cullington	University of Southampton	Principal Clinical Scientist
Ms Helen Dean	Parent	PPI Representative
Tara Hilson	Parent	PPI Representative

Programme Steering Committee

Name	Affiliation	Role
Ms Ruth Thomsen	NHS England	Chair
Mr David McNulty	University Hospitals Birmingham	Statistician
Ms Sarah Hughes	University of Birmingham	Qualitative researcher
Dr Debi Vickers	University of Cambridge	Chief Scientific Investigator
Prof Dan Jiang	GSTT	Chief Investigator
Prof Nick Freemantle	UCL CCTU	Lead trial statistician
Tricia Kemp	Parent	PPIE
Other co-applicants can attend where appropriate		

Data Monitoring and Ethics Committee

Name	Affiliation	Role
Laura Turton	NHS Tayside	Chair
Jameel Muzaffar	University Hospitals Birmingham NHS Foundation Trust	Independent Member
Kate Bennett	University of Surrey	Independent Statistician

Docusign Envelope ID: 5CA44ABD-F65C-8DE8-80E3-4572550DF9E0

Trial Diagram

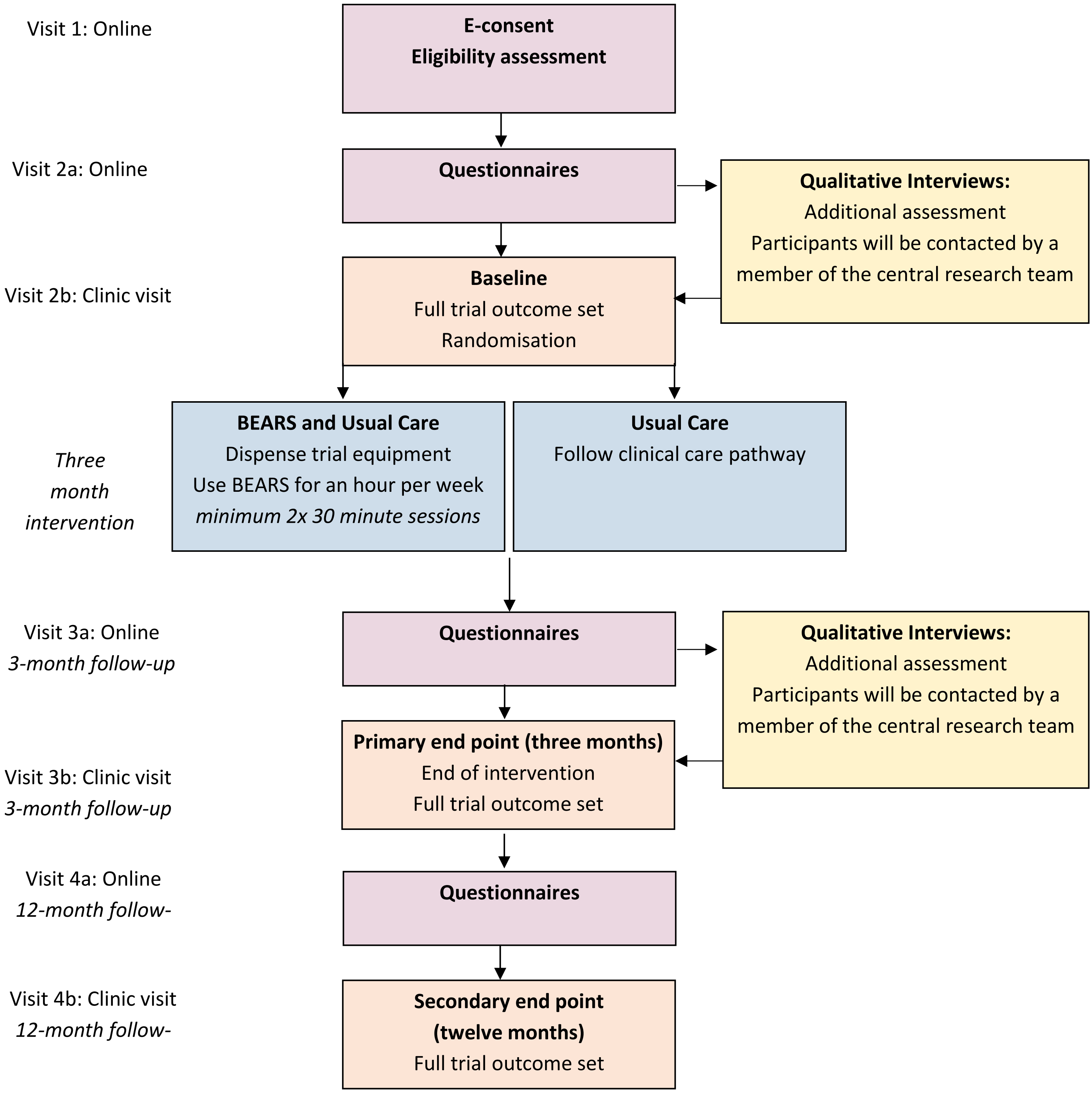

***Please note that as per section 5.3 of the protocol, any participants recruited onto the trial from the 1st November 2025 will end their participation after visit 3b.**

Abbreviations

AE	Adverse Event
AR	Adverse Reaction
BATOD	British Association of Teachers of the Deaf
BEARS	Both EARS training package
BPVS	British Picture Vocabulary Scale
CAP	Categories Auditory Perception
CEA	Cost-Effectiveness Analysis
CEAC	Cost-Effectiveness Acceptability Curve
CEP	Cost-effectiveness Plan
CHU9D	Child Health Utility 9 Dimensions
CCTU	Comprehensive Clinical Trials Unit at UCL
CI	Chief Investigator
CI	Cochlear Implant
CRF	Case Report Form
CSRI	Client Service Receipt Inventory
CTCAE	Common Terminology Criteria for Adverse Events
DHH	Deaf or Hard of Hearing
DMEC	Data Monitoring and Ethics Committee
EC	Ethics Committee
EDC	Electronic Data Capture
GCP	Good Clinical Practice
GP	General Practitioner
GSTT	Guy's and St Thomas' NHS Foundation Trust
HE	Health Economist
HEAP	Health Economics Analysis Plan
HRA	Health Research Authority
HUI-3	Health Utilities Index Mark 3
ICER	Incremental Cost-Effectiveness Ratio
ICH	International Conference on Harmonisation
ICF	Informed Consent Form
IMPD	Investigational Medicinal Product Dossier
IRAS	Integrated Research Application System
ISF	Investigator Site File
ISRCTN	International Standard Randomised Controlled Trial Number
ITT	Intention to Treat
MRC	Medical Research Council

NAE	Notifiable Adverse Event
NHS	National Health Service
NIHR	National Institute for Health and Care Research
NOMAD	Normalisation Measure Development questionnaire
NPT	Normalisation Process Theory
PI	Principal Investigator
PIN	Participant Identification Number
PIS	Participant Information Sheet
PPI	Patient and Public Involvement
PSC	Programme Steering Committee
PSS	Personal Social Services
QA	Quality Assurance
QALY	Quality-Adjusted Life-Years
QC	Quality Control
QMMP	Quality Management and Monitoring Plan
QOL	Quality of Life
R&D	Research and Development
REC	Research Ethics Committee
SAE	Serious Adverse Event
SAP	Statistical Analysis Plan
SAR	Serious Adverse Reaction
SIR	Speech Intelligibility Rating
SOP	Standard Operating Procedure
Sp-ASL	Spatial Adaptive Sentence List
SRM	Spatial Release from Masking
SRT	Speech Reception Threshold
SSiN- VA	Spatial speech in Noise - Virtual Acoustics
TMF	Trial Master File
TMG	Trial Management Group
ToR	Terms of Reference
UCL	University College London
VFS-C	Vanderbilt Fatigue Scale: Child self-report version
YBHRQL-Y	York Binaural Hearing-Related Quality of Life questionnaire for young people/youths

Glossary

Term	Definition
Balance application	The AXP-iBalance application is used as a measure of balance of sound across cochlear implants. The goal of the application is to determine whether the intensity levels of sound at the two ears is the same or whether one ear has higher levels.<br><br>The application works via an iPad, sounds are played to both ears simultaneously and the patient is asked to adjust a dial until the sound seems to come from the front. The intensity levels are recorded of each ear that led to a 'centralised' sound to determine if there is imbalance. This is repeated three times, with the average calculated.<br><br>The test is completed across different frequency regions and using a broadband sound.<br><br>The Balance application is an additional test being completed as part of the BEARS clinical trial. The test will be uploaded onto the iPads given to the sites. Pseudo anonymised data will be collected on the iPads.
British Picture Vocabulary Scale (BPVS)	The BPVS is a vocabulary assessment that can assess a child's receptive (hearing) vocabulary. The assessment is carried out by the assessor saying a word, which the child responds to by selecting a picture from four options that best illustrates that word's meaning [3].
Child Health Utility 9 Dimensions (CHU9D)	The CHU9D is a paediatric generic preference-based measure of health-related quality of life. It consists of a short questionnaire and a set of preference weights using general population values. The questionnaire has nine questions with five response levels per question and is self-completed by the child. The CHU9D allows the analyst to obtain quality adjusted life years (QALYs) directly for use in cost utility analysis [4].
Client Service Receipt Inventory (CSRI)	The CSRI is used to collect information about service utilisation, income, accommodation and other cost-related variables. Its primary purpose is to allow resource use patterns to be described and support costs to be estimated using an appropriate unit cost. Typically, the CSRI has five sections. It can be adapted at the start of each study to ensure it is compatible in format, language, scope and content with the research aims, context, participants' likely circumstances, and the quantity and precision of information required. It is also adaptable in administration. Although originally designed to be administered to the service user or their carer face to face with a trained interviewer, the CSRI has been successfully administered via post for the service user to complete by themselves. It will be used in BEARS to capture health and social care resources use among the teenage target population during the trial [5].
Clinician	A health professional engaged in the care of patients. It includes physicians and allied health care professionals.
Health Utilities Index (HUI-3)	The HUI-3 is a system used to measure health status, report health related quality of life and produce utility scores. The health status classification systems assess eight attributes: vision, hearing, speech, ambulation, dexterity, emotion, cognition and pain. The HUI has demonstrated significant sensitivity to hearing loss [6].

Normalisation Measure Development (NoMAD) questionnaire	The Normalisation Measure Development questionnaire (NoMAD is a measure that can be used to understand implementation participants' experiences. It is intended as a measure that can be used alongside instruments that measure other dimensions of implementation activity, such as implementation fidelity, adoption, and readiness [7]. The questionnaire is a set of 23 survey items.
Relative Localisation Performance	This is a result obtained from the SSiN-VA (detailed below). It is the score for the identification of location shift between the first and second word, presented as part of the SSiN-VA assessment. A % correct score can be obtained from each mean speaker location (location between the first and second word). The overall mean score would be obtained from averaging across all mean speaker localisations.
Spatial Adaptive Sentence List (Sp-ASL)	The Sp-ASL, is an adaptative speech test [8].<br><br>During the test, participants are presented with sentences in noise where the speech level is fixed but the level of noise varies every trial. Listeners are asked to repeat the sentences and are scored on the number of key words they repeat. If the listener identifies 2 or 3 key words correctly, the level of noise in the next trial increases to make the test harder. This adaptive staircase procedure continues until the Speech Reception Threshold (SRT) is found. The SRT is the minimum signal to noise ratio (SNR) that elicits 50% correct speech recognition, a low SRT is a better score. The Sp-ASL will be completed in three conditions:<br>a. When noise and speech is coming from the front<br>b. When noise is at the front and speech is on the right (or better ear if preference)<br>c. When noise is at the front and speech is on the left (or worse ear if preference)<br>There will be an overall speech reception threshold score from each condition, from which an average score can be calculated. By investigating the difference between the conditions, we can calculate the spatial release from masking scores. Two spatial release from masking scores can be calculated: b-a and c-a.
Spatial Release from Masking (SRM)	SRM is the term used to describe the improvement in masked speech recognition, when the target and masker are spatially separated on the horizontal plane compared to when they are co-located [9].<br><br>The SRM will be measured during the Sp-ASL to look at changes in participants performance when separating the speech signal and location of the noise. The distance between the location of the speech and noise per trial can then be analysed against the participants word identification performance.<br><br>A proxy for SRM will be measured during the Spatial Speech in Noise-Virtual Acoustics (SSiN-VA) test an index of performance across different speech/noise separations.
Spatial Speech in Noise - Virtual Acoustics Test (SSiN-VA)	The SSiN-VA is a version of the Spatial speech in noise test that is implemented in Virtual Acoustics. It was developed to simultaneously assess relative localisation and word identification performance [10]. For this test, the participant will complete a listening task. They will hear

	two words in the presence of background noise and be asked to identify both words and the location shift between them (left or right). An overall percentage correct for word identification (speech perception) and relative localisation can be calculated from this.<br>Participants will use a touchscreen to make their selections, with reaction times being recorded for each of the three responses (when identifying the two words and when identifying the location shift). Reaction times can be used as a proxy for measuring listening effort. During the task, the words are presented from different speaker locations, and the background noise also varies by location. Therefore, it is important to explore how the listener's performance varies as a function of the distance between the speech and noise. A spatial index taking these considerations into account will be calculated for relative localisation and word identification performance.
Vanderbilt Fatigue Scale (VFS-C)	The VFS-C is a unidimensional scale designed to assess listening-related fatigue in children ages 6-17 years. This self-reported measure quantifies listening-related fatigue from the child's own perspective. The scale has ten questions, which are answered on a numerical scale from 0-4 [11].
Word Identification Performance	This is a result obtained from the SSiN-VA (detailed above). This results from the participants identifying the first and second words on a touchscreen tablet, presented as part of the SSiN-VA assessment. A % correct score can be obtained from each speaker location. Overall mean score would be obtained from averaging across all locations.
York Binaural Hearing-Related Quality of Life questionnaire for young people/youths (YBHRQL-Y)	The YBHRQL-Y questionnaire has been adapted from the YBHRQL [12]. It is a hearing specific, preference-based-measure of quality-of-life in children. This measure is made of three hearing domains (speech-in-noise, localisation, effort-and-fatigue) each scored on a five-point scale. This measure describes 243 unique health states. Health-utility values are provided that estimate the disease burden of each health state.
Young people	For the purposes of this trial 'young people' is defined as children between the ages 8 and 16 years (inclusive).
Younger children	For the purposes of this trial 'younger children' is defined as children between the ages 8 and 12 years (inclusive).

1 Background

1.1 Rationale

Cochlear Implants (CIs) are medical devices implanted in the ears that send electricity to the hearing nerve, allowing people with significant deafness to hear. There are approximately 10,400 children aged under 20 years with severe-to-profound deafness in the UK [13], with cochlear implantation being the primary intervention. The National Institute for Health and Care Excellence [13, 14] recommends deaf children have bilateral cochlear implantation, to provide the advantages of hearing through two ears rather than one. Currently, 5426 people have bilateral CIs and approximately 475 new children implanted annually in the UK [15].

Speech communication is crucial for cognitive, social and language development and often takes place in complex noisy environments. For normal-hearing listeners, subtle differences in timing and level of sounds reaching each ear provide directional cues [16-19] which contribute to the separation of speech from noise and the ability to attend to a particular speaker among other abilities [20-22]. Children and teenagers are the largest groups of those bilateral implanted and spend substantial time in noisy environments, impacting on their speech and language development, education, and social well-being. Hearing impairment puts older children and teenagers with CIs at a greater risk of emotional and behavioural difficulties that impacts on social relationships with their peers [23, 24].

Although language development [25-27], sound localisation [28-34], tracking of moving sound sources speech-in-noise perception [27, 28, 35] and listening effort [36] are better for people with bilateral CIs than for those with a unilateral implant [37, 38] these skills remain far below those of normally-hearing children. People with bilateral CIs often struggle to understand speech in noise even with visual cues [39]. Our 'Living with Cochlear Implants' Patient and Public Involvement (PPI) group report documents everyday communication as being challenging and tiring due to the extra effort required, especially in noise. Bilateral CI users report how it is hard to balance sounds from two implants and describe sounds from the second implant as 'annoying', 'distracting' and 'lop-sided', leading to its rejection.

The lack of (a) protocols for fitting bilateral CIs, (b) ecologically valid outcome measures, and (c) resources for spatial-hearing training contribute to these listening difficulties. The current rehabilitation assessments and techniques completed with bilateral CI patients are not engaging, do not reflect real-world hearing difficulties experienced by users and do not feature specific training to make the most of having two implants. A large body of research demonstrates that sound localisation can improve with training, underpinned by plasticity-driven changes in the auditory pathways for children and adults [40-57]. Paediatric CI users report that computer-based rehabilitation assessments would better fit their lifestyles and support their learning. By providing engaging training using videogames that are relevant to young people, it is expected that positive brain changes can occur to maximise the use of spatial-hearing skills. Implementing training to improve spatial hearing in older children with bilateral CIs has the potential to improve auditory performance in real-life scenarios, which would have positive repercussions in social, emotional, and academic aspects of life. However, no training programme has been implemented and evaluated for young people with bilateral CIs.

1.2 The BEARS project

To address this gap, through a series of PPI groups we have developed the **B**oth **Ears** training package (BEARS) of virtual-reality games to train spatial hearing in young people with bilateral CIs. The BEARS training package comprises of three games addressing different hearing functions: speech-in-noise perception, music listening and sound-source localisation. Each game is based on an audio-visual task performed through a virtual-reality interface. Players are automatically guided through on-screen visual prompts to support the gameplay with feedback given on their performance. Players progress through levels of increasing difficulty.

The aims of the BEARS Programme grant (NIHR201608), of which this randomised controlled trial forms one part, are:

1. To refine the BEARS training package (ongoing work in preparation for the clinical trial included in a separate protocol).
2. To develop tools to evaluate the effectiveness of the BEARS training package (ongoing work in preparation for the clinical trial included in a separate protocol).
3. To conduct a large-scale confirmatory clinical trial to assess whether BEARS substantially improves hearing with two implants (current protocol).
4. To understand the learning mechanism and process evaluation (independent process, complementary to the trial).

1.3 The programme's proposed logic model to patient benefit

The BEARS programme follows the NIHR/MRC framework on complex interventions to improve health [58]. To understand the mechanism of action, we will work with the premise that the BEARS training package facilitates learning. We will look at behaviour change per se when thinking about uptake and usability and this will be evaluated within the trial and process evaluation. The benefits of the hearing functions addressed in the BEARS games are evidenced within literature [59]. These hearing functions lead to improved listening skills, which in turn improve self-confidence for engaging in communication without the fear of embarrassment. As communication becomes easier, self-confidence continues to develop, improving development in multiple areas of life such as building relationships and education.

We have based our model on developmental theory which considers the biological, psychological, social and emotional changes that occur with age. We would expect our study population to have reached the 'formal operations' stage such that they can develop an understanding of the world. They will also have reached a 'cognitive stage' which is linked to the proposed change mechanisms. As hearing improves, this should develop knowledge construction of the world, increasing self-confidence and socio-emotional development (i.e., improve experience, expression, management of emotions and ability to establish positive relationships with others).

A logic model has been created using the MRC framework on complex interventions to improve health, figure 1:

Docusign Envelope ID: 5CA44ABD-F65C-8DE8-80E3-4572550DF9E0

Figure 1: Logic model created using the MRC framework on complex interventions to improve health.

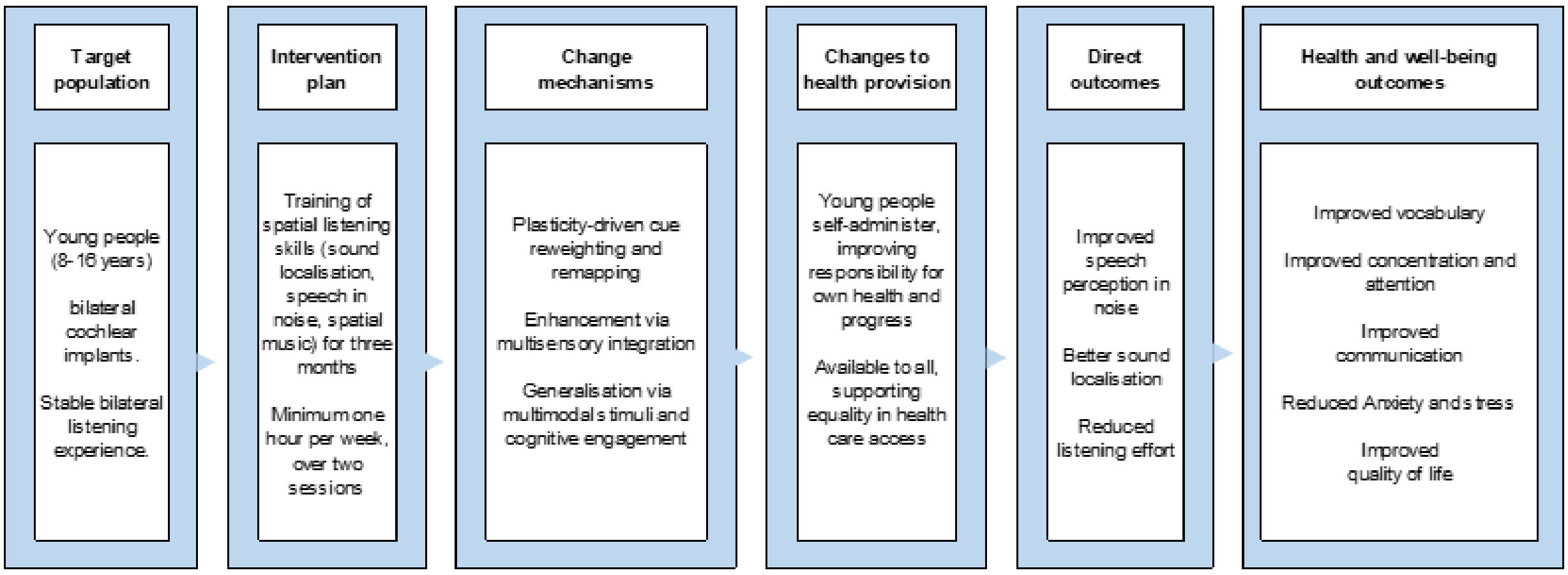

1.4 Games optimisation

To optimise the BEARS training package, we have followed a participatory design whereby stakeholder participants are also co-creators. Children aged 8-16 years old with bilateral CIs attended cycles of workshops, assessed aspects of the three BEARS games and reflected on their experiences which in turn informed changes in the game implementations. The cycles were customised to focus on the elements that the participants considered important. CI users have had the opportunity to give feedback on visual elements of the games or the configuration and settings. Clinicians have also played a key role in the project by assessing the BEARS training package and advising on the usual care pathway across UK CI centres in preparation for the clinical trial. Workshop participants have agreed that the training package are appropriate and ready to be used. The main areas of modification have been the variety of scenarios to cover age range and interests, the number of levels of complexity to ensure small improvements were measurable, feedback and reward schemes to ensure positive reinforcement and the additional implementation of an iPad for those who had difficulties with the headsets [60]. Participants have also provided feedback on different types of headphones which informed our decision on the equipment to be purchased and have helped us examine communication issues in online settings.

1.5 Development of outcome measures

Outcome measures that have been previously developed and validated within this programme grant are:

- The SSiN-VA test. Bizley et al. developed the ‘Spatial-Speech-Test’ [61] to simultaneously assess speech-in-noise perception and relative localisation, thereby increasing cognitive load and more accurately reflecting difficulties faced in everyday listening. This test has been adapted for virtual presentation [10, 62] .Our ‘Technology Review’ PPI group found that this test better reflected their everyday hearing challenges and the listening effort required in noisy environments. Therefore, the Spatial-Speech-Test will be the primary outcome measure. We will also include a standard outcome measure; Spatial Adaptive Sentence Lists (Sp-ASL) [8]. Age-equivalent data for normal-hearing listeners will be obtained for both measures.
- The York Binaural Hearing-Related Quality of Life questionnaire (YBHRQL) was created to measure aspects of quality of life that are sensitive to changes in bilateral hearing [63] and can inform economic evaluations of relevant interventions. It has been validated for adult CI users [64]. We

are currently adapting the YBHRQL for teenagers with CIs and creating a preference-based scoring system so that it can be used to evaluate the cost-effectiveness of BEARS

- The Client Service Receipt Inventory (CSRI) to collect information about the use of NHS services by patients and clinicians [65].
- Qualitative interview topic guides using feedback from children and teenagers

1.6 The clinical trial

Building on the completed components of the BEARS programme above, a randomised clinical trial will now assess the effectiveness of the BEARS training package.

The overall aim of the trial is to determine whether using the spatial-listening training delivered via the BEARS training package for 3-months alongside usual care compared to only receiving usual care improves speech-in-noise perception, hearing experiences, vocabulary and quality of life in young people with bilateral CIs.

1.7 Explanation for choice of comparators

The comparator arm is usual care. This reflects usual UK practice/routine rehabilitation/standard of care for young people with bilateral CIs. A usual care protocol has been defined as part of the project after group discussions with CI centre clinicians (audiologists and rehabilitationists) and teachers of the deaf.

1.8 Objectives

The primary objective of the BEARS study is to determine whether the use of spatial-listening training for 3-months delivered via the BEARS training package in addition to usual care, when compared to usual care alone improves spatial speech-in-noise perception when assessed at three months (accounting for baseline).

The secondary objectives of the BEARS study are to determine whether the use of spatial-listening training for 3-months delivered via the BEARS training package in addition to usual care, when compared to usual care alone:

i. Improves spatial speech-in-noise perception when assessed at twelve months (accounting for baseline).
ii. Improves localisation abilities when assessed at three and twelve months (accounting for baseline)
iii. Reduces listening effort when assessed at three and twelve months (accounting for baseline)
iv. Increases vocabulary age when assessed at three and twelve months (accounting for baseline)
v. Improves quality of life when assessed at three and twelve months (accounting for baseline)
vi. Is cost effective when assessed at three and twelve months (accounting for baseline)
vii. Improves perceived benefits of everyday listening when assessed at three and twelve months (accounting for baseline)
viii. Improves music listening experiences when assessed at three and twelve months (accounting for baseline)

1.9 Trial Design

BEARS is a UK multi-centre, unblinded randomised controlled, phase III superiority trial. Participants will be randomised on a 1:1 ratio.

1.10 Risk Benefit Assessment

BEARS is a low-risk trial. It is a non-CTIMP trial, where the MHRA have confirmed that they do not consider the BEARS training package to be within scope as a medical device.

The documented potential risks associated with the trial's intervention are: injury, pain, discomfort, prolonged dizziness, imbalance or nausea following use of the VR head mounted display These are documented in the Oculus for business safety guide. By having eligibility criterion for potential participants and instructions on how and when to use the device, these risks are greatly reduced. By instructing the participants and their parents/legal representative to use the VR device whilst sitting down, it is anticipated that the risk of injury associated with the VR head mounted display will be reduced. If symptoms of pain, discomfort, prolonged dizziness, imbalance or nausea are being reported, the participant is able to complete the intervention using an iPad with headphones.

The paediatric participant population for the BEARS trial is a higher risk group. Due to the current CI clinical pathway participants approached to be recruited onto the trial will be well known to the auditory implant clinical team and considered suitable to take part in a clinical trial. Assessments will be completed by appropriately trained and delegated staff.

It is considered that the benefits of the BEARS training package outweigh the aforementioned potential risks.

2 Selection of Sites/Investigators

2.1 Site Selection

The Chief Scientific Investigator and Chief Investigator have overall responsibility for site and investigator selection and have delegated this role to CCTU. All sites will have their feasibility for participation in the trial reviewed and assessed in line with CCTU SOPs to ensure sufficient staffing and resource.

2.1.1 Study Setting

BEARS will take place across selected UK paediatric auditory implant clinics.

2.1.2 Site/Investigator Eligibility Criteria

To participate in the BEARS trial, investigators and trial sites must fulfil a set of criteria that have been agreed by the Chief Scientific Investigator and Chief Investigator and BEARS Trial Management Group (TMG) and that are defined below.

Eligibility criteria:

1. A named clinician is willing and appropriate to take Principal Investigator responsibility
2. Suitably trained staff are available to recruit participants, conduct assessments and enter data

Trial sites meeting eligibility criteria and that are accepted by the Chief Scientific Investigator/Chief Investigator /TMG as being suitable to recruit to the trial will be issued with the BEARS document set so that an assessment of capacity and capability can be made. All sites will confirm capacity and capability, and a site initiation visit (SIV) will be performed prior to site activation.

2.1.2.1 Principal Investigator's (PI) Qualifications and Agreements

The Principal Investigator(s) must be willing to sign a Principal Investigator Declaration (Schedule 6 of the Clinical Trial Site Agreement) to comply with the trial protocol (confirming their specific roles and responsibilities relating to the trial, and that their site is willing and able to comply with the requirements of the trial). This includes confirmation of appropriate qualifications (provision of an up-to-date CV), familiarity with the appropriate use of the interventional equipment and agreement to comply with the principles of Good Clinical Practice (GCP). The PI must agree to permit monitoring and audit as necessary at the site, and to maintain documented evidence of staff who have been delegated significant trial related duties.

2.1.2.2 Resourcing at site

- The investigator should demonstrate potential for recruiting the required number of suitable participants within the agreed recruitment period.
- The investigator should have sufficient time to properly conduct and complete the trial within the agreed trial period.
- The investigator should have available an adequate number of qualified staff and suitable facilities for the anticipated duration of the trial in order to conduct the trial properly and safely.
- The investigator should ensure that all persons assisting with the trial are adequately informed about the protocol, the intervention, and their trial-related duties and functions.

- The site should have sufficient data management resources to allow prompt data return to the CCTU (refer to the BEARS CCTU Data Management Plan for timelines).

2.2 Site approval and activation

Site training will be performed prior to the activation of each site and will include all processes for the trial including but not limited to protocol training, data management procedures, procedures for handling of the intervention, adverse event reporting procedures and frequency and expectations for monitoring visits. A log of Site Initiation Visit attendees will be kept in the Investigator Site File (ISF) as a record of participants present. The Visit may occur in person or via Videoconference as outlined in the BEARS CCTU Quality Management and Monitoring Plan (QMMP).

The trial manager or delegate will notify the PI in writing of the plans for site activation. Sites will not be permitted to recruit any patients until the local Research and Development department has given capacity and capability, after which a letter for activation has been issued by CCTU. On receipt of the signed Clinical Trial Site Agreement (including the signed PI Declaration), completed delegation of responsibilities log and staff contact details, the Trial Manager or delegate will complete the green light process and issue written confirmation of site activation to the site PI.

The site must conduct the trial in compliance with the protocol as agreed by the Sponsor and which was given favourable opinion by the Ethics Committee (EC). The PI or delegate must document and explain any deviation from the approved protocol and communicate this to the trial team at CCTU.

A list of activated sites may be obtained from the Trial Manager.

3 Selection of Participants

There will be **NO EXCEPTIONS** (waivers) to eligibility requirements at the time of trial entry. Questions about eligibility criteria should be addressed PRIOR to attempting to enrol/randomise a participant.

The eligibility criteria for this trial have been carefully considered and are the standards used to ensure that only medically appropriate participants are entered. Participants not meeting the criteria should not be entered into the trial for their safety and to ensure that the trial results can be appropriately used to make future treatment decisions for other people with similar diseases or conditions. It is therefore vital that exceptions are not made to these eligibility criteria.

Participants will be considered eligible for enrolment in this trial if they fulfil all the inclusion criteria and none of the exclusion criteria as defined below.

3.1 Participant Inclusion Criteria

1. Participant is a simultaneous or sequential bilateral cochlear implant user*, who either has:
    a. Congenital severe/profound bilateral sensorineural hearing loss and have received at least one implant ≤ 36 months of age.
    b. Progressive or acquired severe/profound bilateral sensorineural hearing loss (no age at implant restrictions for these patients).
2. Participant has stable programmes (defined as no longer using progressive programmes to work through).
3. Participant has had at least two usual care checks/clinical appointments with stable aided levels (+/- 10 dB across 500Hz-4kHz) and no progressive maps to still work through, if they have had re-implantation of internal implant devices.
4. Participant is aged 8-16 years, inclusive.

*(a bilateral CI user is defined as a patient who uses both CI processors for a minimum of 6-hours per day over a month)

3.1.2 Guidance on categorising a severe to profound sensorineural hearing loss

A severe/profound sensorineural hearing loss can either be diagnosed from hearing tests (diagnostic or behavioural) or through a patient's clinically assessed functional hearing abilities, which would make them a cochlear implant candidate.

3.2 Participant Exclusion Criteria

1. Participant (or parent/legal representative) does not speak/understand English sufficiently to undertake assessments.
2. Participant has an intellectual disability at a level that would prevent their ability to understand the trial the intervention or assessment questions.
3. Participant has a comorbid condition impacting ability to participate in the intervention and/or outcome assessment.
4. Participant has an audiological profile impacting ability to participate in the intervention and/or outcome assessments.
5. Participant is actively participating in other trials that may affect hearing outcomes or impact their ability to participate in the intervention.

6. Participant is currently or anticipated to receive treatment and/or intervention that may affect hearing outcomes or adapt implant settings/programming.
7. Participant is refusing to consent to trial activities/protocol.
8. Participant is awaiting reimplantation following device failure or infection.
9. Participant is a non-user of one or both implant processors (i.e., must use both processors for a minimum of 6 hours per day over a month).
10. Participant is a fulltime boarder at a boarding school
11. Participant has unresolvable issues found in device checks that render one of the implants unusable.
12. Participant is pregnant.
13. Participant has a diagnosis of epilepsy or history of seizures of any kind.

3.2.1 Person of Childbearing Potential

One of the participant inclusion criteria for the BEARS trial is that they must be between 8-16 years old (inclusive). This includes people of childbearing potential. Whilst the intervention does not pose a risk to the development of the foetus, adverse events experienced during use of the intervention equipment (e.g. notifiable or serious adverse events, as discussed in Section 6) could potentially do so.

If a participant using the VR head mounted display becomes pregnant during the 3-month intervention, they will instead be provided with an iPad and headphone to use for the remainder of the training period instead. This will substantially reduce this risk to the development of the foetus or child by mitigating the potential for falling while using the BEARS training package.

3.3 Recruitment

A revised total of 272 participants will be recruited to this trial, 136 in each intervention group, over a period of 3-years, which will include a 12-month internal pilot (as described below).

Participants will be recruited from UK CI Centres with expertise in bilateral cochlear implantation and covering geographical locations representing a diverse group of patients.

Most of the proposed centres have collaborated on previous large-scale studies that have successfully reached recruitment targets and high levels of retention: the British CI Group (BCIG) National Service Evaluation on Adult CI Outcomes [66] and the FOUNDATION Study. However, additional centres may be approached to participate in the event of site activation/recruitment challenges.

The study sites will be Cochlear Implant centres. Local trial site team members will be staff working within audiology/implant centre services, with extra support provided by the central research team where required.

The Cochlear Implanted Children's Support (CICS) group, British Association for Teachers of the Deaf (BATOD) and BCIG have agreed to support the BEARS trial by encouraging active recruitment across UK Implant Centres.

The trial will be advertised at each site (using an ethically approved trial poster), at BCIG meetings as well as on public domain websites including the BEARS website and NIHR Be Part of Research, and across the British Association of Teachers of the Deaf (BATOD) website, social media and forums. Potential participants and their parent/legal representative will be provided with the BEARS trial

approved Patient Information Sheet (PIS) through their Implant Centres in the UK, prior to screening for eligibility.

Participants will receive gift vouchers upon completing each of the appointments, as follows:

Baseline visit: £10
Three-month follow-up: £20
Twelve-month follow-up £20

Recruitment will be monitored regularly to identify any barriers to recruitment, which will include an internal pilot with a specific STOP/GO criteria.

The internal pilot will be conducted for the first 12 months of recruitment, with the target of recruiting 25% of the participants.

- If the number recruited is below this target, this will be discussed with the Programme Steering Committee (PSC).
- If <70% of the target is achieved, we will consider the study unfeasible.
- Retention rates, with a target of >80% for the primary outcome, are considered to be the minimum acceptable.

Options to boost recruitment may include a communication/awareness campaign throughout and support organisation websites, and through implant centres.

The Internal pilot will also explore engagement and retention (at 3, 6, 9 and 12-months) and will minimise impact of teething problems to the trial. The remainder of trial recruitment will continue seamlessly upon confirmation of recruitment and retention targets have been achieved. Any required adjustments will be incorporated into the remainder of the trial, with the submission of amendments where required.

Recruitment rates will be reported over the trial set-up and recruitment period on a regular basis to the BEARS Trial Management Group (TMG), Programme Steering Committee (PSC), Independent Data Monitoring and Ethics Committee, alongside the funder. Any concerns regarding the rate of recruitment will be discussed as soon as possible with the relevant programme committees and appropriate strategies implemented where recruitment rates fall below an acceptable level / remedial actions will be put in place if any concerns arise.

3.4 Re-enrolment and Co-enrolment Guidance

All participants will only be enrolled once into the BEARS trial. The site investigators will be responsible for checking the patient notes against the screening/enrolment log to ascertain whether potential participants are already enrolled in the BEARS trial. The online randomisation service used for this trial, (www.sealedenvelope.com) will also prohibit the same participant from being randomised twice.

Participants cannot be concurrently enrolled in any other interventional trial that may affect hearing outcomes or implant settings/ adapt implant programming. Co-enrolment in observational trials is acceptable.

3.5 Screening Procedures and Informed Consent/Assent

Potential participants will be identified through pre-screening measures. This will be completed at the implant centre, where they are receiving usual care. The clinical team will review potential participant's clinical notes, evaluating their implant history and demography.

Pre-screening will be documented on a pseudonymised pre-screening log, produced by the CCTU. This log will document the reasons why patients were not eligible for the trial. Following pre-screening, the clinical implant centre research team should contact those considered eligible for the trial. A copy of the appropriate format of the BEARS PIS should be provided to both the patient and their parent/legal representative at one of the following times:

i) during a routine clinic visit.
ii) in the post or via email alongside routine appointment letter.
iii) following briefing of the potential participant and their family by the clinic team about the trial (e.g., letter, telephone call, email).

Information will be presented to potential participants and their parent/legal representative in a format appropriate to their level of understanding. An age-appropriate PIS that describes the details of the trial, trial procedures, and risks in simplified form will be provided to children who have the capacity to provide informed assent. The PIS should be provided to potential participants and their parents/legal representatives in advance of the online consenting visit (Visit 1). They must be given sufficient time to read and understand the PIS (minimum of 24 hours) and be given the opportunity to ask questions and discuss the aims, methods, benefits, and potential hazards of the trial, as well as the optional qualitative interviews as part of the assessments for the sub-studies, outlined further in section 17.1, before providing electronic informed consent and/or assent.

Following HRA guidance, informed consent will be provided by the parent/legal representative unless the patient is 16 years old or over. If the participant is 16 years old or over, they will provide consent alongside their parent/legal representative. Patients under the age of 16 will provide assent to take part in BEARS alongside their parent/legal representative's consent.

Participants who are 16-years old and over but unable to consent for themselves will not be recruited onto the trial.

The method of informed consent/assent for the BEARS trial will be e-consent via the Sealed Envelope system.

Informed consent will be obtained by the Principal Investigator or delegated member of the local site trial team. The person taking consent/assent will need to be GCP trained, suitably qualified and experienced, and have been delegated this duty by the PI on the delegation log. A copy of a valid GCP certificate for individuals taking consent must be kept in the investigator site file.

During the consent process, it will be made completely and unambiguously clear that the parent/legal representative of the child is free to refuse to participate in all or any aspect of the trial, at any time and for any reason, without incurring any penalty or affecting the treatment of their child. Assent forms do not substitute for the consent form signed by the participant's parent/legal representative.

A PDF copy of the e-consent/assent forms must be kept in:

1. the participant's medical records*,
2. in the site's trial ISF* and
3. a copy given to the participant and parent/legal representative.

* For the Randomised Control Trial only

A letter should be sent to the GP informing them of the trial and the participant's involvement in it.

The following PIS's will be made available for the different age groups:

- PIS for parents/legal representatives
- PIS for children aged 16 years old
- PIS for children aged 11-15 years old
- PIS for children aged 8-10 years old

The following Informed Consent forms (ICF) or Assent forms will be made available for the different age groups:

- ICF for parents/legal representatives
- ICF for children aged 16 years old
- Assent Form for children under 16 years old

The PIS and ICF/Assent Forms will be approved by the ethics committee prior to their use.

Consent will be re-sought if new information becomes available that affects the participant's consent/assent and/or parent/legal representative's consent in any way. This will be documented in a revision to the PIS, submitted to REC as an amendment, and the participant and/or parent/legal representatives will be asked to complete the e-consent process again.

Consent will also be re-sought if a child's carer changes. Children under 16 must be asked to assent, and children achieving their 16th birthday whilst in trial should be consented. Participation (or continued participation) in the trial must be refused if consent/assent is not given.

A copy of the approved PIS and ICF/assent forms are available from the BEARS CCTU trial team.

NB. Informed consent/assent to enter and be randomised into the trial must be obtained from participants and their parents/legal representatives **BEFORE** any trial-specific or sub-study procedures are performed. The only procedures that may be performed in advance of informed consent being obtained are those that would be performed on all patients in the same situation as usual standard of care.

As part of the BEARS trial. We will also be asking the clinicians conducting the BEARS trial at the implant centres to complete questionnaires and be interviewed about their views on the BEARS intervention. This is further detailed in section 17.2 of the protocol. Prior to completing the questionnaire or being interviewed clinicians will be provided with an approved PIS/ICF to complete.

Section 17.1.8 details the screening and consent procedures for the sub-study, qualitative interviews.

3.5.1 E-Consent procedure

Informed consent will be obtained via secure encrypted e-consent module/facility offered via the Sealed Envelope clinical trial services website. It will be obtained in real time, during the online screening appointment prior to any trial related procedures being completed.

Prior to the screening appointment the local site trial team will manually register the patient's parents/legal representative email address into the Sealed Envelope e-consent system. This will generate a unique link, to access the e-consent module, which is then emailed to the parents/legal representative.

For e-consent to be completed, the patient and their parents/legal representative name and signature must be provided. The signature panel works with a mouse or touchscreen. A signature panel is present on the form in line with the HRA recommendations that eSignatures that involve tracing the participant's handwritten signature using a finger or stylus or biometric eSignatures. These allow direct comparison with eSignatures/wet-ink signatures used previously for audit purposes or GCP inspection.

The patient and their parents/legal representative are asked to check the box to make the declaration that they will electronically sign their consent on saving the form. On submitting the signed consent form the patient and their parents/legal representative are shown the form with their completed answers and signature.

Once the patient and/or their parent/legal representative sign the consent form, an email is generated for the principal investigator, or delegated member of the clinical trial team, allowing them to countersign the consent form. A fully executed PDF is sent to all parties, and is stored within Sealed Envelope. The copy within Sealed Envelope is only accessible to the site investigators. All data will be removed from Sealed Envelope once the BEARS trial has closed and the database locked.

The consent process should be documented in the participants medical records. With the consent of the participant, a letter should be sent to the General Practitioner (GP) informing them of the trial and the participant's involvement in it.

Only investigators, delegated the task of consent can view and check the e-consent forms and view the participants and their parents/legal representative's email address and mobile phone number. This information is encrypted. Signatures are timestamped by the Sealed Envelope randomisation software and cannot be altered. All actions involved in the e-consent process are attributed to a used and recorded in the software audit trail.

3.6 Randomisation of Participants

Participants will be randomised on a 1:1 ratio to either the BEARS or usual care intervention arm – further details are provided in Section 8.2.2.

Randomisation will take place during the Baseline appointment (visit 2b) once the outcome assessments have all been completed. The PI or delegate will re-confirm eligibility, within the SealedEnvelope.com secure online randomisation system. This is done by going through the inclusion/exclusion criteria and making sure that the participant meets the eligibility criteria for the trial.

Docusign Envelope ID: 5CA44ABD-F65C-8DE8-80E3-4572550DF9E0

Once the participant has been successfully randomised onto the study, the enrolment of this participant must be documented within the CCTU provided enrolment log.

Delegated staff at participating sites will be provided with a secure login to the SealedEnvelope.com website, according to their role in the trial. The randomisation result will be shown directly online as an intervention allocation, with an email confirmation sent to the user and to the CCTU trial team.

4 Trial Intervention

4.1 Introduction

The participants will be randomised on a 1:1 ratio to one of two intervention arms:

1. Three months of spatial-listening training delivered by BEARS alongside Usual Care. The BEARS training is for a minimum of 1 hour per week, split across at least two 30-minute sessions.
2. Usual care

4.2 Arm A

4.2.1 Both EARS training package (BEARS) and Usual Care

BEARS is a compilation of electronic videogames designed specifically for young people with bilateral CIs. The hardware is either:

- A Head Mounted Display Device (Oculus Quest 2),
- Or an iPad with headphones, for those participants with a smaller head circumference or with reported balance conditions. The headphones that will be used are the AKG 240 studio headphones.

The BEARS training package trains sound localisation using speech and music. BEARS comprises three games addressing different hearing functions: speech-in-noise perception, music listening and sound-source localisation.

Each game is based on an audio-visual task performed through a virtual-reality interface. Players are automatically guided through on-screen visual prompts to support the gameplay with feedback given on their performance. Players progress through levels of increasing difficulty.

The three games are:

1. **Target training game**: Uses audio-visual presentation through headphones and either a mobile phone in a virtual-reality headset or a hand-held tablet. Initially developed to train normal hearing listeners to localise sounds [62], it has subsequently been adapted for CI users. Through a series of increasingly difficult levels, the player is trained to localise sounds relying initially on audio-visual cues. The visual signal disappears gradually as the levels increase, transforming the task into a purely auditory challenge. Stitt et al [62] have shown how virtual auditory displays can be used for creating training environments where users learn to localise sounds using modified localisation cues. The use of audio-visual stimuli helps with task familiarisation in the lower levels, and the gamification approach contributes to improving engagement and attainment.

2. **Speech-in-Noise training game**: Follows many of the same principles as the localisation game it stems from the principle that computer-based training of word perception in noise can improve speech perception [67]. Players will be introduced to virtual scenarios simulating everyday situations, e.g., café, restaurant, classroom. A series of increasingly difficult speech recognition tasks will be presented, challenging the players to interact with the environment (e.g., rotate their

head), localise the speaker and identify the words, using a range of background noises and reverberant acoustic conditions.

3. **Spatial music game**: This is based on the Musiclarity web-application [68] created within the 3D Tune-In project. The game aims to enhance perception and localisation of musical instruments and lyrics in a range of immersive and interactive soundscapes. This game has been developed together with the company Reactify [69], a UK-based company with extensive experience in developing sound applications and interactive music installations, who was involved in developing the original platform.

The BEARS training package design allows for the training to be self-administered, played anywhere and at any time. There is no limit to the frequency of use of the BEARS training package. During each session of use (minimum 2x 30-minute sessions once a week), all three games will need to be played. The device will guide participants through the three games.

The participants will also be provided with a training diary to document any issues they have with the BEARS training package hardware or software, and also to document when the games have been played, helping to ensure the minimum training requirements have been complied to. The BEARS device will automatically log progression and frequency of use, and the player's performance scores for later analysis. No sensitive or personal data is saved onto the device. Once the 3-month intervention has been completed and the device has been returned to the clinic, all data will be downloaded from the device and then deleted.

Note that technical support will be organised via the participant's local implant centre. This will include replacement of equipment in the event of hardware/software failure (see BEARS Device Manual of Operations for details).

The site research teams will be provided training on all aspects of the BEARS training package intervention prior to site activation. This training will be completed during the Site Initiation Visit. Site research teams will be given information and practical training on how to use the BEARS training package using both the head mounted display and the iPad with headphones. They will also be given information on how to troubleshoot issues that could occur with the devices or BEARS training package and who to contact if they cannot solve the participant's problem.

To use the BEARS training package, all participants randomised into the BEARS intervention arm will need an extra program added onto their CIs. This is further detailed in the site training. Participants will need to switch to this program when using the BEARS training package and then return to their everyday program afterwards.

The BEARS intervention will be given in conjunction with Usual Care, which is further described in Section 4.3.1.

4.2.2 Regulatory Status

BEARS is a non-CTIMP study. This is on the basis that the MHRA has confirmed that they do not consider the BEARS software to be a medical device.

4.2.3 Management (Dispensing & Storage) of intervention equipment

Each implant centre will be responsible for the storage, distribution, return and reuse of the BEARS intervention and testing equipment. Storage should be in a locked cabinet or research office. The BEARS intervention equipment will be handed over to sites during the site initiation visit.

The BEARS Equipment log held at each site must record the distribution and return of all BEARS intervention equipment. The log will record the total number of kits at site (e.g. recording kits total/kits dispensed including headset ID cross reference with study PIN).

Participants randomised to the BEARS training package arm will have the trial equipment dispensed and an equipment training session as part of visit 2b.

BEARS equipment will be provided to participants randomised to the BEARS training package arm via their implant centre as follows:

- BEARS equipment and training provided as part of visit 2b
- Step-by-step BEARS VR User guide
- The Oculus Quest 2 safety manual

Following the 3-month intervention period for those randomised to the BEARS arm, participants should return their equipment upon attendance at the 3-month follow-up visit (Visit 3b).

Equipment for the BEARS intervention once returned will have all data downloaded and the device will be reset remotely (as detailed in the BEARS Manual of Operations) and cleaned in accordance with Trust infection control procedures prior to allocation to new participants. The BEARS equipment may be reallocated between sites, based on need at CCTU request only.

Sites will record details of device allocation and return on the BEARS Equipment site log, noting the participant's identification number and device identification codes. This information will also be documented within the trial database via capture on Case Report Forms (CRFs). Sites will use the BEARS Technical Support log to record all instances where a participant using the BEARS equipment requires technical assistance with the device's hardware or software, noting the participant identification number and the following details:

- Number of times site contacted for assistance
- Description of technical assistance required
- Who assisted participant e.g. job role?
- How long did the support take?

4.2.4 Interruptions and Discontinuations of the Trial Intervention

If a participant in the BEARS intervention arm is unable to use the Head Mounted Display device for any reason, they will be provided with an iPad and headphone alternative. Where a participant is unable to use either the Head Mounted Display device or iPad with headphones, the details of this should be documented for each participant within the trial database on the appropriate CRF.

Participants in the BEARS intervention arm who report serious adverse events which are deemed related to the trial intervention may continue with the intervention, if consenting to, at the discretion of the site PI. Where serious adverse events continue to be experienced, the participant, their

parent/legal representative, and investigator at site should decide on whether to stop the trial intervention. Where the decision is taken to stop the intervention, the UCL CCTU should be informed within 24 hours. If the trial intervention is stopped, the participant should continue to be followed for the remainder of the trial duration unless they explicitly withdraw their consent for follow-up. More details about this process are provided in Section 6.

If a participant on the BEARS intervention experiences an interruption/discontinuation associated with either/both CIs, requiring implantation or device setting alteration, they will discontinue the trial intervention and remain in trial for follow-up (as per Intention to Treat analysis principles).

If participants are not able to use the BEARS intervention as intended for any reason not associated with their CIs, despite all mitigating efforts, the participant should continue with follow-up assessment, as per the intention to treat analysis.

4.3 Arm B

4.3.1 Usual Care

Usual care describes the routine rehabilitation received by participants via their implant centre. This is an annual review appointment with the patient and their CI specialist clinician from the implant centre. This contact could be face-to-face, virtual video consultation, questionnaire, or be cochlear remote care checks.

As a minimum this review will check the following:

- Microphone covers changed
- Reported or recorded device use
- All external and internal equipment working (known through no reported or recorded degradation in hearing ability)

During the appointment, the specialist CI clinician would establish if there were any concerns regarding the CI functioning and the patient's rehabilitation programme. They will then follow local protocols to make any repairs or adjustments to the device and manage additional support and contact as required.

Between the annual review appointments patients can attend the implant centre for CI repair appointments or have spare equipment posted from the implant team or implant company (if a contract is in place for this). This will enable their CIs to keep working optimally within BCIG quality standards (BCIG QS). There is no limit to the level of contact between the patient or their family/legal representative and the implant centre, in line with BCIG QS. Any contacts will be recorded in the patient's medical notes and appropriate sections of the CRFs. During enrolment on the trial, no unnecessary upgrades should be carried out of either CI processor.

4.3.2 Interruptions and Discontinuations

If a participant randomised to the usual care intervention experiences an interruption or discontinuation associated with either/both CIs requiring reimplantation or alteration to the settings on the CI they should remain in the trial as per the Intention to Treat analysis. Where this takes place within the BEARS arm intervention period, training activities should be continued only where possible, under the guidance of the implant centre team.

4.4 Concomitant Care

Treatment for any other conditions should be given as per standard care.

Participants that are eligible for BEARS will **not** be enrolled where they are currently or are anticipated to receive treatment and/or an intervention that could modify their CI settings or affect hearing outcomes.

4.5 Blinding Considerations

It is not possible to blind the participant, their parents/legal representative, or the trial research teams to intervention group once it has been allocated, therefore this will be an unblinded randomised clinical trial.

4.5.1 Other Considerations

4.5.1.1 Qualitative sub-study

In order to avoid bias the baseline qualitative interview will be completed before the participant is informed of which trial arm they have been randomised to. The three-month qualitative interview will be completed before the three-month follow-up clinic appointment. This is so that the trial arm or results of the outcome assessments do not influence the interview responses.

4.6 Protocol Intervention Discontinuation

In consenting to BEARS, participants are consenting to either arm of the trial intervention, trial follow-up and data collection. However, a participant can stop the trial intervention early or stop trial participation early for any reason include the following:

1. Any change in the participant's condition that in the clinician's opinion justifies the discontinuation of participation.
2. Unacceptable serious adverse event deemed related to the BEARS intervention.
3. Inter-current illness that prevents continuing the trial intervention/follow-up.
4. Withdrawal of assent/consent for participation by the participant or their parent/legal representative.

As participation in the trial is entirely voluntary, the participant or their parent/legal representative may choose to discontinue trial treatment at any time without penalty or loss of benefits to which they would otherwise be entitled. Although not obliged to give a reason for discontinuing their trial treatment, a reasonable effort should be made to establish this reason, whilst remaining fully respectful of the participant's rights.

It should be clear to the patient and recorded in the patient notes what aspect(s) of the trial the participant is discontinuing their participation. These could include:

- Early cessation of further use of the BEARS intervention
- Early cessation from further trial follow-up

Participants should remain in the trial for the purpose of follow-up and data analysis (unless the participant withdraws their assent/consent from all stages of the trial). If a participant ceases follow-up early, refer to Section 5.4 of the protocol. Data on participants who cease follow-up early will be kept and included in analysis.

4.7 Compliance and Adherence

The importance of undertaking the intervention to the minimum requirements (for those in the BEARs intervention arm) and attending all scheduled follow-up visits (including online) until trial completion will be explained to all participants and their parent/legal representative before they consent/assent to take part in the trial.

If a participant fails to attend a follow-up visit, every effort should be made by the site research team to rearrange the visit and to encourage the participant to attend any remaining follow-up visits.

Participants on the BEARS intervention arm will be provided with a training diary to record details of their use of the BEARS training package e.g. how often and for how long. Participants will be encouraged to bring their diaries with them to the 3-month follow-up visit (Visit 3b for site staff to review to assess compliance/adherence to intervention schedule i.e. minimum of 1 hour per week separated into 2x 30-minute sessions. Reasons for non-compliance will be sought and documented within the trial database via the appropriate CRF.

In addition, compliance metrics will be available from the BEARS equipment devices, which document participant use during the intervention duration of 3 months. These measures are:

- Frequency of training package being used
- What games have been played during each session
- How long each of the games were played for
- General performance for each game

4.7.1 Withdrawal of Consent/assent

If a participant and/or parent/legal representative wishes to withdraw consent/assent, this should be documented on the BEARS Withdrawal CRF. Where the participant provides a reason for discontinuation of the intervention and/or follow-up, the clinical research team will record this in the participants' medical records and also the Withdrawal CRF. The CCTU must be notified of the withdrawal by the site team as soon as they become aware of the Withdrawal request. Further guidance can be found in the BEARS Patient Management Plan.

Once consent/assent has been withdrawn, participation in the trial intervention and/or follow-up will cease, but all the information that has already been obtained up until the point of withdrawal will be kept. To safeguard participant rights, only the minimum possible personally identifiable information will be used.

Due to the nature of the trial database and processes in place for auditing and accountability for trial data collection, it is not possible to remove all the data collected on the participant from the trial. Participants will be made aware of this at the time of recruitment.

4.8 Eligibility Criteria for Individuals Performing the Outcome Assessments

The trial staff will be NHS Hospital or University staff working within paediatric auditory implant clinics and will include (but not be limited to) clinicians, audiologists, speech and language therapists and teachers of the deaf specialising in CIs.

5 Assessments & Follow-Up

5.0 Introduction

All participants will be assessed at the following timepoints:

- Baseline,
- 3 months post-randomisation,
- 12 months post-randomisation.

The details of assessments are provided in Section 5.1 Outcomes, and the schedule of when the assessments will be performed are provided in Section 5.2 Participant Pathway.

Note the permissible periods of time – each as counted from the point of randomisation – within which the visit may take place (visit window) are detailed on the third row of the table in Section 5.2. Where a trial visit takes place outside of each window, it will constitute a deviation from this protocol.

5.1 Outcomes

5.1.1 Primary Outcome

The Primary outcome for the BEARS trial is the difference between the intervention groups in speech-in-noise perception score (% correct overall task) at 3 months, accounting for the participant's baseline score. This is derived from the spatial speech in noise (SSiN-VA) test.

5.1.2 Secondary Outcomes

5.1.2.1 SSiN-VA test outcomes

- Speech-in-noise perception score (% correct of the overall task) at twelve months accounting for the baseline score.
- Relative localisation score (% correct) at three months and at twelve months accounting for the baseline score.
- Average reaction time (measure of listening effort) for word identification selections at three months and at twelve months accounting for the baseline score.
- Average reaction time (measure of listening effort) for location shift selection at three months, and at twelve months accounting for the baseline score.
- Spatial index for word identification at three months and at twelve months accounting for the baseline score (measuring Spatial Release from Masking (SRM)).
- Spatial index for relative localisation at three months and at twelve months accounting for the baseline score.

5.1.2.2 Spatial ASL test outcomes

- SRT at three months and at twelve months, accounting for baseline (for better ear, worse ear, and average of both).
- SRM at three months and at twelve months, accounting for baseline (for better ear, worse ear, and average of both).

5.1.2.3 British Picture Vocabulary Scale (BPVS) test outcome

- Difference between arms in vocabulary age at twelve months, accounting for baseline vocabulary age.

5.1.2.4 VFS-C questionnaire outcome

Difference between arms in listening-related fatigue score at three and 12 months, accounting for baseline.

5.1.3 Health Economic Outcomes

The economic evaluation will calculate incremental cost per quality-adjusted life-year (QALY) gained by offering BEARS and usual care compared to usual care, from an NHS, Personal Social Services (PSS) and Local Education Provider perspective, over the twelve months of the trial.

Outcomes, along with the amounts of missing data, to be reported as part of this analysis are:

- Resource use in each arm, reported according to suitable categories from the CSRI
- Mean cost per participant of delivering the intervention, in the BEARS arm only
- Mean pathway cost per participant in each arm (i.e., costs from CSRI, plus intervention costs if applicable), adjusted for baseline values
- Mean utility values per participant calculated from the YBHRQL-Y self-complete responses, by timepoint and arm
- Mean utility values per participant calculated from the proxy HUI-3 responses, by timepoint and arm
- Mean utility values per participant calculated from the self-completed CHU-9D, by timepoint and arm
- Mean QALYs over 12-months per participant calculated from the YBHRQL-Y self-complete responses, by arm, adjusted for baseline values
- Mean QALYs over 12-months per participant calculated from the proxy HUI-3 responses, by arm, adjusted for baseline values
- Mean QALYs over 12-months per participant calculated from the self-complete CHU-9D responses, by arm, adjusted for baseline values
- Incremental cost-effectiveness ratio (ICER) for primary health economic analysis (i.e. using QALYs from YBHRQL-Y self-complete responses)
- Incremental cost-effectiveness ratio (ICER) for secondary health economic analysis (i.e., using QALYs from proxy HUI-3 responses)
- Incremental cost-effectiveness ratio (ICER) for secondary health economic analysis (i.e., using QALYs from self-complete CHU-9D responses)

5.1.4 Exploratory Analyses

5.1.4.1 Age effects

No further data will be collected for the age effect analyses but the data regarding the trajectory of the children's speech-in-noise scores (SSiN-VA and Sp-ASLs) relative to normative age range for the speech measures and all other secondary outcome measures (VFS, SDQ & YBHRQL-Y) will be assessed to determine if there is a difference in trajectory over time between the BEARS intervention and Usual care groups. Other age factors will be incorporated into the analyses, these will be chronological-to-vocabulary age as an indicator of baseline delay, age at severe-to-profound deafness and hearing age as an indicator of time of listening experience with CI. Within the analyses we will adjust for socio-economic status, pre-implant hearing, balance between ears. Age effects on the use of BEARS games will also be analysed.

5.1.4.2 Retention of training effects

No further data will be collected for the retention effect analyses. It is considered best practice in training interventions to evaluate if the training effects remain after the intervention period has finished. For this analysis fixed effects of timepoint (baseline, 3, and 12 months) and group (BEARS intervention, Usual care) and a random effect of participant will be used to understand the speech-in-noise outcomes. We will conduct these analyses for SSiN-VA, Sp-ASL, VFS & YBHRQL-Y. Age group, balance between ears, pre-implant hearing and socio-economic status will be adjusted for in the analyses.

5.1.4.3 Impact of degree of balance between ears

For this analysis, the AXP-iBalance application will be used to indicate the degree of symmetry across the two ears. The baseline measure will be adjusted for in exploratory analyses as suggested in previous sections. In addition, the measurements at baseline, 3 months and 12 months will be used as an outcome to determine if there have been any changes in the balance between the ears over time. There will also be a fixed effect of timepoint and group (BEARS intervention, Usual care) and a random effect of participant.

5.2 Participant Pathway

Trial visit number (**symbol denotes that visit is to be completed online*)	1	2a *	2b	3a *	3b	4a *	4b
**Month**	**0**	**0**	**0**	**3**	**3**	**12**	**12**
**Protocol window**				**- 2 weeks<br>+6 weeks**	**- 2 weeks<br>+6 weeks**	**- 2 weeks<br>+6 weeks**	**- 2 weeks<br>+6 weeks**
E-consent	x						
Eligibility assessment and demography information	x						
Randomisation			x				
Implant centre data			x				
Clinical checks of implant processor: CI Usual Care and device reporting			x		x		x
Randomisation notification			x				
Dispense trial equipment (for intervention arm only)			x				
Equipment training session (for intervention arm only)			x				
Return of trial equipment (for intervention arm only)					x		x
Safety reporting					x		x
**Assessments**							
Spatial-Speech-Test (SSiN-VA)			x		x		x
Spatial Adaptive Sentence Lists (Sp-ASL)			x		x		x
British Picture Vocabulary Scale (BPVS) (2b: if not completed in last 6 months)			x				x
AXP-iBalance assessment			x		x		x
**Participant reported outcome measures**							
Vanderbilt Fatigue Scale (VFS-C)		x		x		x	
**Health Economics outcome measures**							
York Binaural Hearing-Related Quality of Life questionnaire for young people/youths YHRQL (self-assessment)		x		x		x	
Health Utility Index (HUI-3) (proxy)		x		x		x	
Child Health Utility 9 Dimensions (CHU9D) (self-assessment)		x		x		x	
Adapted Client Service Receipt Inventory (CSRI): parents, implant centres & local education provider versions		x		x		x	

**Trial visit number (*symbol denotes that visit is to be completed online*)**	**1**	**2a ***	**2b**	**3a ***	**3b**	**4a ***	**4b**
**Month**	**0**	**0**	**0**	**3**	**3**	**12**	**12**
**Protocol window**				**- 2 weeks +6 weeks**	**- 2 weeks +6 weeks**	**- 2 weeks +6 weeks**	**- 2 weeks +6 weeks**
**Additional studies**							
Qualitative survey questions		x		x		x	
Process Evaluation: Strengths and Difficulties Questionnaire (SDQ) (parent/legal representative to complete)		x		x		x	
Process Evaluation: Participant questionnaires			x	x		x	
Process Evaluation: Caregiver questionnaires			x	x		x	
**Researcher focused data collection**							
Process Evaluation: Normalisation Measure Development (NoMAD) questionnaire (clinicians to complete)		x					x
Process Evaluation: Qualitative interviews (clinicians)							x
Process Evaluation: Qualitative interviews					x		
Qualitative interviews		x		x			

5.3 Participants randomised from 1st November 2025

The trial has had a 15-month extension to the recruitment period with participants being able to be recruited onto the trial until 31st July 2026 with the last BEARS follow-up appointment occurring on 31st October 2026. From 1st November 2025 no newly randomised participants will have a 12-month follow-up (due to trial timeline constraints) and will all finish their trial pathway after the 3-month follow-up appointment.

The change in the schedule of assessments for this cohort of participants are detailed in section 5.3.1. For this cohort of participants, the secondary objectives related to the 12-month follow-up appointment will not be assessed.

For any participants randomised onto the trial after 1st November 2025 they will be given the following gift vouchers after each appointment:

- Baseline: £10 gift voucher
- 3-month follow-up appointment: £20 gift voucher

5.3.1 Participant pathway for those randomised from November 2025

Trial visit number (*symbol denotes that visit is to be completed online*)	1	2a *	2b	3a *	3b
**Month**	**0**	**0**	**0**	**3**	**3**
**Protocol window**				**- 2 weeks +6 weeks**	**- 2 weeks +6 weeks**
E-consent	X				
Eligibility assessment and demography information	X				
Randomisation			X		
Implant centre data			X		
Clinical checks of implant processor: CI Usual Care and device reporting			X		X
Randomisation notification			X		
Dispense trial equipment (for intervention arm only)			X		
Equipment training session (for intervention arm only)			X		
Return of trial equipment (for intervention arm only)					X
Safety reporting					X
**Assessments**					
Spatial-Speech-Test (SSiN-VA)			X		X
Spatial Adaptive Sentence Lists (Sp-ASL)			X		X
British Picture Vocabulary Scale (BPVS) (2b: if not completed in last 6 months)			X		
AXP-iBalance assessment			X		X
**Participant reported outcome measures**					
Vanderbilt Fatigue Scale (VFS-C)		X		X	
**Health Economic outcome measures**					
York Binaural Hearing-Related Quality of Life questionnaire for young people/youths YBHRQL (self-assessment)		X		X	
Health Utility Index (HUI-3) (proxy)		X		X	
Child Health Utility 9 Dimensions (CHU-9D) (self-assessment)		X		X	
Adapted Client Service Receipt Inventory (CSRI): parents, implant centres & local education provider versions		X		X	
Qualitative survey questions		X		X	
Process Evaluation: Strengths and Difficulties Questionnaire (SDQ) (parent/legal representative to complete)		X		X	
Process Evaluation: Participant questionnaires			X	X	
Process Evaluation: Caregiver questionnaires			X	X	
**Researcher focused data collection**					
Process Evaluation: Normalisation Measure Development (NoMAD) questionnaire (clinicians to complete)		X			
Process Evaluation: Qualitative interviews (clinicians)					X
Process Evaluation: Qualitative interviews					

5.4 Participant Transfers

If a participant moves away from their local area, making continued follow up at their consenting centre inappropriate, every effort should be made for them to be followed at another participating trial centre. Consent/assent of the participant and parent/legal representative should be taken at the new centre and then a copy of the participant's CRFs should be provided to the new centre. Responsibility for the participant remains with the original consenting centre until the new consent

process is complete. The original site remains responsible for resolving data queries relating to data collection that has occurred prior to the point of transfer.

5.5 Early Stopping of Follow-up

If a participant or their parent/legal representative chooses to discontinue the trial intervention, they should always be followed up providing they are willing, that is, they should be encouraged to not leave the whole trial. If they do not wish to remain on trial follow-up, however, their decision must be respected, and the participant will be withdrawn from the trial completely. The CCTU should be informed of this in writing using the appropriate documentation as soon as they become aware of the participant and/or parent/legal representative's request. Data already collected will be kept and included in analyses according to the intention-to-treat principle for all participants who stop follow up early.

Participants ending trial involvement prematurely may have a negative impact on trial data integrity and the ability to reach sufficient statistical confidence in the stated endpoints, and therefore should be avoided where possible, while remaining respectful of the participant's rights.

Participants or their parent/legal representative may change their minds about stopping trial follow-up at any time and re-consent to participation in the trial. Participants who stop trial follow-up early will not be replaced.

5.6 Loss to Follow-up

Implant centre teams will be encouraged from the outset to ensure that the participant and parent/legal representative is aware of the trial requirements including the intervention and follow-up visit schedules, before they commit to participation. Judgement should be used when maintaining contact with participants and their parent/legal representative.

Where there is a lack of engagement/contact from the participant and parent/legal representative, site teams should make every reasonable effort to re-engage with the participant and parent/legal representative. Please refer to the BEARS Patient Management Plan.

If the participant and parent/legal representative is not contactable or does not arrive for an agreed trial appointment, the site should attempt to contact the family (e.g., by telephone, text, email, or letter) on at least 3 different occasions. If it is not possible to make contact via these methods, the implant centre team should contact the participant's GP.

Only following attempts to contact the participant and parent/legal representative as detailed above, with no response received, should the participant be deemed lost to follow-up.

5.7 Post-trial Care

Post-trial care will be standard medical care (routine rehabilitation).

6 Safety reporting

The principles of GCP require that both investigators and Sponsors follow specific procedures when notifying and reporting adverse events or reactions in clinical trials. These procedures are described in this section of the protocol. **Section 6.1** lists definitions, **Section 6.3** gives details of the investigator responsibilities and **Section 6.4** provides information on CCTU responsibilities.

6.1 Definitions

Definitions of harm of the EU Directive 2001/20/EC Article 2 based on the principles of GCP apply to this trial.

Table 1: Definitions

Adverse Event (AE)	Any untoward medical occurrence in a patient or clinical trial participant using an intervention and which does not necessarily have a causal relationship with this product.
Notifiable Adverse Event (NAE)	Any notifiable untoward medical occurrence in a patient or clinical trial participant using an intervention and which does not necessarily have a causal relationship with this product.
Serious Adverse Event (SAE)	An adverse event, adverse reaction, or unexpected adverse reaction that:<br>• results in death<br>• is life threatening*<br>• requires hospitalisation or prolongation existing hospitalisation**<br>• results in persistent or significant disability or incapacity<br>• consists of a congenital anomaly or birth defect<br>• is another important medical condition***
* The term life threatening here refers to an event in which the patient is at risk of death at the time of the event; it does not refer to an event that might hypothetically cause death if it was more severe (e.g., a silent myocardial infarction)<br>** Hospitalisation is defined as an in-patient admission, regardless of length of stay, even if the hospitalisation is a precautionary measure for continued observation. Hospitalisation for pre-existing conditions (including elective procedures that have not worsened) do not constitute an SAE<br>*** Medical judgement should be exercised in deciding whether an AE or NAE is serious in other situations. The following should also be considered serious: important AEs or NAEs that are not immediately life threatening or do not result in death or hospitalisation but may jeopardise the participant or may require intervention to prevent one of the other outcomes listed in the definition above (e.g., a secondary malignancy, an allergic bronchospasm requiring intensive emergency treatment, seizures, or blood dyscrasias that do not result in hospitalisation, or development of drug dependency).	

6.1.1 Adverse Events

Adverse events include:

- An exacerbation (i.e., increase in the frequency or intensity) of a pre-existing illness, episodic event or symptom (initially recorded at the baseline visit), that is detected after intervention
- Occurrence of a new illness, episodic event or symptom, that is detected after intervention

Adverse events do NOT include:

- Medical or surgical procedures: the condition that leads to the procedure is the adverse event
- Pre-existing disease or a condition present before treatment that does not worsen
- Hospitalisation where no untoward or unintended response has occurred e.g., elective cosmetic surgery
- Overdose of medication without signs or symptoms

6.2 Notifiable Adverse Events (NAEs)

The safety manual for the Oculus Quest 2 documents possible adverse events that could occur when using the VR headset. From this list, the following adverse events should be considered as notifiable to CCTU, **only if occurring when using the headset or 1-hour post-use:**

- Seizures
- Loss of awareness
- Involuntary movements
- Altered, blurred, or double vision or other visual abnormalities
- Persistent dizziness
- Disorientation
- Impaired balance
- Nausea
- Light-headedness
- Discomfort or pain in the head, eyes, or site of CI
- Drowsiness
- Fatigue
- Any symptom similar to motion sickness

No NAEs are reportable for the participants using the iPad to complete the BEARS training package.

6.2.1 Pregnancy

From the time of consent, all pregnancies and suspected pregnancies that occur in female participants must be reported immediately upon the site becoming aware to UCL CCTU using the notification and follow-up of pregnancy form.

Before any additional outcome of pregnancy or pregnancy complications can be reported to UCL CCTU, pregnant participants must be given a copy of the trial pregnancy monitoring information sheet and asked to complete a pregnancy monitoring informed consent form for follow-up in pregnancy. The process should follow GCP, and the processes used to obtain consent for the clinical trial.

If a participant who is randomised to the BEARS intervention arm becomes pregnant during the first three months of trial, they must use immediately discontinue use of the head mounted display and will be provided instead with an iPad to complete the BEARS training package period.

Pregnancy outcomes should be reported to UCL CCTU as soon as notified. Any complications of pregnancy including miscarriage, congenital abnormality or birth defect resulting from the pregnancy must be reported as an SAE to UCL CCTU, within the timelines outlined in section 7.3.2 below.

6.3 Investigator responsibilities

6.3.1 Investigator Assessment

6.3.1.1 Seriousness

When an AE occurs, the investigator responsible for the care of the participant must assess whether or not the event is 'serious' **using the definition given in Table 1** in the section above.

6.3.1.2 Severity or Grading of Adverse Events

The severity of all SAEs and NAEs in this trial should be graded using the toxicity gradings in the Common Terminology Criteria for Adverse Events (CTCAE) Version 5.0 (2017).

Grades for NAEs and SAEs according to the CTCAE are as per Table 2 below.

Table 2: Toxicity Gradings for Adverse Events

Grade	Description
One	Mild; asymptomatic or mild symptoms; clinical diagnostic observations only; intervention not indicated
Two	Moderate; minimal, local or non-invasive intervention indicated; limiting age-appropriate instrumental activities of daily living (ADL)*
Three	Severe or medically significant but not life threatening; hospitalisation or prolongation of hospitalisation indicated; disabling; limiting self-care ADL**
Four	Life threatening consequences: urgent intervention indicated
Five	Death related to AE or AR
**Instrumental ADL refer to preparing meals, shopping for groceries or clothes, using the telephone, managing money etc.*<br>*** Self-care AD refer to bathing, dressing and undressing, feeding self, using the toilet, taking medications and not bedridden.*	

Where no specific grading criteria exist for an event, the event should be graded according to the CTCAE general guidelines outlined above.

6.3.1.3 Causality

The investigator must assess the causality of all NAEs and SAEs in relation to the trial intervention using the definitions in Table 3. There are five categories: unrelated, unlikely, possibly, probably, and definitely related. If the causality assessment is unrelated or unlikely to be related, the event is classified as an 'unrelated NAE/SAE'. If the causality is assessed as possibly, probably or definitely related, then the event is a 'related NAE/SAE'.

Table 3: Assigning Type of NAE/SAE Through Causality

Relationship	Description	Event type
Definitely	There is clear evidence to suggest a causal relationship and other possible contributing factors can be ruled out	Related NAE/SAE
Probably	There is evidence to suggest a causal relationship and the influence of other factors is unlikely	Related NAE/SAE
Possibly	There is some evidence to suggest a causal relationship (e.g., because the event occurs within a reasonable time after intervention). However, the influence of other factors may have contributed to the event (e.g., the participant's clinical condition, other concomitant treatment)	Related NAE/SAE

Unlikely	There is little evidence to suggest that there is a causal relationship (e.g., the event did not occur within a reasonable time after intervention). There is another reasonable explanation for the event (e.g., the participant's clinical condition, other concomitant treatment)	Unrelated NAE/SAE
Unrelated	There is no evidence of any causal relationship	Unrelated NAE/SAE

Note that in all cases above where trial intervention has been discontinued, interrupted or modified, refer to the relevant sections of the protocol for further guidance.

6.3.1.4 Expectedness

If there is at least a possible involvement of the trial intervention, **the Chief Investigator** must assess the expectedness of the event. However, the Sponsor has the overall responsibility for determination of expectedness. A list of expected occurrences resulting from the trial intervention are as follows:

- Seizures
- Fall immediately after use of the VR headset
- Loss of awareness
- Involuntary movements
- Altered, blurred, or double vision or other visual abnormalities
- Persistent dizziness
- Disorientation
- Impaired balance
- Nausea
- Light-headedness
- Discomfort or pain in the head, eyes or site of CI
- Drowsiness
- Fatigue
- Any symptom similar to motion sickness

When the event falls outside of this list, it must be deemed 'unexpected' by the Chief Investigator. If an SAE is assessed as being related to the trial intervention (resulting from administration of any of the research procedures) and unexpected, then the event must be reported to the REC (see Notifications sections of the protocol).

6.3.2 Notifications

Where the SAE is related (that is, it resulted from administration of any of the research procedures), to the study procedures or is an unexpected occurrence (that is, the type of event is not listed in the protocol as an expected occurrence) then it must be reported by the principal investigator or delegate immediately upon knowledge of the event, and always within 24 hours to the UCL CCTU and the Chief Investigator. The UCL CCTU will onward report related SAEs to the GSTT sponsor office.

6.3.2.1 Notifications by the Investigator to CCTU

SAEs and NAEs will be reported using the BEARS Serious Adverse Event Case Report Form. This must be completed by the investigator delegated this responsibility, with attention paid to the grading, causality and expectedness of the event.

In the absence of the responsible investigator, the SAE CRF should be completed and signed by a member of the site trial team and emailed as appropriate within the timeline. The responsible investigator should check the SAE form at the earliest opportunity, make any changes necessary, sign and then email to CCTU. Detailed written reports should be completed as appropriate. Systems will be in place at the site to enable the responsible investigator to check the form for clinical accuracy as soon as possible.

The minimum criteria required for reporting an SAE/NAE are the primary event term, PIN and partial date of birth (month and year), name of reporting investigator and sufficient information on the event to confirm seriousness. Any further information regarding the event that is unavailable at the time of the first report should be sent to the CCTU as soon as it becomes available.

6.2.3.1.1 SAEs

CCTU must be notified of all SAEs considered to be **possibly/probably/definitely related** to the participant's involvement on the BEARS trial immediately when site staff become aware of the event (in no circumstances should this notification take longer than 24 hours). Reported **SAEs that are unexpected and related** to the participant's involvement on the BEARS trial will need onward reporting to the REC within fifteen days of the CI becoming aware of the event.

Investigators should use the above guidance/timelines to notify CCTU of any SAEs occurring from the time of randomisation until 30 days after the participant completes their last follow-up appointment (visit 6b).

6.3.2.1.2 NAEs

All NAEs should be reported to the CCTU from the time of randomisation until the time of the discontinuation of use of the BEARS intervention. NAEs need to be reported immediately when site staff become aware of the event (in no circumstances should this notification take longer than 24 hours).

6.3.2.1.3 Other AEs

Where an AE is considered to be neither 'serious' nor fulfils the requirements of a NAE within the BEARS context should be recorded in the patient's medical records only. Such AEs should **not** be recorded in the trial's CRFs nor reported to the UCL CCTU.

6.3.2.2 Approach to be Used for Notifications by the Investigator to CCTU

Procedures required for NAE or SAE notifications to the UCL CCTU:

1. The NAE/SAE eCRF must be completed on the BEARS central database by the site research team
2. Sites must also email the CCTU to notify them when a NAE or SAE occurs
3. The CCTU trial team, will download a PDF copy of the NAE/SAE. This will be sent to the Chief Investigator for review
4. Follow-up of SAEs/NAEs: Participants must be followed up until clinical recovery is complete, or until the event has stabilised. Follow-up should continue after completion of protocol treatment and/or trial follow-up if necessary. Follow-up forms should be completed on the BEARS central database as further information becomes available
5. Staff should follow their institution's procedure for local notification requirements

Further details can be found in the BEARS Safety Management Plan.

6.3.2.3 Notifications of CI device issues to CCTU

Any faults to either CI, that prevents participants in the BEARS intervention arm from completing one or more of their training sessions or in the Usual Care arm from using their CIs for over a week need to be reported to the CCTU upon identification of the issue by the site team.

Confirmation of any internal CI device failure need be reported to the CCTU within 3-days. These participants will continue to participate on the trial, under the intention to treat analysis (as mentioned in sections 4.2.4 and 4.3.4).

Faults to the CI devices that can be fixed through a replacement of cable/head piece or processor within three days do not need to be immediately reported to the CCTU. It is not considered that this will have an effect on the participants involvement in the trial.

6.4 CCTU responsibilities

The Chief Investigator or a medically qualified and delegated individual will review all SAE/NAE reports received. The causality assessment given by the local investigator at the hospital cannot be overruled; in the case of disagreement, both opinions will be provided in any subsequent reports.

The Chief Investigator or a medically qualified and delegated individual will review the form for expectedness against the events given in Section 6.3.1.4 and provide a Sponsor assessment of expectedness for the purposes of onward reporting. The site team should not perform this function.

CCTU is undertaking certain duties of trial sponsor and is responsible for the reporting of unexpected SAEs to the REC as appropriate, with GSTT Sponsor copied in all correspondence. Note that all NAEs are, by the definitions provided in S6.2 and S6.3.1.4, are all 'expected' with no onward reporting requirement. CCTU will keep investigators informed of any safety issues that arise during the trial.

An Annual Progress Report (APR) will be submitted to the REC within 30 days of the anniversary date on which the favourable opinion was given, and annually until the trial is declared ended.

6.4.1 Urgent Safety Measures

The CCTU or investigator may take appropriate urgent safety measures in order to protect research participants against any immediate hazard to their health or safety.

Where urgent safety measures are taken the CCTU and CIs shall immediately (not later than 3 days from the date measures are taken), give written notice to the REC of the measures taken and the circumstances giving rise to those measures, according to the relevant CCTU SOP. The sponsor will be copied into these correspondences.

7 Quality Assurance & Control

7.1 Risk Assessment

The Quality Assurance (QA) and Quality Control (QC) considerations for the BEARS trial are based on the standard CCTU Quality Management Policy that includes a formal Risk Assessment, and that acknowledges the risks associated with the conduct of the trial and proposals of how to mitigate them through appropriate QA and QC processes. Risks are defined in terms of their impact on the rights and safety of participants; project concept including trial design, reliability of results and institutional risk; project management; risk benefit of the trial; and other considerations.

QA is defined as all the planned and systematic actions established to ensure the trial is performed and data generated, documented and/or recorded and reported in compliance with the principles of GCP and applicable regulatory requirements. QC includes the operational techniques and activities performed within the QA system to verify that the requirements for quality of the trial related activities are fulfilled.

The BEARS Risk Assessment has been reviewed by the CCTU's Quality Management Group (QMG).

7.2 Central Monitoring at CCTU

CCTU staff will review data and other information provided by investigators to identify trends, outliers, anomalies, protocol deviations and inconsistencies. The frequency and type of central monitoring will be detailed in the BEARS Quality Management and Monitoring Plan (QMMP).

7.3 Monitoring

The frequency, type and intensity of routine on-site monitoring and the requirements for triggered on-site monitoring will be detailed in the BEARS QMMP, including any provision for remote or self-monitoring. The QMMP will detail the procedures for review and sign-off of monitoring reports. In the event of a request for a trial site inspection by any regulatory authority UCL CCTU and the Sponsor must be notified as soon as possible.

CCTU staff will review CRF data for errors and missing key data points. The trial database will also be programmed to generate reports on errors and error rates. Essential trial issues, events and outputs, including defined key data points, will be detailed in the BEARS Data Management Plan.

7.3.1 Direct access to Participant Records

Participating investigators must agree to allow trial-related monitoring, including audits, EC review and regulatory inspections, by providing access to source data and other documents as required. Participant consent for this must be obtained as part of the informed consent process for the trial.

7.3.2 Confidentiality

CCTU will follow the principles of the UK Data Protection Act 2018 and subsequent amendments.

The informed consent form and assent forms will carry the participant's name and an appropriate signature, and that of their parent/legal representative these will be retained at the trial site (participant's hospital). The consent forms will only be accessed by UCL CCTU staff for purposes of monitoring the consent procedure at the site.

Participant's data will be collected and kept securely. Confidentiality of participant's personal data is ensured by not collecting participant names and other personally identifiable information on CRFs and receiving only pseudonymised data. At trial enrolment, participants will be allocated a PIN, which will be used on all trial related paperwork sent to UCL CCTU and in the trial database. Any documents (e.g. screening and enrolment logs) linking PIN to participant's personally identifiable information will be kept securely at site; only redacted copies will be sent to Sponsor if requested.

Copies of participant's trial data will be kept at the participating site in a secure location with restricted access. Unless working at a site, CCTU staff will only have access to the data collected on the trial CRFs (i.e. they will not have access to any other personal data) and applicable source data, moreover only staff working on the trial will have access to these data. CCTU copies of CRF data on paper are stored securely in locked filing cabinets at the UCL CCTU office, data stored electronically is held on secure servers, that have restricted access.

The implant centre teams will have access to the participants' medical/implant/educational records (including hospital records) for the purposes of screening for eligibility. Access to these records will only be gained after the participant and parent/legal representative has given consent for this.

Personal data information required for trial co-ordination (e.g. participant and parent/legal representative contact information) will be kept in a separate database to the main trial study data. All electronic information will be kept in encrypted, password protected files stored on implant centre internal networks (including laptops), in line with local NHS Trust policies. In the event of a CCTU or Sponsor led audit or inspection, the authorised individual will also require access to the participant's medical and educational records in the course of their duties.

Please refer to Section 17 for confidentiality considerations for the exploratory/sub-study work.

7.4 Source Data

The investigator should maintain adequate and accurate source documents and trial records that include all pertinent observations on each of the site's trial participants. Source data are contained in source documents and are defined here as all information in original records that are used for the reconstruction and evaluation of the clinical trial. Source documents are the first place where the source data are recorded. These can include hospital records, clinical and office charts, and source data worksheets.

Source data should be attributable, legible, contemporaneous, original, accurate, and complete. Changes to source data should be traceable, should not obscure the original entry, and should be explained if necessary (e.g., via an audit trail). Each data element should only have one source.

For this trial, the majority of datapoints will be recorded directly into CRFs/eCRFs and therefore the CRF/eCRF will be regarded as source data. However, a minority of datapoints will be recorded in the participant's medical notes. In such cases, the medical notes will be regarded as source data. The location of each datapoint will be detailed in the BEARS Data Management Plan.

A Source Data Agreement will be put in place as part of the site activation process with each site. This will define the source documents and the data therein, together with location of the source documents.

All trial data should all be verifiable from source documents, which may include CRFs/eCRFs, paper notes and electronic health records.

7.5 Data Collection and Transfer Methods

Trial specific CRFs will be designed by the BEARS trial team. The approved BEARS CRFs will be provided to all participating sites.

Data collected will need to be entered by members of the clinical research team delegated the task, onto the central trial database electronic CRFs by members of the BEARS trial team working within each research site.

Data may also be entered onto paper Case Record Forms (CRFs) prior to manual entry into the database, though this is an optional step and should be performed only where considered supportive for local site processes.

Trial data will be collected at the time-points indicated in the Participant Timeline (Section 5.2). Data collection will only occur by delegated members of the trial team across participating sites. Trial team members will receive adequate training on the BEARS protocol and outcome assessments used as part of the trial prior to data collection. The PI is responsible for ensuring site staff are adequately trained for the roles and responsibilities they are delegated.

The primary and secondary outcome measure assessments (SSiN-VA and Sp-ASLs) need to be conducted by a healthcare specialist who has had appropriate training in completing paediatric speech tests.

Training on data collection, secure data transfer and storage for site staff listed on the delegation of responsibilities log will be provided at the site initiation meeting(s). Trial staff will receive separate training on data collection, data entry and use of the BEARS bespoke database.

Safety Report Forms will be completed on the BEARS central database as detailed in section 6.3.2.1, Safety Reporting (also refer to trial Safety Management Plan).

Note that in line with GDPR data minimisation principles, the CRFs will not bear the participant's name or initials; instead, participant PIN will be recorded and used for identification with corresponding trial screening/enrolment logs. Partial date of birth will also be recorded and can be used to validate PIN.

7.6 Data Management

The database software provides a number of features to help maintain data quality, including maintaining an audit trail, allowing custom validations on data, allowing users to raise data query requests, and search facilities to identify validation failure and missing data.

Data collection, data entry, queries raised by a member of the BEARS trial team and database lock(s) will be conducted in line with the CCTU SOPs and BEARS Data Management Plan. After completion of the trial, the database will be retained for on-going analyses. The CCTU will retain a copy of the database, which will be password protected and only accessible to members of the BEARS trial team at CCTU, delegated site staff and external regulators if requested. Database users will only be granted permissions to use the database functionality appropriate to their role in the clinical trial.

All data will be handled in accordance with the Data Protection Act 2018, the UK General Data Protection Regulation (UK GDPR) and subsequent updates and amendments.

Central review of the SSiN-VA, Sp-ASL, iBalance and game play and usage assessment will be undertaken by the central trial teams at Cambridge University and Imperial College on behalf of the sites. These datasets are being stored on the University of Cambridge OneDrive.

7.7 Data Storage

Trial data will be stored in a database created specifically for the BEARS trial. This database is hosted by OpenClinica which is validated for 21 CFR Part 11, GCP, HIPAA, GDPR, and Annex 11. The data is stored on secure, GDPR-compliant, cloud-based servers held within UK and EU: https://www.openclinica.com/privacy-policy/.

The randomisation service is hosted by Sealed Envelope LTD. The data is stored on a secure, GDPR-compliant, cloud-based servers held within the EU: https://www.sealedenvelope.com/security/.

These databases are protected by multi-layer firewalls with full data encryption at rest and in transit. The identification, screening, and enrolment logs, linking personally identifiable information to the PIN, will be held locally by the trial site. This will either be held in written form in a locked filing cabinet or electronically in password protected form on hospital computers.

Data that is external to the trial database, such as training and compliance records for those in the BEARS intervention group, and the raw SSiN-VA, Sp-ASL, iBalance application and data logging data will be saved in password protected secure form on institutional computer systems.

7.8 Data Archiving

Once all primary, secondary and health economic and exploratory analyses have been completed the trial data will be archived. Once the trial data has been archived the trial database will be decommissioned and will no longer be available. Any subsequent/ further analysis will be performed using the archived data. This will be within one year of the end of the trial.

The investigators agree to archive and/or arrange for secure storage of BEARS trial materials and records/essential documents for a minimum of 5 years after the close of the trial unless otherwise advised by the CCTU or Sponsor.

7.9 Quality Issues

Quality Issues are issues that can have an impact on patient safety, rights, and well-being; data integrity and/or scientific rigor; and compliance with regulatory requirements; these can be classified as protocol deviations, potential serious breaches, near misses etc.

A protocol deviation is any departure from procedures documented in this protocol, this includes deviations that cannot be predicted. If a protocol deviation is identified the BEARS trial team should be contacted and CCTU's protocol deviation reporting process will be followed.
A 'serious breach' is a deviation from procedures documented in this protocol, GCP or other clinical trial regulations that is likely to affect to a significant degree:

1. The safety or physical or mental integrity of the participants in the trial, or
2. The scientific value of the trial

If a serious breach is suspected the CCTU must be contacted within 1-working day. The CCTU and CI will prepare a report of the serious breach, which will be reviewed by the Sponsor. If appropriate, the Sponsor will report it to the EC committee within 7 days.

8. Statistical Considerations

8.1 Sample Size

The Primary outcome for the BEARS trial is the difference between the intervention groups in speech-in-noise-perception score (% correct overall task) at 3 months, accounting for the participant's baseline score [70].

The original sample size was 153 evaluable participants per group required to detect the clinically important difference between treatment groups of 2.25 percentage points in change in speech-in-noise-perception score (obtained when the speech and noise separated by 30° compared to 0°), with 90% power and at the 5% level of significance (two-sided). The SD of change from baseline is estimated to be 6.05 percentage points, obtained from the Spatial-Speech-Test validation study. Mitigating loss to follow up or other challenges a 20% allowance raised this to a total of 192 participants per group totalling 384 participants.

The revised sample size of a total of 230 evaluable participants is based on detecting the clinically important difference between treatment groups of 2.25 percentage points in change in speech-in-noise-perception score with 80% power and at the 5% level of significance (two-sided), assuming the original SD in change from baseline of 6.05 percentage points. Based on observed withdrawal proportion in the trial, we revised the allowance for loss to follow-up to 15%. The revised required sample size is 136 per group, totalling 272 participants.

8.2 Assignment of Intervention

8.2.1 Randomisation

Participants will be randomised 1:1 to either the BEARS alongside Usual Care or Usual Care alone intervention groups.

8.2.2 Sequence generation

Randomisation will be performed by the PI or delegated member of the trial team at local sites using an independent online randomisation service (www.sealedenvelope.com) that minimises allocation bias within the trial. The use of Sealed Envelope and the specifications of the randomisation system required for BEARS have been discussed with the trial statistician and validated for use. Following confirmation of eligibility and consent, each participant will be randomised using their screening identification number that was allocated sequentially at screening (as detailed in the trial Patient Management Plan).

Participants will be randomised 1:1 using a concealed minimisation process according to the minimisation factors (listed below). This will ensure that balance across the two intervention groups will be achieved. The minimisation algorithm will incorporate a random element to maximise balance in the stratifying variables between the randomised groups.

Minimisation factors will include:

- Sex
- School age group
- School setting
- Congenital deafness, severe to profound hearing loss

- Device allocation

8.2.3 Allocation concealment mechanism

Intervention allocation will be concealed from the participant/family prior to notification, to prevent allocation bias on baseline data collection. This will be facilitated by the use of minimisation.

Allocation concealment (blinding) at site is not possible during the intervention period as the equipment/training aspects of the BEARS intervention will require presenting to the participant/family at the end of their baseline visit (visit 2b). Therefore, participant blinding is considered to have ended upon the formal notification point.

8.2.4 Allocation Implementation

The responsibility for enrolling participants and randomising them to intervention group lies with the PI and other trained individuals (e.g. clinicians/research nurses) named on the site delegation log.

Once a participant has been allocated to an intervention group and following baseline data collection (and qualitative interviews if consented and selected for this), the participant/family should be informed of the intervention allocation by a delegated team member (randomisation notification).

In addition, where allocated to the BEARS intervention group, the site team must ensure that the participant/family are provided with the appropriate equipment and training.

8.2.5 Blinding

The proposed trial will be unblinded. It is not possible to blind patients, clinicians and researchers to the intervention allocation following randomisation.

8.3 Statistical Methods

8.3.1 Statistical Analysis Plan

A detailed statistical analysis plan (SAP), including a full specification of the analysis principles and details, will be drafted as early as possible and finalised prior to the first substantive analysis, following approval by the Program Steering Committee (PSC) and review by the Data Monitoring and Ethics Committee (DMEC).

8.3.2 Interim Analyses

There will be no interim analyses. Monitoring of the safety of the trial will be undertaken by the DMEC which will have untrammelled access to the trial data, and whose work is governed by a separate charter.

8.3.3 Statistical Methods – Outcomes

1. Outcomes to confirm improvements in everyday hearing (SSiN-VA) following BEARS compared to Usual-Care

Primary, secondary and exploratory analyses will be by intention to treat. We will use a linear mixed model to compare the speech-in-noise perception scores between the two arms. The baseline and three-month measures will be included as outcome variables. The fixed-effect of the model will include an indicator variable to identify pre-and post-intervention measures and the minimisation factors as adjusting variables. A random intercept term will be included to account for clustering

within participants, and an additional random intercept will be included to account for clustering by site.

We will fit analogous models with appropriate link functions and error terms for the analysis of the secondary outcomes, speech-in-noise perception at 12 months and BPVS, YBYRQL-Y and VFS at three and 12 months.

2. Outcomes of the clinical trial to confirm improvements in QoL (YBHRQL-Y and HUI-3) following BEARS compared to Usual-Care

Quality of life scores (also called "utility scores") for each arm will be calculated using the newly determined preference-based value set for the YBHRQL-Y (i.e. as determined in work package 5 of the overall programme grant), the existing value set for the HUI-3 and the CHU9D. They will be compared across the two arms, adjusting for baseline values.

8.3.4 Additional Analyses - Subgroup

Using interaction terms, we will explore whether a differential treatment effect is observed according to the following baseline factors: sex (male vs female), school age group (primary vs secondary), school setting (boarding during the week vs main-stream), congenital severe-to-profound deafness (yes vs no), whether participants are in criteria for iPads or Head Mounted Displays, vocabulary delay (no delay moderate delay vs severe delay), hearing age (used as a continuous outcome defined as the difference between chronological age and age at first implant), socio-economic status, parental education (degree and higher degree, A level and HMD/HMC, and all other), family's main language spoken at home (English or other), cochlear-implant usage (daily average at baseline) and loudness imbalance between ears.

8.3.5 Additional Analyses - Adjusted

Where appropriate, analyses will additionally adjust for other baseline factors. Further details on these analyses will be added to the SAP.

8.3.6 Additional Analyses - QoL instrument measurement properties

We will examine associations between the YBHRQL-Y, the VFS and speech in noise measurements at all measurement timepoints to assess the construct validity of the YBHRQL-Y in measuring the intended quality of life construct (listening effort, speech in noise, relative localisation and reaction time (also listening effort)). We will similarly explore differences in the sensitivity to change of the YHBRQL-Y over time compared to the HUI-3 and the CHU-9D.

8.3.7 Analysis Population and Missing Data

Analysis will be conducted on all patients randomised to either the BEARS or usual care intervention groups. Primary outcome analysis will follow the intention-to-treat (ITT) principle where all randomised participants are analysed in their allocated group, whether or not they receive their randomised intervention plan.

We will conduct sensitivity analyses to examine the potential influence of missing data under different pattern assumptions using joint models.

9 Economic Evaluations

9.1 Health Economic Analysis Plan

The health economic analysis of BEARS will be a within trial cost-effectiveness analysis. A detailed health economic analysis plan will be drafted alongside the statistical analysis plan described above and will be included as a section with the Statistical Analysis Plan (SAP) as the two analyses will complement each other and have certain overlaps in terms of data and analysis.

9.2 Within-trial analysis

9.2.1 Outcomes

9.2.1.1 Resource Use

Resource use will be collected using three modified CSRIs at baseline, 3and 12 months:

- Parents' CSRI (primary and community health care service use, urgent or emergency care and hospital admissions and time off school).
- Implant Centres CSRI (professionals in the implant centre including rehabilitationist, audiologist, clinical psychologist, surgical team, and others)
- Local education providers CSRI (teachers of the deaf, multi-sensory impairment teacher, special educational needs co-ordinator, other communication or teaching assistants, speech and language therapist, other professionals provided by the school). The Local education providers will be sent a copy of the CSRI by a member of the participant's clinical implant centre research team via post or email, which they will complete and return. This will then be uploaded onto OpenClinica by a delegated member of the clinical implant centre research team.

Baseline resource use (covering the 3 months before baseline) will be collected to allow costs to be adjusted for baseline values.

At 12 months, Parents' CSRI will ask for the preceding 3 months of data (covering the period from 9 to 12 months post baseline). The IC and Local education providers will be asked to provide the preceding 9 months of resource use information at 12-month timepoint.

We will report total resource use in each arm as well as according to suitable categories from the CSRI.

Resource use in the BEARS arm will also incorporate the cost of the intervention and other NHS, PSS and local education provider costs associated with using the intervention.

9.2.1.2 Health related Quality of life

Health related Quality of Life will be collected using three patient reported outcome measures:

- Self-complete version of the newly validated teenage version of the YBHRQL, the YBHRQL-Y (primary cost-utility analysis)
- The proxy completed version of the generic 15-question version of the HUI-3 (secondary cost-utility analysis)
- The self-complete version of the child specific CHU9D (secondary cost-utility analysis)

We will report mean utility values per participant by timepoint and arm for each measure.

9.2.3 Cost Data

9.2.3.1 Cost of the BEARS intervention

The cost of the intervention will be calculated using information on development costs from the group that developed the intervention, and any ongoing costs e.g. maintenance, and this will be included in the costs for the intervention arm. We will report a mean cost per participant of delivering the intervention, for the BEARS arm only. Costs incurred in the form of time spent by health care providers and other professionals in providing support to users of BEARS in using BEARS will also be included here.

9.2.3.2 Cost of Healthcare resource use

Unit costs will come from routine published sources or mean salaries where applicable. We will report total healthcare resource use costs as well as by categories from the CSRI, by arm for each time point.

Mean incremental cost and 95% confidence intervals between BEARS and usual care will be reported, using bootstrapped results adjusting for baseline values.

9.2.4 QALYs

For each of the three measures, QALYs will be calculated as area under the curve using utility scores at the different timepoints. We will report mean unadjusted QALYs by arm. Bootstrapped Mean incremental QALYs and 95% confidence intervals between BEARS and usual care will be reported adjusting for baseline utility values.

9.2.5 Analysis results

9.2.5.1 Primary analysis

We will report the incremental cost-effectiveness ratio (ICER) between BEARS and usual care from an NHS, PSS and local education provider perspective using QALYs calculated using the self-completed YBHRQL-Y. To represent the uncertainty around the cost-effectiveness results, we will report bootstrapped results on a cost-effectiveness plane (CEP) and a cost-effectiveness acceptability curve (CEAC) showing the probability of BEARS being cost-effective compared to usual care at different threshold values.

9.2.5.2 Missing data

Missing data will be dealt with as in the main statistical analysis, with the primary analysis being an intention to treat analysis and secondary analyses taking into account assumptions about missingness patterns and multiple imputation.

9.2.5.3 Sensitivity analysis

We will conduct one- and two-way sensitivity analyses for any assumptions made.

9.2.5.4 Secondary analyses

The analysis outlined in the primary analysis section will be repeated using QALYs calculated using the proxy HUI-3 and the self-complete CHU-9D measures. Further secondary analyses will incorporate costs outside health and education, namely time off school and out-of-pocket costs.

9.2.6 Additional analyses

Responses collected via the newly validated self-complete version of the YBHRQL-Y questionnaire will be compared against the proxy HUI and self-complete CHU-9D to provide further information on how

this new measure, previously validated in the preparatory work in Work Package 5 of this programme grant, perform within the larger trial context.

10 Regulatory & Ethical Issues

10.1 Compliance

10.1.1 Regulatory Compliance

The trial will be conducted in compliance with the approved protocol, the Declaration of Helsinki 1996, the principles of GCP as laid down by the ICH topic E6 (R2), General Data Protection Regulation and the UK Data Protection Act 2018 and the UK Policy Framework for Health and Social Care Research.

10.1.2 Site Compliance

Agreements that include detailed roles and responsibilities will be in place between participating sites, CCTU and the Sponsor.

Participating sites will inform CCTU as soon as they are aware of a possible serious breach of compliance, so that CCTU can fulfil its requirement to report the breach to the Sponsor (see section 7.9).

10.1.3 Data Collection & Retention

Clinical notes and administrative documentation should be kept in a secure location (for example, locked filing cabinets in a room with restricted access) During this period, all data should be accessible, with suitable notice, to the competent authorities, the Sponsor, and other relevant parties in accordance with the applicable regulations. The data may be subject to an audit by the competent authorities. Medical files of trial participants should be retained in accordance with the maximum period of time permitted by the hospital, institution or private practice.

Also see section 7.8 on data archiving requirements.

10.2 Ethical Approvals

10.2.1 Ethical Considerations

The following ethical considerations, problems and/or dilemmas relating to the trial should be considered:

- Additional hospital visits required as part of the trial. Although the majority of the visits are completed online, the participant and their parent/legal representative will have to visit their implant clinic for two additional visits to their standard care. This might involve taking time off work or out of school.
- There are not any invasive tests as part of the BEARS trial. The participants though are expected to complete two speech tests and six questionnaires, which are all additional to their usual care
- BEARS is a randomised controlled trial. Participants are therefore unable to choose what intervention arm they receive. This could cause for disappointment (from the participant and/or their parent/legal representative), if participants are not randomised to receive the BEARS intervention. Those randomised to the usual care arm will be given the opportunity to

trial the BEARS training package. This will be arranged by the BEARS CI and those delegated the responsibility.

- Participants that used the BEARS training package might want to continue to use it once they have completed all trial visits, especially if they have noticed an improvement in speech, relative localisation, listening effort, vocabulary, hearing and quality of life. This will not be possible and therefore could cause participants to become unengaged with the trial.
- Participants that used the BEARS training package and have noticed an overall improvement in speech and listening outcomes might notice a decline in these improvements once no longer using the training package. This could cause the participant and/or their parent/legal representative to feel disheartened in regard to their CI experience.
- Participants will receive a £10 gift voucher upon completing the Baseline appointment and £20 upon completion of the 3 and 12-month follow-up appointments. This will total £50.They will also be reimbursed for travel expenses for each in-person clinic visit. Participants and/or their legal representatives may want to take part in the trial due to the financial gain, rather than because they are a suitable candidate. Please note that for participants recruited from the 1st November 2025 this will be at total of £30.

These issues are fully explored with mitigation strategies provided for in the CCTU BEARS Risk Assessment documentation.

10.2.2 Ethics Committee Approval

The BEARS clinical trial documents will be submitted to the following REC for approval: Yorkshire & The Humber - Sheffield Research Ethics Committee

10.3 Competent Authority Approvals

This is not a Clinical Trial of an Investigational Medicinal Product (IMP) as defined by the EU Directive 2001/20/EC. In addition, the UK Competent Authority, the MHRA, does not consider the BEARS software training package to be a medical device. Therefore, a Clinical Trial Authorisation (CTA) is not required in the UK.

The progress of the trial and safety issues will be reported via the Annual Progress Report form to the REC in accordance with HRA's requirements.

Safety reports, specifically expedited reporting for 'related and unexpected' SAEs, will be submitted to REC in accordance with the HRA's requirements.

10.4 Other Approvals

Following main REC approval and Health Research Authority (in England) approvals and before initiation of the trial at each clinical site, the local information pack will be submitted to each Trust's Research and Development (R&D) office by UCL CCTU. The local information pack will contain the protocol, all informed consent forms, and information materials to be given to the prospective participant, the Clinial Trial Site Agreement, the Organisation Information Document (OID), and the validated Schedule of Events Cost Attribution Template (SoECAT). In Wales, Scotland and Northern Ireland, the R&D office will be asked to give approval. In England, the R&D office will be asked to confirm capacity and capability. A copy of the local permissions (or other relevant approval as above) and of the PIS and ICF on local headed paper must be forwarded to the CCTU before participants are

entered. The CCTU will then issue an email to the site giving the greenlight for the site to start seeing participants as part of the trial.

Any amendments will be submitted to the local R&D offices, who will need to give approval/confirm capacity and capability before implementing them at site.

10.5 Trial Closure

Trial closure is defined as the date when all data have been received and cleaned (all data queries resolved at all sites) and the database locked for final analysis. For BEARS this will be after all queries have been resolved following the last participant completing their last follow-up visit measures.

The REC/HRA will be notified within 90 days of trial completion. Within one year of the end of the trial, the CCTU will submit a final trial report with the results of the trial, including any publications/abstracts of the trial, to the HRA. In the case that trial is ended prematurely, the CCTU will notify the HRA within 15 days, including the reasons for the premature termination. All reports will be reviewed and approved by the CI and Sponsor.

At a site level, a site/implant centre may only be deemed 'closed' once all trial-related activities have been reconciled and/or complete, all outstanding data queries resolved; all necessary trial documentation has been provided to the participating sites for inclusion in the ISF; following the Trial Manager or delegate at the CCTU performing a close-out visit or teleconference, as appropriate. The close-out process will be documented accordingly. Any outstanding actions resulting from the close-out visit will need to be completed and documented within an agreed timeframe between the participating site and CCTU. The responsibility for completing any outstanding actions will lie both with the local research staff and Trial Manager or delegate. A letter confirming that close out is complete has been sent to the site PI from the CCTU.

11 Indemnity

This study is sponsored by Guy's and St Thomas' NHS Foundation Trust (GSTT) and indemnity is provided through NHS indemnity for clinical negligence. GSTT is a member of the clinical Negligence Scheme for Trusts (CNST). In the case of negligent harm, health care professionals undertaking clinical trials or studies on volunteers, whether healthy or patients, in the course of their NHS employment are covered by NHS Indemnity. In the case of non-negligent harm legal liability does not arise where a person is harmed but no one has acted negligently. In exceptional circumstances NHS bodies may consider whether an ex-gratia payment could be offered.

12 Finance

BEARS is fully funded by an NIHR Programme grant number NIHR201608. It is not expected that any further external funding will be sought.

13 Oversight & Trial Committees

Trial oversight is intended to preserve the integrity of the trial by independently verifying a variety of processes and prompting corrective action where necessary.

There are a number of committees involved with the oversight of the trial. These committees are detailed below.

13.1 Trial Management Group

A Trial Management Group (TMG) will be formed comprising the Chief Investigator, other lead investigators (clinical and non-clinical) and CCTU staff and PPI contributors. The TMG will be responsible for the design, coordination and strategic management of the trial. The membership, frequency of meetings, activity (including trial conduct and data review) and authority will be covered in the TMG Terms of Reference.

Note that the wider BEARS Programme also has a Programme Management Group (PMG) that has logistical responsibility for all Work Packages (1-8). The two groups will be conducted at the same meeting, but the emphasis will change as some of the work packages finish and the trial begins. Responsibility for the overall programme management and dissemination plan will not be devolved but the running of the trial and the process evaluation will be.

13.2 Programme Steering Committee

The Programme Steering Committee (PSC) is the independent group responsible for oversight of the Programme, including Work Package 6/RCT, and to safeguard the interests of trial participants. The PSC provides advice to the CI, CCTU, the funder and sponsor on all aspects of the trial through its independent Chair. The membership, frequency of meetings, activity (including trial conduct and data review) and authority will be covered in the PSC terms of reference.

13.3 Data Monitoring and Ethics Committee

The Data Monitoring and Ethics Committee (DMEC) is the only oversight body that has access to the full trial data set for the trial. The DMEC is responsible for safeguarding the interests of trial participants, as well as monitoring the accumulating data and making recommendations to the PSC on whether the trial should continue as planned. The membership, frequency of meetings, activity and authority will be covered in the DMEC Terms of Reference. The DMEC will consider data in accordance with the statistical analysis plan and will advise the PSC through its Chair.

13.4 Trial Sponsor

The role of the sponsor is to take on responsibility for securing the arrangements to initiate, manage and ensure there is sufficient funding to cover the duration of the study. GSTT is the trial sponsor and has delegated the duties as sponsor to CCTU via an amendment to the BEARS collaborator agreement. Details of the specific responsibilities that have been delegated are detailed in the BEARS: Roles and Responsibilities document.

14 Patient & Public Involvement

Patient and Public Involvement (PPI) in research is defined by INVOLVE (an advisory group established by the NIHR) as research being carried out 'with' or 'by' members of the public rather than 'to', 'about' or 'for' them. INVOLVE intends 'public' to include patients, potential patients, carers and other users of health and social care services, as well as people from organisations that represent people who use services. In some cases, this may include involvement of a trial's participants in guidance or oversight of a trial.

14.1 Potential Impact of PPI

There has been PPI involvement throughout the BEARS programme.

PPI groups have previously tested and given feedback on the BEARS training package software and hardware, the Speech-in-Noise tests being used as outcome measures and provided suggestions on dissemination and communication of the research outcomes of the BEARS trial. The PPI 'Writing Review' group provide input on the patient materials used as part of the clinical trial. Engagement with the patient population in BEARS will hopefully allow for successful recruitment and retention of participants.

PPI involvement will continue once the trial has opened to recruitment, by having a representative attend the monthly TMG meetings.

Each of the PPI groups will receive age-appropriate training from the Cambridge Biomedical Research Centre and will have continuous support from the PPI Coordinator throughout their involvement in the trial

14.2 Identifying PPI Contributors

The BEARS programme has identified and collaborated with PPI contributors prior to the RCT Work Package (WP6) commencing. Each Work Package has several PPI groups in order to discuss the BEARS training package hardware and software and the outcome assessments. The PPI groups include patients 8-24 (inclusive) years old with bilateral CIs, teachers of the deaf and clinicians.

15 Publication & Dissemination of Results

15.1 Publication Policy

15.1.1 Trial Results

The results of the trial will be disseminated regardless of the direction of effect. The publication of the results will comply with the BEARS specific publication policy (generated through BEARS Work Package 8 on Dissemination) and will include submission to open access journals.

A lay summary of the results will also be produced and uploaded on to the BEARS trial website. All participants will be able to view this document. This has been detailed in the patient information sheets.

A summary of results will be included online in the publicly accessible HRA website within 12 months of date of trial closure.

15.1.2 Authorship

As part of the BEARS Programme Work Package 8, the PMG will advise on the nature of publications. All publications shall include a list of investigators, and if there are named authors, these should include the Chief Scientific Investigator, Chief Investigator, and appropriate Co-Investigators, Trial Manager, and Statistician(s) involved in the trial (and/or data analysis). Named authors will be agreed by the Chief Investigator(s) and in line with the BEARS publication policy. If there are no named authors, then a writing committee will be identified.

15.1.3 Reproducible Research

The BEARS Trial Protocol and Statistical Analysis Plan will be published for public access.

Docusign Envelope ID: 5CA44ABD-F65C-8DE8-80E3-4572550DF9E0

16 Data Sharing

Data will be shared in accordance with this Protocol, the Data Protection Legislation and Funder (NIHR) terms, based on the following principles:

- No data should be released that would compromise an ongoing BEARS project.
- There must be a strong scientific or other legitimate rationale for the data to be used for the requested purpose of the BEARS Project.
- Data be made available on an open access basis for research purposes.
- Data exchange complies with the applicable Information Governance and Data Security Policies.

Data will be available for sharing for the purposes of the BEARS project between the project researchers as detailed in the WP6/RCT Data Sharing Agreement between the institutions hosting the BEARS researchers.

16.1 Post Project

Requests for access to Project Data for non-commercial future research will be considered, and approved in writing where appropriate, after formal application, subject to the Sponsor, PMG, PSC and NIHR's approval.

17 Sub Studies

The qualitative research (section 17.1) and PE research (section 17.2)

17.1 Qualitative Research

Study Design	Qualitative methodology
Aims	• To explore participants experiences of everyday listening as users of bilateral cochlear implants<br>• To contribute to understanding how the BEARS intervention may lead to perceived changes to the experience of everyday listening as users of bilateral cochlear implants
Data collection methods	In-depth interviews and surveys
Sample size	In-depth interviews: A minimum of 20 participants from each randomisation arm.<br>Surveys will be completed by all participants as part of the RCT.

17.1.1 Background/rationale

The full background to the study is given in section one. As outlined in section 1.8, the aim and objectives of the BEARS clinical trial are to determine whether the use of BEARS for a period of 3 months:

1. Improves spatial speech-in-noise perception
2. Improves localisation abilities
3. Improves music listening experiences
4. Reduces listening effort
5. Increases vocabulary age
6. Improves quality of life
7. Is cost effective
8. Improves perceived benefits of everyday listening.

17.1.2 Study design

Qualitative methodology will be used to specifically address trial objectives 3) and 8) to explore whether BEARS improves music listening experiences and improves the perceived benefits of everyday listening. Qualitative data will also complement the quantitative data collected to address objectives 1-2, 4 and 6.

17.1.3 Aims and objectives

The aim of the qualitative research is two-fold:

- To explore participants experiences of everyday listening as users of bilateral cochlear implants
- To contribute to understanding how the BEARS intervention may lead to perceived changes to the experience of everyday listening as users of bilateral cochlear implants

This data will contribute to and deepen our understanding of how the BEARS intervention or the Usual Care pathway impacts on participants' everyday listening. This will include whether the breadth/range

of quantitative outcomes used in the trial reflect what is important to participants, and identify reasons for any potential variation/differences in outcomes between individuals or sites [71, 72].

17.1.4 Research Questions

1. What is the experience of everyday listening for young people using bilateral cochlear implants? To include:
    a. Listening to speech in noise
    b. Localising sound sources
    c. Listening effort
2. What is the experience of listening to music for older children/teenagers using bilateral cochlear implants?
3. How do older children/teenagers using bilateral cochlear implants perceive changes to their everyday listening during and after their participation in the BEARS trial?
4. How do older children/teenagers using bilateral cochlear implants perceive changes to their listening to music during and after participation in the BEARS trial?
5. How do older children/teenagers account for perceived changes after their participation in the BEARS trial?

17.1.5 Theoretical framework

Deaf or hard of hearing (DHH) children experience speech and language difficulties even when early diagnosed and implanted, although there is diversity of experience depending on severity of hearing loss, age at implant and other factors [73]. DHH children and young people require ongoing training to master good functional communication as well as the more academic language skills necessary for literacy [74]. Good spoken language skills result in improved social relationships, well-being and educational outcomes [75, 76]. BEARS aims to improve all these skills and many measures are being made to quantify any changes; however, DHH children are a heterogeneous group with many individual characteristics and contextual factors. In order to better understand how these differences impact on measured changes (or no changes), and as has been shown to be valuable in other studies [77], in this qualitative study, we will gather the views and experiences of (a subset of) the participants.

17.1.6 Participants and setting

In-depth interviews will be conducted with a sample of participants from both arms at baseline and again at 3 months. Data will be collected across all sites participating in the study.

All participants in both BEARS and Usual Care arms will be given the opportunity to provide brief, key qualitative data via an online survey at baseline, 3, and 12 months. Data will be collected across all sites participating in the study.

17.1.7 Sampling: in-depth interviews

Purposive sampling [78] will be used to gain perspectives from a range of participants according to their age, sex and site.

Interviews will be conducted with a minimum of 20 participants in each of the BEARS and the Usual Care arms. For each of BEARS and Usual Care arms, we will aim for at least 10 participants in the younger group (age 8-11), and 10 in the older group (age 12-16). In each age group, we will aim for a mix of boys and girls in each grouping. For the interview conducted at baseline, both the participant

and the researcher conducting the interview will be blind to which arm the participant has been randomised to. The final sample size will be determined by reaching data saturation, i.e. the point at which no new themes are identified in the data.

17.1.8 Sample identification and recruitment: in-depth interviews

The CCTU team members will identify potential participants from the trial database who have provided electronic consent/assent to take part in an interview, according to the sampling criteria (i.e. age, sex, site, trial arm). CCTU will inform the qualitative research team of potential participants who meet the sampling criteria by their clinical identifier. Members of the qualitative research team will contact the participants' respective site to gather information on the participants' name/contact details. They will then send the participant/their parent/legal representative the appropriate PIS. The qualitative research team members will then contact the child/parent to discuss the interview thus providing an opportunity for the child/parent to ask any questions, to discuss the child's preferred method of communication and to arrange a convenient time for the interview to take place. In addition to providing electronic consent/assent to be interviewed, verbal consent/assent will be gained from participants prior to the start of each interview.

17.1.9 Data collection procedures

17.1.9.1 In-depth interviews

In-depth, semi-structured individual interviews will be carried out by the BEARS qualitative researchers. Interviews will be conducted at baseline and at 3 months and are expected to last 30-60 minutes. Interviews will take place virtually/online using Zoom and will be video recorded with consent/assent. As DHH children can use both verbal and non-verbal communication (e.g. using gestures and/or sign language vocabulary to augment spoken words), a video recording of the interview is needed to ensure we are able to accurately record the interaction between the participant and interviewer [79].

The rationale for using Zoom rather than another videoconferencing platform such as MS Teams is as follows:

- Children and families will be more familiar with Zoom, and find it more accessible and easier to use than MS Teams
- MS Teams is mostly used within workplaces, therefore it is likely to be less accessible for children/families to use at home, especially if they only have access to a smartphone, not another device
- The group we are interviewing are a vulnerable population; we need to ensure that participation in the study is as straightforward as possible and aim to reduce the burden where we can
- One of the NIHRs (study funder) key priorities is equality, diversity and inclusion. They emphasise the need to improve inclusion of under-served groups (e.g. children, deaf people) in research. Using Zoom will support this by increasing opportunities for a more diverse group of children to participate in interviews
- UCL (institution hosting qualitative research team) and study sponsors have provided agreement that Zoom can be used as we will utilise safety features within Zoom, such as passwords and the waiting room, to ensure a closed, secure environment.

As we will be using the audio transcription feature in Zoom to automatically transcribe the interview we will need to record and save to Zoom. Zoom settings will be enabled so only the interviewer can access the recordings and transcription. The Zoom audio transcript will provide us with an initial transcript; however, these transcripts will need to be significantly expanded to ensure an accurate, verbatim transcription as deaf children/young people's speech may not be fully intelligible. This further transcription will be completed by a UCL approved, GDPR compliant transcription service and checked by members a professional transcription service and then further enhanced by members of the research team who are experienced in transcribing deaf speech and non-verbal communication.

Interviews will be guided by a topic guide developed with PPI input. Flexibility and creativity will be key when conducting the interviews to facilitate participants with differing levels of communication and understanding to share their views. Included will be the options of: writing the question in the chat function available in Zoom; using pictures/photographs to support participants understanding of concepts; using British Sign Language (BSL) interpreters where needed should any participants have BSL as their primary language and prefer to provide their responses in BSL; and sending the participant a copy of the topic guide in advance of the interview. If participants prefer to have their parent present during the interview this will be accommodated.

Participants will receive a £10 gift voucher upon completing each of the qualitative interviews.

17.1.9.2 Qualitative online survey

All participants in both arms will be asked to respond to an open-ended question at baseline, 3, and 12 months. These questions will be incorporated into the package of trial questionnaires/quantitative outcome measures administered via the online survey platform used as part of the whole trial. Questions will explore participants' experiences of listening with their cochlear implants in everyday contexts, their perceptions of how this experience might change during the trial and whether they would like further training to help their listening skills. Qualitative data will be collected from participants' free-text responses to each question. If a child needs help with writing a response to these questions, a parent/carer can assist them. Questions have been developed with PPI input.

17.1.10 Data analysis

17.1.10.1 Interview data

Transcriptions will be imported into NVivo12 software to facilitate data handling, organisation and coding. Data will be analysed thematically using Framework analysis. This matrix-based analytic method is flexible and systematic and facilitates rigorous and transparent data management. This method involves five distinct, but highly connected stages which enable data to be analysed iteratively (1) data familiarisation; (2) identifying a thematic framework; (3) indexing all study data against the framework; (4) charting to summarize the indexed data; and (5) mapping and interpretation of patterns found within the charts [80]. The systematic movement through the five stages provides an explicit audit trail [81]. It is particularly suited to analysis by a team of researchers, and it allows easy access to synthesised data so that it can be continually revisited. The development of an analytical framework enables highlighting both key issues related to the research questions (e.g. understanding speech in noise, localising sound sources and listening to music) and emergent data-driven themes. All themes will be developed and revised in an iterative manner as patterns within the data become more apparent. Emerging themes will be discussed in regular meetings with the research team to

reach consensus on the major themes. The analysis will be complemented by discussions with our PPI group.

17.1.10.2 Qualitative survey data

Framework analysis will also be used to analyse the text-based qualitative data collected via the online surveys.

17.1.10.3 Mixed methods integration of qualitative and quantitative data

Quantitative and qualitative data will be integrated during the analysis and the interpretation stages. Both types of data will be available for the subset of cases (individual children participating in the interviews). At the analysis stage, we will consider integrating data by comparing a child's responses and scores on the questionnaires, surveys and speech tests with their interview transcript to identify convergence, complementarity or contradictions [82]. In addition, we will integrate qualitative and quantitative data at the interpretation stage; for example, using a joint display technique such as the Pillar Integration Process [83] to enable us to contextualise and explain the reasons for the trial findings.

17.1.11 Qualitative data management

17.1.11. Qualitative interview data

Following each interview, the interviewer will download the audio and video recording and audio transcription from Zoom and transfer them directly into a private channel on Microsoft Teams/SharePoint only accessible to members of the qualitative research team. All recordings and transcriptions on Zoom will be deleted. Clinical trial identifiers will be used to pseudonymize the digital files in SharePoint. Pseudonymized audio recordings will be shared with the approved transcription service for professional transcription via the transcription service's secure online platform. As necessary, the research team will enhance the transcriptions with reference to the video recordings. Any identifying information will be removed from the transcriptions, e.g. any names used by interviewees will be pseudonymized. Transcriptions will be stored on SharePoint and imported into NVivo software for analysis within SharePoint. Participants will not be named in the transcriptions or in the analysis or in any subsequent reports about the research. When publishing results, care will be taken not to report information that will enable participants to be identified, for example in relation to rare conditions or geographical locations.

Audio/video recordings and transcripts will be stored on Teams/SharePoint for the duration of the project. Once the project has finished, audio/video recordings and transcripts will be downloaded from SharePoint and saved on an external encrypted hard drive for the archiving retention period. Any data on SharePoint will be deleted.

17.1.11.2 Qualitative text-based survey data

Data will be stored on the private channel on Microsoft Teams/SharePoint only accessible to members of the qualitative research team. Data will be pseudonymized. Clinical trial identifier will be used to pseudonymize data and any identifying information will be removed. Data will be imported into NVivo software for analysis within SharePoint. Data will be stored on Teams/SharePoint for the duration of the project. Once the project has finished, this data will be downloaded from SharePoint and saved on an external encrypted hard drive for the archiving retention period. Any data on SharePoint will be deleted.

17.1.12 Ethical considerations

Several steps will be taken in order to safeguard participants taking part in interviews. We will pay attention to obtaining consent/assent, using a range of chronologically and developmentally age-appropriate study information materials. We will give thought to the various ways in which children with bilateral cochlear implants may express their views, including providing sign language support where necessary and respecting any wish to withdraw from the interview. Should any child become distressed during an interview, they will be the given the option to have a break, move onto the next question and/or stop the interview. The interviewer will signpost participants who experience any distress during interviews to appropriate sources of support. Participants will be advised what they share will be strictly confidential. The only reason we would break confidentiality and share any information is if we felt concerned that someone was in danger of harm. Participants will be informed if we deem it necessary to break confidentiality.

17.1.13 Patient and Public Involvement

PPI advisory groups consisting of DHH children/young people, and parents who have a child with an implant, have been established to advise on the design and delivery of the study. PPI contributors have been involved in different aspects of the study including advising on the interview topic guides and open text/qualitative survey questions, and will continue to have input to the study, including qualitative data analysis and dissemination.

17.1.14 Reporting and dissemination

Results from the study will be disseminated widely as detailed earlier in the protocol. The qualitative research will be disseminated through international peer-reviewed journals and relevant conferences, reaching different professional, lay and academic audiences. This will include a: 1) qualitative paper reporting on the everyday listening experiences of children with bilateral cochlear implants, 2) mixed methods paper that contextualises and explains the reason for the trial findings.

17.2 Process Evaluation research Protocol

17.2.1 Background

Process evaluation plays an important role in informing policy and practice following the evaluation of an intervention [84]. This is typically achieved by investigating aspects of implementation, change mechanisms and how context influences change mechanisms and intervention impact. Contextual factors may include individual characteristics, family, social network or organisations, such as a school or healthcare service. For example, a process evaluation could investigate whether the intervention worked better for older or younger people or in particular types of clinical settings.

Process evaluation can help determine why an intervention works or fails or has unanticipated consequences and how it can be optimised. The results can inform an implementation strategy and provide valuable information for decision-makers who seek to implement the intervention across a range of settings [58].

Based on the logic model outlined in section 1.3, the process evaluation will systematically analyse intervention delivery, fidelity, dose and reach as well as moderators, mediators and confounding variables that might impact the intervention.

The process evaluation will be complementary to the clinical trial and will be an independent parallel process to reduce potential biases in analysis of outcome data. The process evaluation data will not be collected by those involved in designing or implementing the intervention.

17.2.2 Aims

1. To determine if the RCT was delivered as planned (trial implementation)
2. To analyse how context influences intervention impact
3. To explore the participant experience of how BEARS produces change

17.2.3 Objectives

1. Determine the fidelity of the trial
2. Determine the dose of the trial
3. Determine the reach of the trial
4. Determine the quality of the implementation
5. Explore the subjective influence of change mechanism 'Enhancement via multisensory integration' on learning outcomes.
6. Explore the subjective experience of change mechanism 'Generalisation via multimodal stimuli and cognitive engagement'
7. Identify contextual factors that might be associated with variations in outcome
8. Identify factors that support the implementation of the intervention into general routine practice
9. Identify enablers and barriers to the deployment of the intervention

17.2.4 Process Evaluation Outputs

1. Description of the quality of trial delivery
2. An analysis of how context influences impact
3. Qualitative analysis of the user experience of using BEARS and how BEARS leads to change
4. Qualitative analysis of the clinician experience of implementing the trial
5. Recommendations for implementation, deployment and scaling up

17.2.5 Study Design

The process evaluation will adopt a mixed methods approach to collect and analyse quantitative and qualitative data. A mixed methods triangulation convergence design will be used [85]. Quantitative and qualitative data will be given equal emphasis and will provide different but complementary data to best answer the research questions. This combines the differing strengths and non-overlapping weaknesses of quantitative methods (large sample size, trends, generalization) with those of qualitative methods (small sample size, details, in depth). Concurrent but separate quantitative and qualitative data collection and analysis will be followed by integrated data analysis and dissemination of findings.

17.2.6 Research Aims

Table 4: Research aims and questions for Process Evaluation

Objective	Question	Qualitative question	Quantitative question
1	Determine the fidelity of the trial	X	X
2	Determine the dose of the trial		X
3	Determine the reach of the trial		X
4	Determine the quality of the implementation	X	X

5	Explore the subjective influence of change mechanism 'Enhancement via multisensory integration' on learning outcomes.		X
6	Explore the subjective experience of change mechanism 'Generalisation via multimodal stimuli and cognitive engagement'	X	
7	Identify contextual factors that might be associated with variations in outcome	X	X
8	Identify factors that support the implementation of the intervention into general routine practice	X	X
9	Identify enablers and barriers to the deployment of the intervention	X	X
Additional exploratory analysis	To what extent is the primary outcome and BEARS usage associated with differential levels of change in participant behaviour?		X

17.2.7 Theoretical framework

Due to variability across age and how children of different ages interact and engage with technology to become autonomous users [86], the process evaluation leans on theoretical models that consider individual differences for effective and continued use of technology [87, 88]. The importance of agency (or autonomy) being a predictor of future technology use is important, and teenagers with lower levels of agency are likely to need a higher level of support to encourage autonomy to ensure continued use and engagement.

To understand implementation within the health service data collection and analyses are informed by Normalisation Process Theory (NPT) which is directly applicable to the implementation and embedding of a new complex intervention in a healthcare setting [89, 90]. The four key constructs of NPT are:

1. ***Coherence***: Sense-making work to operationalise an intervention through understanding the intervention, its value and benefits
2. ***Cognitive participation***: Relationship work to build a community of practice around the intervention
3. ***Collective action***: Enacting work to operationalise a set of practices to deliver the intervention
4. ***Reflexive monitoring***: Appraisal work to assess and understand ways that the new practices affect clinicians delivering them and others around them, for example how useful the intervention is and how clinical practices might be modified.

17.2.8 Sample identification and recruitment

The Process Evaluation quantitative questionnaires will be asked to all participants as part of the questionnaires completed in the RCT trial. Participants will consent to completing these questionnaires as part of the E-consent procedure detailed in section 3.5.

Clinicians, who are seeing the participants as part of the BEARS trial at each site will consent to complete the NoMAD questionnaire. Consent will be taken electronically via the Qualtrics GDPR compliant platform by members of the Process Evaluation team. The Process Evaluation quantitative team will send the electronic PIS and consent form to each clinician's email address via the Qualtrics platform. The clinicians will complete the consent form providing an electronic signature, with the clinician permitted to access and respond to the questionnaire on Qualtrics only once they have consented. Once the clinician signs the consent form, a confirmation will be generated for the

researcher, allowing them to countersign the consent form. A fully executed PDF will then be sent to all parties.

Participants will consent to take part in the Process Evaluation qualitative interviews as per the process documented in section 17.1.8 of the protocol.

17.2.9 Quantitative Data Collection and Analysis

17.2.9.1. Overview of Data Collection

Participant inclusion and exclusion criteria are provided in section 3.1. For the purpose of the Process Evaluation, all participants in the BEARS intervention (*N* =136) as well as the usual care pathway (*N* =136) will be included in the quantitative data collection. Exceptions to this are measures that specifically relate to usage of the BEARS intervention, which will only be completed by participants and their caregivers in the intervention arm.

Quantitative data will be collected using online questionnaires completed by participants, caregivers, and clinicians. Measures will gauge the quality of information and training delivered, trial implementation and potential moderating and mediating factors. Quantitative usage data from participants will also be collected automatically via the BEARS software to inform the fidelity analysis.

17.2.9.2 Data Collection from Clinicians, Caregivers and Participants

Table 5 outlines the measures required for the quantitative Process Evaluation. Further details of these measures will be available in the BEARS PE working document.

Table 5: Quantitative Measures for Process Evaluation

	**Assessment**
1	Participant implant data (reach)
2	Implant centre data (enablers and barriers)
3	Sociodemographic information (reach)
4	CRF (fidelity and quality of implementation)
5	Helpdesk data logging (fidelity, enablers and barriers)
6	Data logging within BEARS app
7	Normalisation Measure Development (NoMAD) questionnaire [91]
8	Caregiver questionnaire
9	Participant questionnaire
10	BEARS in-app questions
11	Technological engagement
12	Home environment
13	Strengths and Difficulties Questionnaire (SDQ; [92])
14	User Experience of BEARS apps (developed for BEARS during PPI workshop (February 2022) and based on [93, 94]
15	User Experience of Headset

Note. Entries shaded in grey are collected as part of WP6.

17.2.9.2.1 Clinicians

Complementing the qualitative data collection, the quantitative process evaluation will adopt the NPT approach, using the NoMAD questionnaire [91]. The NoMAD questionnaire will be used to determine the views of clinicians towards the intervention and their willingness to incorporate it into their

routine practice. Clinicians will be asked to complete the NoMAD questionnaire following their initial training at baseline and again following their final patient appointment, to capture change.

17.2.9.2.2 Caregivers

Caregivers will be asked to report on their child's prior technological access and engagement at baseline and behaviour (SDQ;[92]) at baseline, 3 and 12 months. This will allow an analysis of the trial outcomes in relation to parent-reported behavioural strengths and weaknesses. Caregivers will also be asked to complete a questionnaire to detail their experience of the intervention delivery and incorporation of the BEARS into their routines as well as the convenience and management of the trial. This questionnaire will allow caregiver views to be assessed and fidelity to be monitored, as well as providing an understanding of enablers and barriers to engagement.

17.2.9.2.3 Participants

For participants in the BEARS arm the following measures will apply. Participants will be asked to complete a questionnaire to detail their knowledge of the intervention and what is required and their experience of the intervention delivery following the initial clinician session. Participants will also be asked about their expectations of BEARS in terms of helping them individually. Throughout the trial, participants will be asked questions about their motivation to use, confidence using, and enjoyment of the BEARS app. These questions will be delivered through the participant's BEARS device, with participants automatically prompted to respond once weekly. Following the end of the intervention period, participants will be asked to complete a questionnaire to indicate where they used BEARS (home environment), their user experience of BEARS and if applicable, the VR headset. Together, these measures will allow an examination of how context (user experience, confidence and motivation) may relate to the primary trial outcomes. Fidelity, in terms of participant adherence and dose received, will be determined using automated data logging from the software used to deliver BEARS using VR technology. This will include the number of times used in a week, the duration of sessions, type of training and level achieved. BEARS automatically logs progression and frequency of use, and the player's performance scores, in an anonymised format.

17.2.10 Data Collection and Data Transfer

Data will be collected in online questionnaires delivered through the OpenClinica electronic database, with participants, caregivers and clinicians prompted to respond at the timepoints stated in section 5.2.

Data logging from the BEARS software for participants in the intervention arm will be downloaded when the devices are returned at the end of the intervention period. MATLAB scripts will be used to process the data, which will be manually inserted into the OpenClinica electronic database.

17.2.11 Data Analysis Plans

The process evaluation analysis of BEARS will address each of the quantitative research questions outlined in table 4. We will report descriptive statistics for fidelity, dose, reach, quality of implementation, and the factors supporting implementation and deployment. A regressive framework will be used to predict the primary outcome from contextual factors and factors supporting implementation and deployment. The relationship between BEARS usage, the primary outcome and parent-reported participant behaviour will also be examined in an exploratory regressive framework. Missing primary outcome data will be dealt with as in the main statistical analysis. Further details of the data analysis plans will be available in the BEARS PE working document (to be created).

17.2.12 Qualitative Data Collection

17.2.12.1 Case study sites

We will identify case study sites from which to draw clinician participants and, where possible, intervention arm and caregiver participants. Intervention arm and caregiver participants may also be drawn from non-case study sites where necessary to achieve sufficient variation in the participant/caregiver sample. Sites will be purposively sampled to obtain maximum variation of:

- Healthcare system
- Type of provider centre – NHS and non-NHS providers
- Size of centre (number of patients and clinicians)
- Geographical location
- Prior site research experience
- Trial delivery – whether trial testing is by local clinicians or the research team

17.2.12.2 Participant and caregiver semi-structured interviews

Online (Zoom) semi-structured interviews will be conducted with a sample of intervention and usual care participants and their caregivers. Interviews may be jointly with child and carer-giver or separately if preferred. Participants will be purposively sampled to obtain maximum variation of:

- Age (primary school children / children in year 7 transition / secondary school children)
- Sex
- Device used – headset or tablet (intervention arm participants only)
- Site

Final sample size will be determined by reaching data saturation, i.e. the point at which no new themes are identified in the data. We anticipate a minimum of 20 interviews with intervention participants.

Interviews will be 30-60 minutes and conducted post-intervention. Topics guides will be provided in the PE working document. The language used in questions will be informed by the learning from Qualitative Pre-Test Interviews conducted in WP5 to ensure that it is meaningful for participants. We will take a flexible and creative approach to conducting interviews to enable participants with differing levels of communication to share their views, for example using pictures or writing in the chat function. British Sign Language (BSL) interpreters will be used where needed. A copy of the topic guide will be available in advance of the interview. Participants may have a parent present during the interview if they wish.

Deaf children can use both verbal and non-verbal communication and so interviews will be video recorded [79].

17.2.12.3 Participant qualitative online survey

All intervention arm participants will be asked to provide brief free-text responses to six open-ended questions (provided in the separate PE working document) at post-intervention. These will be collected as part of the package of trial questionnaires/quantitative outcome measures administered via the online survey platform.

Data will be stored on the private channel on Microsoft Teams/SharePoint only accessible to members of the qualitative research team. Data will be pseudonymized. Clinical trial identifiers will be used to

pseudonymize data and any identifying information will be removed. Data will be imported into NVivo software for analysis within SharePoint.

17.2.12.4 Clinician semi-structured focus groups/interviews

At each case study site, we will recruit clinicians who are delivering the trial in both the intervention and control arms. Where possible we will recruit an audiologist, rehabilitationist and community rehabilitationist at each site. Data will be collected in online focus groups or interviews depending on clinician availability. The topic guide (provided in the separate PE working document) will address trial delivery and factors relevant to implementation. Interviews are expected to last between 30-60 minutes and focus groups 1-2 hours. All will be audio-recorded and transcribed verbatim.

17.2.13 PE Qualitative Data Management

This will follow the same process, detailed in section 17.1.11.

17.2.14 Qualitative Data Analysis

Interview/focus group transcriptions and qualitative survey data will be imported into NVivo10 software to facilitate data handling, organisation and coding.

For participant and caregiver interviews a coding frame informed by the Technology Adoption Model [95] and the Technology Integration Model [88] will drive deductive coding, complemented by inductive coding to capture emergent data-driven themes. For clinician interviews a coding frame informed by Normalisation Process Theory will drive deductive coding complemented by inductive coding to capture emergent data-driven themes.

Data will be analysed thematically using Framework analysis [96]. Scripts will be read, re-read and coded by two researchers. Codes will be grouped into categories and a working analytical framework developed which will be applied to all transcripts. Data will be summarised by category in framework matrices on which the final interpretation will be based.

To explore research question 7.1 (What contextual factors are associated with differential levels of change in the primary outcome?) BEARS users will be stratified into low to high benefit within the Framework matrix and compared and contrasted on contextual factors such as previous technology engagement, home environment, parental support, confidence and motivation to use BEARS, time availability and commitment, experience of BEARS and device used, use and experience of BEARS tech support.

Themes will be developed and revised in an iterative manner as patterns within the data become more apparent. Emerging themes will be discussed in regular meetings with the research team and PPI groups to reach consensus on the major themes.

17.2.15 Mixed Methods Integrated Interpretation

Quantitative and qualitative data provide different but complementary data to address the research questions. Following independent data analysis, quantitative and qualitative findings will be integrated using the Triangulation design - Convergence model [85]. Quantitative and qualitative findings related to each research question will be compared and contrasted to validate, confirm or corroborate the findings in order to develop valid and well-substantiated conclusions.

In the intervention and usual care arms we will explore within and between group phenomena (e.g., younger children vs teenagers; boys vs girls; baseline scores and degree of change on trial outcomes).

17.2.16 Reporting and Dissemination

1. Publication of the findings of the mixed methods process evaluation in a peer reviewed journal
2. Mixed methods process evaluation findings will also be referred to in the main trial paper
3. Conference presentations in national and international venues
4. Dissemination outputs, e.g., lay summaries, presentations to professional/patient bodies etc. Recommendations for implementation of BEARS

17.3 BEARS Include Program Development Grant project

Study Design	Mixed quantitative and qualitative methodology
Aims	• Measure the diversity of early recruitment, and compare with the population of deaf children with cochlear implants in the UK.<br>• Use an established RCT recruitment intervention protocol to optimise recruitment diversity<br>• Interview children and their families who chose not to take part in BEARS, clinicians, and patient representatives e.g. teachers to explore reasons for not participating<br>• Improve scale-up plan inclusivity
Data collection methods	Scoping review, quantitative analysis of BEARS recruitment data, in-depth interviews, paired interviews, focus groups
Sample size	Interviews: 10-15 children aged 8-16 with CI, 10-15 parents/carers, 10-12 clinicians and 10-12 family representatives

17.3.1 Background/rationale

BEARS Include will run in parallel with the programme grant. We want to improve the quality and value of the BEARS trial by ensuring recruitment is as diverse and representative as possible and also ensure our future plan to implement BEARS widely is inclusive. Our research seeks to serve all children with bilateral cochlear implants in the UK; under-served groups must be involved. The proposal augments and compliments the ongoing randomised controlled trial (RCT) by assessing and improving the ongoing screening and recruitment activities as well as informing the details of the implementation and scale up plan.

17.3.2 Study design

A mixed methods approach will be used to answer the Research Questions in 17.3.4.

17.3.3 Aims and objectives

We will evaluate the sociodemographic characteristics of families who have so far enrolled in the BEARS clinical trial to establish whether we have a representative sample of the population of deaf children with cochlear implants in the UK. A scoping review will help us to understand the sociodemographic characteristics of the population we wish to serve. We will use an established recruitment intervention method to try and improve the clinical trial recruitment. We will then augment the BEARS trial process evaluation work by exploring the barriers and facilitators to taking part in the BEARS clinical trial with stakeholders – focussing on people who declined participation. We will use this new learning to improve and co-create the scale-up plan.

17.3.4. Research Questions

1. What is the sociodemographic profile of children aged 8 to 16 years with cochlear implants in the UK? (WP1)
2. What are the sociodemographic characteristics of the families recruited to the BEARS clinical trial in the first 6 months? (WP2)
3. What are the barriers and facilitators to families taking part in the BEARS clinical trial? (WP3)
4. How can we use this learning to improve the scale-up plan for the roll out of BEARS? (WP4)

17.3.5 How this related to Process Evaluation (17.2)

After the BEARS clinical trial, we plan to scale up the use of the BEARS VR games across the UK for children and young people using cochlear implants, and we would like this to be as inclusive as possible. We already have a scale-up plan template, which will iteratively change as we progress through the BEARS clinical trial and this new project.

We have a process evaluation integrated into the BEARS clinical trial. Process evaluation can help determine why an intervention works or fails and how it can be optimised; results can then be used to inform scale-up and implementation strategies. Currently, the clinical trial process evaluation examines barriers and facilitators to participant retention and adherence. Contextual factors are examined through qualitative interviews and quantitative measures with participants, caregivers and clinicians. These include factors such as socioeconomic status, ethnicity, prior experience with technology, home environment, user experience, confidence, motivation, parent-reported participant behaviour, willingness to incorporate BEARS into routine clinical practice, as well as the experience of either implementing (clinicians) or participating in (families) the wider trial. The process evaluation of course involves children and families who have consented to be in the BEARS trial. It is beyond the scope of our process evaluation to incorporate non-trial participants, and to explore the reasons for non-participation by these individuals. In contrast, the programme development work builds on and augments the process evaluation by exploring a new set of participants: those who declined to take part. This will allow for a full analysis of recruitment and inform positive approaches to improve inclusivity by implementing changes during the RCT. By identifying populations that are not being involved, we can quantitatively analyse the stage at which participants are screened out of the trial or decline to participate at a site level. We can also qualitatively explore the reasons for individual non-participation. This adds significant value to the existing programme as we will be able to utilise data from both non-trial and trial participants to improve future recruitment and implementation strategies following the trial. A process evaluation should be an independent process to the RCT, analysing and reporting data following the trial. In contrast, this programme development work will permit the opportunity to implement change during the RCT, to broaden recruitment so that it is more representative of the population that the intervention is intended to reach. Together these strands of development work can improve the impact of the trial.

17.3.6 Work plan

Figure 2 below sets out the stages of development work we are planning.

Figure 2. Our plans for the BEARS Include development project

BEARS Both Ears | NIHR | National Institute for Health and Care Research | UCL COMPREHENSIVE CLINICAL TRIALS UNIT | NHS Guy's and St Thomas' NHS Foundation Trust

BEARS Include

Development work: what will we do?

Scoping review — WP1
What is the sociodemographic profile of children with CI in the UK?

Quantitative work — WP2
Analysis of sociodemographic characteristics of first 6 months of programme grant recruitment data

Quantitative work — WP2
Analyse programme grant screening data at each site

Comparison — WP2
Compare data to UK population, each centre's catchment data (and data from WP1)

Workshop and changes — WP2
QRI-Two protocol to optimise programme grant recruitment

Qualitative work — WP3
Interviews with children and families who declined participation, clinicians and family representatives

Quantitative work — WP2
Analysis of last 7 months of programme grant recruitment data - did we make it more diverse?

Using the learning — WP4
Combine trial process evaluation and previous WPs to make the BEARS NHS roll-out more inclusive

17.3.6.1 WP1: Establish the demographic characteristics of deaf children with cochlear implants in the UK: a scoping review

Researchers: Helen Cullington, Sandra Driver, Sarah Somerset, Deborah Vickers, Trishna Chauhan (patient and researcher)

Research question 1: What is the sociodemographic profile of children aged 8 to 16 years with cochlear implants in the UK?

17.3.6.1.1 Method

We will use the methodological framework proposed by Arksey and O'Malley [97] for scoping reviews, and report according to the PRISMA-ScR checklist [98]. We will incorporate recommendations given by Levac et al [99] to clarify and enhance each stage, also incorporating quality appraisal of evidence. One key consideration is that the research question may evolve during the searching process as the team become more familiar with the available evidence. All children with cochlear implants in the UK are cared for by one of 16 paediatric cochlear implant centres.

We will definitely approach all UK CI centres to share anonymised data and may use a Freedom of Information request. We anticipate that they collect data about ethnicity, sex, home postcode etc; we would also like to know about family size, educational attainment, disability status and home language if possible.

We will collect data that the centres share and may use a Freedom of Information request if required. Other sources of data are charities, the Consortium for Research in Deaf Education, the UK national hearing implant registry, local authorities, and schools.

17.3.6.1.2 Inclusion criteria

- Any literature, reports, statistics, figures, studies, policies, documents, grey literature, articles etc which share any demographic characteristics of deaf children with CI
- Published in the last 10 years (2016 onwards)
- Data from the UK population

The output of this work package will be a summary of available evidence and whether it is valid and generalisable. If we find gaps in the available evidence, we will make recommendations for future reporting and highlight any areas of missing information.

17.3.6.2 WP2: Establish the sociodemographic characteristics of participants recruited to the BEARS clinical trial, and use QRI-Two methods to identify hidden challenges and optimise recruitment number and diversity

Researchers: Fleur Corbett, Marcus Jepson, Sandra Driver, Deborah Vickers and Helen Cullington

Research question 2: What are the sociodemographic characteristics of the families recruited to the BEARS clinical trial in the first 6 months?

17.3.6.2.1 Method

The first six months of recruitment data will be quantitatively analysed. The data we will have are:

- Age, sex, ethnicity, socioeconomic status as a proxy from home postcode (from BEARS screening appointment CRF)
- Home language, parent/carer highest educational level and occupation, number of siblings (from BEARS baseline appointment CRF)

We will then compare the sociodemographic profile of recruited children with each centre's catchment area and the UK population to identify populations that are not being engaged. If available, we will also examine the sociodemographic profile of children screened at each site. The data we will obtain from each centre will be group data describing the population they serve – ethnicity, sex, postcodes. If WP1 reveals any quantitative data, that will also be used for comparison.

Data regarding the screening of children with bilateral CI, eligibility for participation, and clinician contact with eligible families will be analysed for each trial site using the QuinteT SEAR framework [100] to determine:

1. The percentage of children that the clinicians consider eligible for the BEARS clinical trial and reasons for ineligibility of others
2. The percentage of eligible children and families:
    - Contacted, and reasons why others not contacted
    - Who consented to participate
    - Who declined to participate, and reasons why declined if known
    - Who did not respond

We will follow the QRI-Two protocol [101] to identify and estimate the scale of 'hidden' barriers in the recruitment strategy. These barriers may include inconsistencies in participant information, clinician discomfort or emotion related to beliefs in the effectiveness of the intervention, or any perceived conflict with their clinical roles. Depending on the above findings, rapid content analysis of BEARS trial documentation (patient information materials, consent forms, protocol) may be required; input from our PPIEP group will help here. As knowledge about the sociodemographic profile of participants and recruitment process is generated, findings from the analysis of the initial recruitment data will be presented to the BEARS trial team, site clinicians, and PPIEP lead at a workshop (can be held online). We will work together to devise remedial actions and optimise the recruitment strategy in line with the INCLUDE recommendations [102], with the aim of improving recruitment within the remaining timeframe of the clinical trial . The INCLUDE recommendations are to ensure:

1. Eligibility criteria and recruitment pathway do not limit participation in unintended ways
2. Trial materials are developed with inclusion in mind
3. Trial staff are culturally competent.
4. Trusting partnerships with community organisations that work with ethnic minority group

we may also need to:

1. Change aspects of the protocol (even something as simple as changing the times of research sessions may facilitate inclusion for some families)
2. Change study materials e.g., advertising
3. educate and support clinicians. For example, if we find clinician gate-keeping has occurred, we will use education methods to address and decrease this [103].

We will make full use of the toolkit for Increasing participation of black Asian and minority ethnic (BAME) groups in health and social care research [104]. The final stage of WP2 will occur , after BEARS recruitment ends. We will quantitatively analyse the final 6 months of recruitment data to assess whether the actions of WP2 improved recruitment diversity.

17.3.6.3 WP3: Explore barriers and facilitators to families taking part in the BEARS clinical trial and how these might impact scale-up

Researchers: Ruth Nightingale and Sarah Somerset
Research question 3: What are the barriers and facilitators to families taking part in the BEARS clinical trial?

17.3.6.3.1 Method

The BEARS programme grant process evaluation explores factors that facilitate or hinder the use of the VR games at home – these insights will be used to inform the scale-up plan. WP3 will look specifically at participants who declined participation in the BEARS trial. We want to understand more about the lived experience of those declining participation and the reasons for their lack of engagement. This joined up approach of learning from two distinct groups will really optimise our future scale-up plans. We will use qualitative semi-structured interviews, paired interviews and focus groups with families of children with CI, clinicians and family representatives/stakeholders such as teachers and volunteers. We will engage with the PPIEP lead and PPIEP group to understand how we can engage those underrepresented groups from the trial and implement this into future plans for BEARS scale-up. We want to explore why children and or families do not engage with the BEARS intervention, covering:

1. Barriers and facilitators to family participation in the trial
2. Barriers and facilitators to the use of technology (i.e. using BEARS at home). If BEARS becomes part of routine care, what would support them to use it (scale-up)

We will ask clinicians what underpins their decision-making around the screening process, who they invite to participate, their perceptions of why families decline to take part, and how this could impact scale-up. We will ask family representatives their perceptions of why families decline to take part in research, the use of technology such as a VR rehabilitation tool for children, and how this might impact scale-up.

17.3.6.3.2 Theoretical framework

Scaling up and implementing BEARS in clinical practice will require changes in behaviour at individual (e.g. child, parent, clinician), collective and organisational levels. Therefore, the Theoretical Domains Framework (TDF), a synthesis of 33 behaviour change theories into 14 domains [105], will inform WP3. Using the TDF will enable us to conceptualise the barriers and facilitators to change, and together with findings from the process evaluation (conducted in the BEARS trial), will inform WP4 [106].

17.3.6.3.3 Participants and setting

Data will be collected across all trial sites. Inclusion criteria:

- Children/young people with CI and their parents who are eligible for the BEARS trial but declined to take part
- Clinicians/gatekeepers (e.g. research audiologists, rehabilitationists) who are responsible for screening and decision-making about whether child/family 1) meets the inclusion criteria, and 2) is subsequently invited to take part in the trial

- Family representatives/stakeholders from across the UK (e.g. teachers of the deaf, members of the voluntary sector such as ambassadors from the NDCS, the Cochlear Implanted Children's Support group (CICS), and regional deaf societies).

17.3.6.3.4 Sampling and recruitment

Purposive sampling will be used to achieve maximum variation among participants in relation to:

1. children's age, sex, ethnicity, study site, home language and family socioeconomic status
2. clinicians' role, study site and research experience
3. family representatives' role, organisation and area of the UK

We aim to recruit 10-15 children aged 8-16 with CI, 10-15 parents/carers, 10-12 clinicians and 10-12 family representatives. The final sample size will be determined by reaching data saturation. Children, parents and clinicians will be recruited from the study sites that are taking part in the BEARS trial. If necessary, we will use social media (e.g. British Cochlear Implant Group (BCIG), NDCS, CICS) to recruit families who declined to take part in the trial. Family representatives will be recruited via various methods including through the British Association of Teachers of Deaf Children and Young People (BATOD) network, CICS, NDCS, BCIG and social media. The letter of invitation for the BEARS project is amended to include text asking the family to say why they do not want to take part (either by email, phone, QR code/link to web questions on a Microsoft Form). The family can include their email address/phone number if they want to take part; otherwise, their reasons for not taking part remain anonymous.

17.3.6.3.5 Data collection

A mixture of qualitative methods including in-depth semi-structured individual interviews, paired interviews and focus groups will be used, as appropriate to each participant. Data collection will take place either virtually/online via videoconference, or in person depending on participants' preferences. Interviews/focus groups will be recorded with consent/assent, using an encrypted digital voice recorder. We will discuss with the ethics committee whether verbal consent at the start of the interview would be appropriate; we do not want to introduce additional steps that may put people off taking part in the research - especially as these are families who have already said no to the BEARS trial. We will make the study information as simple and accessible as possible and will translate where required. The participant children are eligible for BEARS, so have sufficient understanding of English. If a parent requires translation, we will provide.

Interviews are expected to last between 30-60 minutes and focus groups between 60-90 minutes. All interviews/focus groups will be transcribed by a UCL-approved, GDPR compliant transcription service. Interviews will be guided by a topic guide informed by the TDF and developed with input from the PPIEP group. All children taking part in an interview will be offered a £10 voucher.

17.3.6.3.6 Data analysis

Data will be analysed thematically using the Framework approach [96]. This matrix-based analytic method is flexible and systematic and facilitates rigorous and transparent data management. The domains and constructs of the TDF will inform our analysis and interpretation of the data.

17.3.6.4 WP4: Amend the BEARS scale-up plan based on new learning

Researchers: Helen Cullington, Fleur Corbett and Deborah Vickers

Research question 4: How can we use this learning to improve the scale-up plan for the roll out of BEARS?

Preliminary data from the process evaluation will be used to directly complement the findings from WPs 1 to 3 to enable iterative refinement of the scale-up and implementation strategy. The scale-up and implementation strategy seeks to optimise the trial approach for NHS adaptation of the BEARS games as patient self-managed technology- based care, ensuring equity and inclusivity. This strategy is subject to iterative improvement from new learning and informed by Normalisation Process Theory (NPT) [89, 90], which comprises four key concepts:

1. Coherence: how clinicians and patients understand BEARS
2. Cognitive participation: buy-in and support for BEARS
3. Collective action: operational work required for BEARS
4. Reflexive monitoring: how BEARS is appraised and how work can be configured to enable it

We will also incorporate the programme grant's early findings from the Normalisation MeAsure Development (NoMAD) questionnaire [91]. A significant part of the latter months of the main BEARS trial focusses on implementation and roll out. The new learning on diversity from this project will augment the approach we are already taking.

We will continue to involve our diversity PPIEP group and will obtain feedback from them and other stakeholders as the scale-up plan evolves.

17.3.7 Future work plans

We hope to roll out the BEARS VR games in the NHS after the programme grant is finished. This will involve a change in NHS policy and an arrangement for commissioning the equipment. This will be informed by the programme grant economic analysis.

17.3.8 Patient and Public Involvement

PPI advisory groups consisting of DHH children/young people, and parents who have a child with an implant, have been established to advise on the design and delivery of the study. PPI contributors have been involved in different aspects of the study including advising on the interview topic guides and open text/qualitative survey questions, and will continue to have input to the study, including qualitative data analysis and dissemination.

17.3.9 Dissemination

This project has diverse stakeholders - patients, the public, clinicians, teachers, funders, commissioners, managers, service leads, the NHS, hearing researchers, diversity researchers, cochlear implant companies, VR companies etc. We already have a communications plan for the BEARS programme grant with ideas of how to reach each sector. We have made a new section on our website (https://tinyurl.com/BothEars) covering Diversity; we will keep this updated.

We will produce two peer-reviewed publications (based on WP1 and WP3), presentations at the NDCS and the BCIG conferences in 2025, and newsletter articles for the National Cochlear Implant Users Association (NCIUA) and CICS.

It has worked well for us previously to co-present with a patient; we will continue this for future conferences.

We will produce an easy-read postcard to share our results with patients and the public. We will print 1000 and send bundles to all UK cochlear implant centres for their waiting rooms, for inclusion in NCIUA and CICS mailings, and our social media (@BEARS_CI). We are particularly keen to disseminate the findings to the participants in this research and will ask them for contacts in their own communities where it may be appropriate to give out postcards. Our PPIEP group will also be involved in dissemination. Including a diverse population in research is not just applicable to cochlear implants, or indeed deafness. We would like to share our learning more widely - to encourage other researchers to focus on research participant diversity. We will offer seminars for audiology teaching programs (both undergraduate and postgraduate) to influence a future generation of researchers.

17.3.10 Patient and public involvement and engagement

We continue to have access to the established BEARS PPIEP group. Since this development work focuses on equality, diversity and inclusivity, we have set up a new PPIEP group. The six members cover a wide age range and are:

- Two parents (from different families) of children who use cochlear implants. One identifies as Muslim; one organises a group for deaf children and families in Cardiff
- Two adults with cochlear implants (both identify as part of ethnic minority communities)
- Two members of the public - both with experience of PPIEP (one identifies as part of a minority community) The PPIEP lead is co-applicant Trishna Chauhan.

We will hold an introductory meeting in December 2023 to set out terms and co-design the payment policy. We will ask people how they want to be compensated, following NIHR guidance on reward and payment. Meetings will then be held monthly online, with communication support/captioning/interpretation as required. We will talk to each PPIEP member individually about training and development needs.

We have the support of the Southampton Research Design Service and the Wessex Public Involvement Network. We will follow the UK standards for Public Involvement.

Members of the PPIEP group will be asked for their input to study documentation (consent/assent form, information sheet). As we are committed to engaging 'hard to reach' groups, it will be particularly important that documents are accessible and not off-putting to potential participants. PPIEP input will also be sought for the recruitment intervention analysis in WP2 - the PPIEP lead will attend the workshop, planning the interview questions in WP3, the scale-up plan (WP4), and dissemination (particularly to patients). Further details in the Work Plan (WP2, 3 and 4).

We are committed to maintaining a feedback cycle between the research team and the PPIEP group to develop relationships, increases confidence and motivation. The lead applicant Helen Cullington will take responsibility for providing feedback - either via the PPIEP lead or directly to the contributor. She will also keep a log of 'Did this lead to change?' to track the impact of our PPIEP.

17.3.11 Ethics

We are submitting an amendment to the ethics in the following ways: we do not want to miss out on early months of recruitment data:

- Change to the invitation letter asking if parents/carers/children and young people who say no to BEARS would say their reasons on an online form and/or be willing to be contacted to explain their reasons
- To recruit parents/carers/children and young people/clinicians/family representatives to take part in interview/focus group (additional PIS and consent/assent forms needed)
- To use already-collected BEARS data for additional analysis
- To collect demographic data on the pre-screening log
- To collect each site's pre-screening log without participant identifiable

18 Protocol Amendments

Table 6. Summary of Protocol Amendments

Protocol version	Protocol date	Summary of changes
v1.0	19th December 2022	Initial version
v2.0	11th August 2023	1. Deletion of Exclusion criterion 9 and 10.<br>2. The VFS-C at 6 and 9 months has been removed.<br>3. Randomisation has moved from the screening appointment to Baseline, after the outcome assessments have been completed.<br>4. Section 17.2.12.2: An additional sampling criterion has been added: Participant site.<br>5. The words female and women have been removed from the protocol when referring to pregnancy.<br>6. More information has been provided in the protocol section 7.6 and 7.7 about data storage.
v3.0	16th May 2024	Under section 8.2.2 the description of the categories for each randomisation factor have been removed from the protocol as per guidance from the trial statistician.
v4.0	9th August 2024	1. Removing the 6/9-month follow-ups and any information relating to them<br>2. Changing the protocol window at 3/12-month follow-ups to -2weeks +6 weeks<br>3. Adding supplementary text to section 3.1.2 to provide guidance on how to categorise a severe to profound sensorineural hearing loss<br>4. Added BEARS Include Program Development work as an additional sub-study in 17.3<br>5. Removal of usual care arm interviews from Process Evaluation qualitative interviews in section 17.2.12.2<br>6. Removal of two sampling criteria for Process Evaluation qualitative interviews (change in Spatial Speech Text score at 3 months and period of time before severe to profound deafness) in section 17.2.12.2.
v5.0	11th July 2025	Updated Sponsor and CI email addresses due to switch to nhs.net
v6.0	24th September 2025	Update to the trial recruitment target. This has changed from 384 to 272.<br>Update to section 8.1: Sample size due to the change in recruitment target.<br>Additional section 5.3 added to explain the trial pathway of participants recruited after the 1st November 2025.
v7.0	14th July 2026	1. Removal of the YBHRQL-proxy outcome measure<br>2. Updates to the main RCT and Health Economic analyses, factoring in the removal of the YBHRQL-proxy and align with the current statistical analysis plan proposals

		3. Minor updates to the PDG data collection and analysis plans<br>4. Minor updates to PE qualitative data collection<br>5. Minor typographical errors corrected and updates made throughout the protocol

19 References

1. Chan, A.-W., et al., *SPIRIT 2013 statement: defining standard protocol items for clinical trials.* Annals of internal medicine, 2013. **158**(3): p. 200-207.
2. Chan, A.-W., et al., *SPIRIT 2013 explanation and elaboration: guidance for protocols of clinical trials.* BMJ : British Medical Journal, 2013. **346**: p. e7586.
3. Dunn, L. and D. Dunn, *The British Picture Vocabulary Scale Manual.* 3rd ed. 2009, UK: GL Assessment. .
4. Stevens, K., *Valuation of the Child Health Utility 9D Index.* PharmacoEconomics, 2012. **30**(8): p. 729-747.
5. Chung, C.C.Y., et al., *Client Service Receipt Inventory as a standardised tool for measurement of socio-economic costs in the rare genetic disease population (CSRI-Ra).* Scientific reports, 2021. **11**(1): p. 23837-23837.
6. Horsman, J., et al., *The Health Utilities Index (HUI®): concepts, measurement properties and applications.* Health and Quality of Life Outcomes, 2003. **1**(1): p. 54.
7. Rapley, T., et al., *Improving the normalization of complex interventions: part 1 - development of the NoMAD instrument for assessing implementation work based on normalization process theory (NPT).* BMC Medical Research Methodology, 2018. **18**(1): p. 133.
8. Macleod, A. and Q. Summerfield, *A procedure for measuring auditory and audiovisual speech-reception thresholds for sentences in noise: Rationale, evaluation, and recommendations for use.* British Journal of Audiology, 1990. **24**(1): p. 29-43.
9. Park Lisa, R., et al., *Spatial Release From Masking in Pediatric Cochlear Implant Recipients With Single-Sided Deafness.* American Journal of Audiology, 2021. **30**(2): p. 443-451.
10. Salorio-Corbetto, M., et al., *Evaluating Spatial Hearing Using a Dual-Task Approach in a Virtual-Acoustics Environment.* Frontiers in Neuroscience, 2022. **16**.
11. Hornsby Benjamin, W.Y., et al., *Development and Evaluation of Pediatric Versions of the Vanderbilt Fatigue Scale for Children With Hearing Loss.* Journal of Speech, Language, and Hearing Research, 2022. **65**(6): p. 2343-2363.
12. Summerfield, A.Q., P.T. Kitterick, and A.M. Goman, *Development and Critical Evaluation of a Condition-Specific Preference-Based Measure Sensitive to Binaural Hearing in Adults: The York Binaural Hearing–Related Quality-of-Life System.* Ear and Hearing, 2022. **43**(2).
13. Education, C.f.R.i.D., *Report on 2017 Survey on Educational Provision for Deaf Children*. 2017.
14. Excellence, N.I.f.H.a.C., *Cochlear implants for children and adults with severe to profound deafness: Technology appraisal guidance (TA566)* 2019: https://www.nice.org.uk/guidance/ta566.
15. Group, B.C.I., *CI activity 2017*. 2017: https://www.bcig.org.uk/wp-content/uploads/2018/02/Overall-data.pdf.
16. Blauert, J., *Spatial Hearing: The Psychophysics of Human Sound Localization*. 1997: MIT Press
17. Köbler, S. and U. Rosenhall, *Horizontal localization and speech intelligibility with bilateral and unilateral hearing aid amplification.* International Journal of Audiology, 2002. **41**(7): p. 395-400.
18. Noble, W. and S. Gatehouse, *Effects of bilateral versus unilateral hearing aid fitting on abilities measured by the Speech, Spatial, and Qualities of Hearing scale (SSQ).* International Journal of Audiology, 2006. **45**(3): p. 172-181.
19. McAlpine, D., D. Jiang, and A.R. Palmer, *A neural code for low-frequency sound localization in mammals.* Nature Neuroscience, 2001. **4**(4): p. 396-401.
20. Bronkhorst, A.W., *The cocktail-party problem revisited: early processing and selection of multi-talker speech.* Attention, Perception, & Psychophysics, 2015. **77**(5): p. 1465-1487.
21. Nabelek Anna, K. and J.M. Pickett, *Monaural and Binaural Speech Perception through Hearing Aids under Noise and Reverberation with Normal and Hearing-Impaired Listeners.* Journal of Speech and Hearing Research, 1974. **17**(4): p. 724-739.

22. McArdle, R.A., et al., *Are Two Ears Not Better Than One?* J Am Acad Audiol, 2012. **23**(03): p. 171-181.
23. Bat-Chava, Y. and E. Deignan, *Peer Relationships of Children With Cochlear Implants.* The Journal of Deaf Studies and Deaf Education, 2001. **6**(3): p. 186-199.
24. Stevenson, J., et al., *Emotional and behavioural difficulties in children and adolescents with hearing impairment: a systematic review and meta-analysis.* European Child & Adolescent Psychiatry, 2015. **24**(5): p. 477-496.
25. Sarant, J., et al., *Bilateral Versus Unilateral Cochlear Implants in Children: A Study of Spoken Language Outcomes.* Ear and Hearing, 2014. **35**(4).
26. Boons, T., et al., *Effect of Pediatric Bilateral Cochlear Implantation on Language Development.* Archives of Pediatrics & Adolescent Medicine, 2012. **166**(1): p. 28-34.
27. Sparreboom, M., et al., *Long-term outcomes on spatial hearing, speech recognition and receptive vocabulary after sequential bilateral cochlear implantation in children.* Research in Developmental Disabilities, 2015. **36**: p. 328-337.
28. Lovett, R., et al., *Bilateral or unilateral cochlear implantation for deaf children: An observational study.* Archives of disease in childhood, 2009. **95**: p. 107-12.
29. Beijen, J.-W., A.F.M. Snik, and E.A.M. Mylanus, *Sound Localization Ability of Young Children With Bilateral Cochlear Implants.* Otology & Neurotology, 2007. **28**(4).
30. Litovsky, R.Y., *Review of recent work on spatial hearing skills in children with bilateral cochlear implants.* Cochlear Implants International, 2011. **12**(sup1): p. S30-S34.
31. Grieco-Calub, T.M., R.Y. Litovsky, and L.A. Werner, *Using the Observer-Based Psychophysical Procedure to Assess Localization Acuity in Toddlers Who Use Bilateral Cochlear Implants.* Otology & Neurotology, 2008. **29**(2).
32. Zheng, Y., S.P. Godar, and R.Y. Litovsky, *Development of Sound Localization Strategies in Children with Bilateral Cochlear Implants.* PLOS ONE, 2015. **10**(8): p. e0135790.
33. Killan, C.F., et al., *The effect of early auditory experience on the spatial listening skills of children with bilateral cochlear implants.* International Journal of Pediatric Otorhinolaryngology, 2015. **79**(12): p. 2159-2165.
34. Grieco-Calub, T.M. and R.Y. Litovsky, *Sound localization skills in children who use bilateral cochlear implants and in children with normal acoustic hearing.* Ear and hearing, 2010. **31**(5): p. 645-656.
35. Jacobs, E., et al., *Benefits of simultaneous bilateral cochlear implantation on verbal reasoning skills in prelingually deaf children.* Research in Developmental Disabilities, 2016. **58**: p. 104-113.
36. Hughes, K.C. and K.L. Galvin, *Measuring listening effort expended by adolescents and young adults with unilateral or bilateral cochlear implants or normal hearing.* Cochlear Implants International, 2013. **14**(3): p. 121-129.
37. Lammers, M.J.W., et al., *Bilateral cochlear implantation in children: A systematic review and best-evidence synthesis.* The Laryngoscope, 2014. **124**(7): p. 1694-1699.
38. Johnston, J.C., et al., *Bilateral paediatric cochlear implants: A critical review.* International Journal of Audiology, 2009. **48**(9): p. 601-617.
39. Taitelbaum-Swead, R. and L. Fostick, *Audio-visual speech perception in noise: Implanted children and young adults versus normal hearing peers.* International Journal of Pediatric Otorhinolaryngology, 2017. **92**: p. 146-150.
40. Anderson, S. and N. Kraus, *Auditory Training: Evidence for Neural Plasticity in Older Adults.* Perspectives on hearing and hearing disorders. Research and research diagnostics, 2013. **17**: p. 37-57.
41. Firszt, J.B., et al., *Localization training results in individuals with unilateral severe to profound hearing loss.* Hearing Research, 2015. **319**: p. 48-55.
42. Yu, F., et al., *Effects of Training on Lateralization for Simulations of Cochlear Implants and Single-Sided Deafness.* Frontiers in Human Neuroscience, 2018. **12**.

Docusign Envelope ID: 5CA44ABD-F65C-8DE8-80E3-4572550DF9E0

43. Irving, S. and D.R. Moore, *Training sound localization in normal hearing listeners with and without a unilateral ear plug.* Hearing Research, 2011. **280**(1): p. 100-108.
44. Keating, P., et al., *Behavioral training promotes multiple adaptive processes following acute hearing loss.* eLife, 2016. **5**: p. e12264.
45. Keating, P., J.C. Dahmen, and A.J. King, *Context-specific reweighting of auditory spatial cues following altered experience during development.* Current biology : CB, 2013. **23**(14): p. 1291-1299.
46. Keating, P., J.C. Dahmen, and A.J. King, *Complementary adaptive processes contribute to the developmental plasticity of spatial hearing.* Nature neuroscience, 2015. **18**(2): p. 185-187.
47. Keating, P. and A. King, *Developmental plasticity of spatial hearing following asymmetric hearing loss: context-dependent cue integration and its clinical implications.* Frontiers in Systems Neuroscience, 2013. **7**.
48. Carlile, S., *The plastic ear and perceptual relearning in auditory spatial perception.* Frontiers in Neuroscience, 2014. **8**.
49. Trapeau, R., V. Aubrais, and M. Schönwiesner, *Fast and persistent adaptation to new spectral cues for sound localization suggests a many-to-one mapping mechanism.* The Journal of the Acoustical Society of America, 2016. **140**(2): p. 879-890.
50. Watson, C.J.G., et al., *The Generalization of Auditory Accommodation to Altered Spectral Cues.* Scientific reports, 2017. **7**(1): p. 11588-11588.
51. Hofman, P.M., J.G.A. Van Riswick, and A.J. Van Opstal, *Relearning sound localization with new ears.* Nature Neuroscience, 1998. **1**(5): p. 417-421.
52. Stitt, P., L. Picinali, and B.F.G. Katz, *Auditory Accommodation to Poorly Matched Non-Individual Spectral Localization Cues Through Active Learning.* Scientific Reports, 2019. **9**(1): p. 1063.
53. Zahorik, P., et al., *Perceptual recalibration in human sound localization: Learning to remediate front-back reversals.* The Journal of the Acoustical Society of America, 2006. **120**(1): p. 343-359.
54. Godar, S.P. and R.Y. Litovsky, *Experience with bilateral cochlear implants improves sound localization acuity in children.* Otology & neurotology : official publication of the American Otological Society, American Neurotology Society [and] European Academy of Otology and Neurotology, 2010. **31**(8): p. 1287-1292.
55. Killan, C., et al., *Factors Affecting Sound-Source Localization in Children With Simultaneous or Sequential Bilateral Cochlear Implants.* Ear and Hearing, 2019. **40**(4).
56. Strøm-Roum, H., et al., *Sound localising ability in children with bilateral sequential cochlear implants.* International Journal of Pediatric Otorhinolaryngology, 2012. **76**(9): p. 1245-1248.
57. Mathew, R., et al., *Development of electrophysiological and behavioural measures of electrode discrimination in adult cochlear implant users.* Hearing Research, 2018. **367**: p. 74-87.
58. Skivington, K., et al., *A new framework for developing and evaluating complex interventions: update of Medical Research Council guidance.* BMJ, 2021. **374**: p. n2061.
59. Rayes, H., G. Al-Malky, and D. Vickers, *Systematic Review of Auditory Training in Pediatric Cochlear Implant Recipients.* Journal of Speech, Language, and Hearing Research, 2019. **62**(5): p. 1574-1593.
60. Vickers, D., et al., *Involving Children and Teenagers With Bilateral Cochlear Implants in the Design of the BEARS (Both EARS) Virtual Reality Training Suite Improves Personalization.* Frontiers in Digital Health, 2021. **3**.
61. Bizley, J.K., et al., *Simultaneous Assessment of Speech Identification and Spatial Discrimination: A Potential Testing Approach for Bilateral Cochlear Implant Users?* Trends in Hearing, 2015. **19**: p. 2331216515619573.
62. Picinali, L., M. Salorio-Corbetto, and D. Vickers, *Spatial release from masking assessment in virtual reality for bilateral cochlear implants users*. 2019.

63. Goman, A., *A comparison of bilateral cochlear implantation and bimodal aiding in severely-profoundly hearing-impaired adults: head movements, clinical outcomes, and cost-effectiveness.* 2014.
64. Cullington, H.E., et al., *United Kingdom national paediatric bilateral project: Results of professional rating scales and parent questionnaires.* Cochlear Implants International, 2017. **18**(1): p. 23-35.
65. Beecham, J.K. and M. Knapp. *Costing psychiatric interventions*. 1992.
66. Vickers, D.A., et al., *Preliminary assessment of the feasibility of using AB words to assess candidacy in adults.* Cochlear Implants International, 2016. **17**(sup1): p. 17-21.
67. Faulkner, A., S. Rosen, and T. Green, *Comparing live to recorded speech in training the perception of spectrally shifted noise-vocoded speech.* The Journal of the Acoustical Society of America, 2012. **132**(4): p. EL336-EL342.
68. Musiclarity. *Musiclarity* Available from: www.musiclarity.com.
69. Reactify. *Reactify Music* 2010; Available from: https://reactifymusic.com/.
70. Dorman, M.F., et al., *Sound Source Localization by Normal-Hearing Listeners, Hearing-Impaired Listeners and Cochlear Implant Listeners.* Audiology and Neurotology, 2016. **21**(3): p. 127-131.
71. Lewin, S., C. Glenton, and A.D. Oxman, *Use of qualitative methods alongside randomised controlled trials of complex healthcare interventions: methodological study.* BMJ, 2009. **339**: p. b3496.
72. Cathain, A., et al., *What can qualitative research do for randomised controlled trials? A systematic mapping review.* BMJ Open, 2013. **3**(6): p. e002889.
73. Dammeyer, J., M. Chapman, and M. Marschark, *Experience of Hearing Loss, Communication, Social Participation, and Psychological Well-Being Among Adolescents With Cochlear Implants.* Am Ann Deaf, 2018. **163**(4): p. 424-439.
74. Wie, O.B., et al., *Long-Term Language Development in Children With Early Simultaneous Bilateral Cochlear Implants.* Ear Hear, 2020. **41**(5): p. 1294-1305.
75. Stevenson, J., et al., *Language and reading comprehension in middle childhood predicts emotional and behaviour difficulties in adolescence for those with permanent childhood hearing loss.* J Child Psychol Psychiatry, 2018. **59**(2): p. 180-190.
76. Michael, R., J. Attias, and E. Raveh, *Cochlear Implantation and Social-Emotional Functioning of Children with Hearing Loss.* The Journal of Deaf Studies and Deaf Education, 2018. **24**(1): p. 25-31.
77. Terlektsi, E., et al., *Peer Relationship Experiences Of Deaf And Hard-Of-Hearing Adolescents.* The Journal of Deaf Studies and Deaf Education, 2020. **25**(2): p. 153-166.
78. Campbell, S., et al., *Purposive sampling: complex or simple? Research case examples.* Journal of Research in Nursing, 2020. **25**(8): p. 652-661.
79. Bailey, J., *First steps in qualitative data analysis: transcribing.* Family Practice, 2008. **25**(2): p. 127-131.
80. Ritchie, J., et al., *Qualitative research practice: a guide for social science students and researchers*. 2003, London: Sage.
81. Parkinson, S., et al., *Framework analysis: a worked example of a study exploring young people's experiences of depression.* Qualitative Research in Psychology, 2016. **13**(2): p. 109-129.
82. O'Cathain, A., E. Murphy, and J. Nicholl, *Three techniques for integrating data in mixed methods studies.* BMJ, 2010. **341**: p. c4587.
83. Johnson, R.E., A.L. Grove, and A. Clarke, *Pillar Integration Process: A Joint Display Technique to Integrate Data in Mixed Methods Research.* Journal of Mixed Methods Research, 2019. **13**(3): p. 301-320.
84. Moore, G.F., et al., *Process evaluation of complex interventions: Medical Research Council guidance.* BMJ : British Medical Journal, 2015. **350**: p. h1258.

85. Kaur, N., et al., *Practical mixed methods strategies used to integrate qualitative and quantitative methods in community-based primary health care research.* Family practice, 2019. **36**(5): p. 666-671.
86. Sawyer, S.M., et al., *The age of adolescence.* The Lancet Child & Adolescent Health, 2018. **2**(3): p. 223-228.
87. Bruni, O., et al., *Technology Use and Sleep Quality in Preadolescence and Adolescence.* Journal of clinical sleep medicine : JCSM : official publication of the American Academy of Sleep Medicine, 2015. **11**(12): p. 1433-1441.
88. Shaw, H., D.A. Ellis, and F.V. Ziegler, *The Technology Integration Model (TIM). Predicting the continued use of technology.* Computers in Human Behavior, 2018. **83**: p. 204-214.
89. May, C.R., et al., *Development of a theory of implementation and integration: Normalization Process Theory.* Implementation Science, 2009. **4**(1): p. 29.
90. Murray, E., et al., *Normalisation process theory: a framework for developing, evaluating and implementing complex interventions.* BMC Medicine, 2010. **8**(1): p. 63.
91. Finch, T.L., et al., *Improving the normalization of complex interventions: part 2 - validation of the NoMAD instrument for assessing implementation work based on normalization process theory (NPT).* BMC Medical Research Methodology, 2018. **18**(1): p. 135.
92. Goodman, R., *The Strengths and Difficulties Questionnaire: A Research Note.* Journal of Child Psychology and Psychiatry, 1997. **38**(5): p. 581-586.
93. Laugwitz, B., T. Held, and M. Schrepp. *Construction and Evaluation of a User Experience Questionnaire*. in *HCI and Usability for Education and Work*. 2008. Berlin, Heidelberg: Springer Berlin Heidelberg.
94. Wöbbekind, L., T. Mandl, and C. Womser-Hacker, *Construction and First Testing of the UX Kids Questionnaire (UXKQ): A Tool for Measuring Pupil's User Experience in Interactive Learning Apps using Semantic Differentials*, in *Mensch und Computer 2021*. 2021, Association for Computing Machinery: Ingolstadt, Germany. p. 444–455.
95. Davis, F.D., R.P. Bagozzi, and P.R. Warshaw, *User Acceptance of Computer Technology: A Comparison of Two Theoretical Models.* Management Science, 1989. **35**(8): p. 982-1003.
96. Ritchie, J.L.J.M.N.C.O.R., *Qualitative research practice : a guide for social science students and researchers*. 2014.
97. Arksey, H. and L. O'Malley, *Scoping studies: towards a methodological framework.* International journal of social research methodology, 2005. **8**(1): p. 19-32.
98. Tricco, A.C., et al., *Engaging policy-makers, health system managers, and policy analysts in the knowledge synthesis process: a scoping review.* Implement Sci, 2018. **13**(1): p. 31.
99. Levac, D., H. Colquhoun, and K.K. O'Brien, *Scoping studies: advancing the methodology.* Implementation Science, 2010. **5**(1): p. 69.
100. Wilson, C., et al., *Development of a framework to improve the process of recruitment to randomised controlled trials (RCTs): the SEAR (Screened, Eligible, Approached, Randomised) framework.* Trials, 2018. **19**(1): p. 50.
101. Donovan, J.L., et al., *Development of a new adapted QuinteT Recruitment Intervention (QRI-Two) for rapid application to RCTs underway with enrolment shortfalls-to identify previously hidden barriers and improve recruitment.* Trials, 2022. **23**(1): p. 258.
102. Dawson, S., et al., *Trial Forge Guidance 3: randomised trials and how to recruit and retain individuals from ethnic minority groups-practical guidance to support better practice.* Trials, 2022. **23**(1): p. 672.
103. Sharkey, K., et al., *Clinician gate-keeping in clinical research is not ethically defensible: an analysis.* J Med Ethics, 2010. **36**(6): p. 363-6.
104. Farooqi, A., et al., *Toolkit for Increasing participation of Black Asian and minority ethnic (BAME) groups in health and social care research*. 2018.
105. Cane, J., D. O'Connor, and S. Michie, *Validation of the theoretical domains framework for use in behaviour change and implementation research.* Implement Sci, 2012. **7**: p. 37.

Docusign Envelope ID: 5CA44ABD-F65C-8DE8-80E3-4572550DF9E0

106. Atkins, L., et al., *A guide to using the Theoretical Domains Framework of behaviour change to investigate implementation problems.* Implement Sci, 2017. **12**(1): p. 77.

Certificate Of Completion

Envelope Id: 5CA44ABD-F65C-8DE8-80E3-4572550DF9E0		Status: Completed
Subject: Complete with Docusign: BEARS_Protocol_v7.0_14 Jul 2026_Clean.pdf		
Source Envelope:		
Document Pages: 107	Signatures: 4	Envelope Originator:
Certificate Pages: 5	Initials: 0	Liz Arram
AutoNav: Enabled		90 High Holborn 2nd Floor London
EnvelopeId Stamping: Enabled		, London WC1V 6LJ
Time Zone: (UTC) Dublin, Edinburgh, Lisbon, London		elizabeth.arram@ucl.ac.uk
		IP Address: 144.82.114.234

Record Tracking

Status: Original<br>27 July 2026 | 11:33	Holder: Liz Arram<br>elizabeth.arram@ucl.ac.uk	Location: DocuSign

Signer Events	Signature	Timestamp
Dan Jiang<br>dan.jiang3@nhs.net<br>Security Level: Email, Account Authentication (Optional), Logged in	Signed by:<br>6FF49BD2EB784A0...<br>Signature Adoption: Drawn on Device<br>Using IP Address: 212.71.225.250	Sent: 27 July 2026 | 11:35<br>Viewed: 27 July 2026 | 11:40<br>Signed: 27 July 2026 | 11:41

Electronic Record and Signature Disclosure:
Accepted: 26 February 2026 | 13:04
ID: deda91b9-c485-4044-b9dd-60ef916789d7

Signer Events	Signature	Timestamp
Debi Vickers<br>dav1000@medschl.cam.ac.uk<br>Security Level: Email, Account Authentication (Optional), Logged in	DocuSigned by:<br>Debi Vickers<br>3F1655C1B25F485...<br>Signature Adoption: Pre-selected Style<br>Using IP Address: 82.20.18.111	Sent: 27 July 2026 | 11:35<br>Viewed: 27 July 2026 | 11:37<br>Signed: 27 July 2026 | 11:39

Electronic Record and Signature Disclosure:
Accepted: 14 December 2022 | 11:51
ID: 2d7befda-75f4-433b-a297-a941f84b1ecf

Signer Events	Signature	Timestamp
James Blackstone<br>j.blackstone@ucl.ac.uk<br>Head of Clinical Trial Operations<br>UCL<br>Security Level: Email, Account Authentication (Optional), Login with SSO	DocuSigned by:<br>4BCBCE549E8E46B...<br>Signature Adoption: Uploaded Signature Image<br>Using IP Address: 80.176.233.85	Sent: 27 July 2026 | 11:35<br>Viewed: 27 July 2026 | 11:44<br>Signed: 27 July 2026 | 15:01

Electronic Record and Signature Disclosure:
Not Offered via Docusign

Signer Events	Signature	Timestamp
Nick Freemantle<br>nicholas.freemantle@ucl.ac.uk<br>CCTU Director<br>Comprehensive Clinical Trials Unit (CCTU) at UCL<br>Security Level: Email, Account Authentication (Optional), Login with SSO	DocuSigned by:<br>Nick Freemantle<br>19AD28D4AC4F48B...<br>Signature Adoption: Pre-selected Style<br>Using IP Address: 193.223.71.26	Sent: 27 July 2026 | 11:35<br>Viewed: 27 July 2026 | 18:24<br>Signed: 27 July 2026 | 18:24

Electronic Record and Signature Disclosure:
Not Offered via Docusign

In Person Signer Events	Signature	Timestamp

Editor Delivery Events	Status	Timestamp

Agent Delivery Events	Status	Timestamp

Intermediary Delivery Events	Status	Timestamp

Certified Delivery Events	Status	Timestamp

Carbon Copy Events	Status	Timestamp

Witness Events	Signature	Timestamp

Notary Events	Signature	Timestamp

Envelope Summary Events	Status	Timestamps
Envelope Sent	Hashed/Encrypted	27 July 2026 | 11:35
Certified Delivered	Security Checked	27 July 2026 | 18:24
Signing Complete	Security Checked	27 July 2026 | 18:24
Completed	Security Checked	27 July 2026 | 18:24

Payment Events	Status	Timestamps

Electronic Record and Signature Disclosure

ELECTRONIC RECORD AND SIGNATURE DISCLOSURE

From time to time, Comprehensive Clinical Trials Unit (CCTU) at UCL (we, us or Company) may be required by law to provide to you certain written notices or disclosures. Described below are the terms and conditions for providing to you such notices and disclosures electronically through the DocuSign system. Please read the information below carefully and thoroughly, and if you can access this information electronically to your satisfaction and agree to this Electronic Record and Signature Disclosure (ERSD), please confirm your agreement by selecting the check-box next to 'I agree to use electronic records and signatures' before clicking 'CONTINUE' within the DocuSign system.

Getting paper copies

At any time, you may request from us a paper copy of any record provided or made available electronically to you by us. You will have the ability to download and print documents we send to you through the DocuSign system during and immediately after the signing session and, if you elect to create a DocuSign account, you may access the documents for a limited period of time (usually 30 days) after such documents are first sent to you. After such time, if you wish for us to send you paper copies of any such documents from our office to you, you will be charged a $0.00 per-page fee. You may request delivery of such paper copies from us by following the procedure described below.

Withdrawing your consent

If you decide to receive notices and disclosures from us electronically, you may at any time change your mind and tell us that thereafter you want to receive required notices and disclosures only in paper format. How you must inform us of your decision to receive future notices and disclosure in paper format and withdraw your consent to receive notices and disclosures electronically is described below.

Consequences of changing your mind

If you elect to receive required notices and disclosures only in paper format, it will slow the speed at which we can complete certain steps in transactions with you and delivering services to you because we will need first to send the required notices or disclosures to you in paper format, and then wait until we receive back from you your acknowledgment of your receipt of such paper notices or disclosures. Further, you will no longer be able to use the DocuSign system to receive required notices and consents electronically from us or to sign electronically documents from us.

All notices and disclosures will be sent to you electronically

Unless you tell us otherwise in accordance with the procedures described herein, we will provide electronically to you through the DocuSign system all required notices, disclosures, authorizations, acknowledgements, and other documents that are required to be provided or made available to you during the course of our relationship with you. To reduce the chance of you inadvertently not receiving any notice or disclosure, we prefer to provide all of the required notices and disclosures to you by the same method and to the same address that you have given us. Thus, you can receive all the disclosures and notices electronically or in paper format through the paper mail delivery system. If you do not agree with this process, please let us know as described below. Please also see the paragraph immediately above that describes the consequences of your electing not to receive delivery of the notices and disclosures electronically from us.

How to contact Comprehensive Clinical Trials Unit (CCTU) at UCL:

You may contact us to let us know of your changes as to how we may contact you electronically, to request paper copies of certain information from us, and to withdraw your prior consent to receive notices and disclosures electronically as follows:
To contact us by email send messages to: cctu.qa@ucl.ac.uk

To advise Comprehensive Clinical Trials Unit (CCTU) at UCL of your new email address

To let us know of a change in your email address where we should send notices and disclosures electronically to you, you must send an email message to us at cctu.qa@ucl.ac.uk and in the body of such request you must state: your previous email address, your new email address. We do not require any other information from you to change your email address.

If you created a DocuSign account, you may update it with your new email address through your account preferences.

To request paper copies from Comprehensive Clinical Trials Unit (CCTU) at UCL

To request delivery from us of paper copies of the notices and disclosures previously provided by us to you electronically, you must send us an email to cctu.qa@ucl.ac.uk and in the body of such request you must state your email address, full name, mailing address, and telephone number. We will bill you for any fees at that time, if any.

To withdraw your consent with Comprehensive Clinical Trials Unit (CCTU) at UCL

To inform us that you no longer wish to receive future notices and disclosures in electronic format you may:

i. decline to sign a document from within your signing session, and on the subsequent page, select the check-box indicating you wish to withdraw your consent, or you may;

ii. send us an email to cctu.qa@ucl.ac.uk and in the body of such request you must state your email, full name, mailing address, and telephone number. We do not need any other information from you to withdraw consent.. The consequences of your withdrawing consent for online documents will be that transactions may take a longer time to process..

Required hardware and software

The minimum system requirements for using the DocuSign system may change over time. The current system requirements are found here: https://support.docusign.com/guides/signer-guide-signing-system-requirements.

Acknowledging your access and consent to receive and sign documents electronically

To confirm to us that you can access this information electronically, which will be similar to other electronic notices and disclosures that we will provide to you, please confirm that you have read this ERSD, and (i) that you are able to print on paper or electronically save this ERSD for your future reference and access; or (ii) that you are able to email this ERSD to an email address where you will be able to print on paper or save it for your future reference and access. Further, if you consent to receiving notices and disclosures exclusively in electronic format as described herein, then select the check-box next to 'I agree to use electronic records and signatures' before clicking 'CONTINUE' within the DocuSign system.

By selecting the check-box next to 'I agree to use electronic records and signatures', you confirm that:

- You can access and read this Electronic Record and Signature Disclosure; and
- You can print on paper this Electronic Record and Signature Disclosure, or save or send this Electronic Record and Disclosure to a location where you can print it, for future reference and access; and
- Until or unless you notify Comprehensive Clinical Trials Unit (CCTU) at UCL as described above, you consent to receive exclusively through electronic means all notices, disclosures, authorizations, acknowledgements, and other documents that are required to be provided or made available to you by Comprehensive Clinical Trials Unit (CCTU) at UCL during the course of your relationship with Comprehensive Clinical Trials Unit (CCTU) at UCL.
