# Supplementary material for: Multicentre phase III randomised controlled trial to evaluate effectiveness of the Both EARS (BEARS) virtual reality training package to maximise hearing abilities in children and young people with bilateral cochlear implants: the BEARS protocol
Source: BMJ Open. 2026 Oct 27;16(10):e128310. doi: 10.1136/bmjopen-2026-128310 (PMC13723878; doi:10.1136/bmjopen-2026-128310)
Supplement: Supplementary file 3 [file bmjopen-16-10-s003.pdf]

Summary of Protocol Amendments

Protocol version	Protocol date	Summary of changes
v1.0	19th December 2022	Initial version
v2.0	11th August 2023	1. Deletion of Exclusion criterion 9 and 10.<br>2. The VFS-C at 6 and 9 months has been removed.<br>3. Randomisation has moved from the screening appointment to Baseline, after the outcome assessments have been completed.<br>4. Section 17.2.12.2: An additional sampling criterion has been added: Participant site.<br>5. The words female and women have been removed from the protocol when referring to pregnancy.<br>6. More information has been provided in the protocol section 7.6 and 7.7 about data storage.
v3.0	16th May 2024	Under section 8.2.2 the description of the categories for each randomisation factor have been removed from the protocol as per guidance from the trial statistician.
v4.0	9th August 2024	1. Removing the 6/9-month follow-ups and any information relating to them<br>2. Changing the protocol window at 3/12-month follow-ups to -2weeks +6 weeks<br>3. Adding supplementary text to section 3.1.2 to provide guidance on how to categorise a severe to profound sensorineural hearing loss<br>4. Added BEARS Include Program Development work as an additional sub-study in 17.3<br>5. Removal of usual care arm interviews from Process Evaluation qualitative interviews in section 17.2.12.2<br>6. Removal of two sampling criteria for Process Evaluation qualitative interviews (change in Spatial Speech Text score at 3 months and period of time before severe to profound deafness) in section 17.2.12.2.
v5.0	11th July 2025	Updated Sponsor and CI email addresses due to switch to nhs.net
v6.0	24th September 2025	Update to the trial recruitment target. This has changed from 384 to 272.<br>Update to section 8.1: Sample size due to the change in recruitment target.<br>Additional section 5.3 added to explain the trial pathway of participants recruited after the 1st November 2025.
v7.0	14th July 2026	1. Removal of the YBHRQL-proxy outcome measure<br>2. Updates to the main RCT and Health Economic analyses, factoring in the removal of the YBHRQL-

		proxy and align with the current statistical analysis plan proposals<br>3. Minor updates to the PDG data collection and analysis plans<br>4. Minor updates to PE qualitative data collection<br>5. Minor typographical errors corrected and updates made throughout the protocol
