# Supplementary material for: Multicentre phase III randomised controlled trial to evaluate effectiveness of the Both EARS (BEARS) virtual reality training package to maximise hearing abilities in children and young people with bilateral cochlear implants: the BEARS protocol
Source: BMJ Open. 2026 Oct 27;16(10):e128310. doi: 10.1136/bmjopen-2026-128310 (PMC13723878; doi:10.1136/bmjopen-2026-128310)
Supplement: Supplementary file 4 [file bmjopen-16-10-s004.pdf]

UCL COMPREHENSIVE CLINICAL TRIALS UNIT

BEARS CRF Pregnancy Notification

BEARS Both Ears

Participant ID ☐☐-☐☐☐

Month & Year of birth ☐☐-☐☐☐☐

1. Notification

Please notify UCL CCTU within 24 hours of site awareness of pregnancy.

1a. Site Name: ______________________________

1b. Date of site becoming aware of pregnancy:

D	D	M	M	M	Y	Y	Y	Y

1c. Date of report to UCL CCTU:

D	D	M	M	M	Y	Y	Y	Y

2. Trial intervention details

Which trial arm is the participant in? (✓)<br>***If 'Usual care' only, please ignore the remaining questions***	BEARS training package + Usual Care ☐<br>Usual Care ☐
If 'BEARS training package + Usual care',<br>has trial intervention commenced? (✓)	Yes ☐ No ☐
Date of first device use:	D D M M M Y Y Y Y
Date of most recent device use:	D D M M M Y Y Y Y

Sections 1 & 2 Completed by:

Name (Please Print)______________________ Signature ______________________

Date: d d m m m y y y y
