# Supplementary material for: Multicentre phase III randomised controlled trial to evaluate effectiveness of the Both EARS (BEARS) virtual reality training package to maximise hearing abilities in children and young people with bilateral cochlear implants: the BEARS protocol
Source: BMJ Open. 2026 Oct 27;16(10):e128310. doi: 10.1136/bmjopen-2026-128310 (PMC13723878; doi:10.1136/bmjopen-2026-128310)
Supplement: Supplementary file 5 [file bmjopen-16-10-s005.pdf]

BEARS

Both **EARS** training package (BEARS) to maximise hearing abilities in older children and teenagers with bilateral cochlear implants

**UCL CTU ID:**	**CTU/2019/330**
**Protocol versions:**	**7.0, 14 July 2026**
**Trial Registration #:**	**NCT05808543**
**Research Ethics Committee #:**	**23/YH/0046**

STATISTICAL AND HEALTH ECONOMICS ANALYSIS PLAN (SHEAP)

VERSION 0.8, 28TH JULY 2026

DISCLAIMER: THIS IS NOT A FINAL VERSION AND SUBJECT TO CHANGE UNTIL THE FIRST SUBSTANTIVE ANALYSIS

DRAFT

**Approved by:**	**Signature**	**Date**
Dr Deborah Vickers<br>Chief Scientific Investigator		
Professor Dan Jiang<br>Chief Investigator		
Kashfia Chowdhury<br>Oversight Statistician		
**Prepared by:**	Norin Ahmed<br>Trial Statistician<br>Email: n.mumtaz@ucl.ac.uk	
	Dr. Caroline S. Clarke<br>Principal Research Fellow in Health Economics<br>Email: caroline.clarke@ucl.ac.uk	
	Dr. Ekaterina Bordea<br>Senior Research Fellow<br>Email: e.bordea@ucl.ac.uk	

BEARS Both Ears

UCL COMPREHENSIVE CLINICAL TRIALS UNIT

TABLE OF CONTENTS

ABBREVIATIONS 3

1 INTRODUCTION 5

1.1 Background and rationale 5
1.2 Objectives 6
1.3 Estimand framework (primary outcome) 6

2 STUDY METHODS 7

2.1 Trial design 7
2.2 Randomisation 7
2.3 Sample size 7
2.4 Framework 8
2.5 Statistical interim analyses and stopping guidance 8
2.6 Timing of final analysis 8
2.7 Timing of outcome assessments 8

3 STATISTICAL PRINCIPLES 8

3.1 Confidence intervals and p-values 8
3.2 Analysis population and Missing Data 8

4 TRIAL POPULATION 9

4.1 Screening, recruitment, withdrawal/follow-up 9
4.2 Eligibility 9
4.3 Baseline participant characteristics 9

5 ANALYSIS 10

5.1 Outcome definitions 10
5.1.1 Primary Outcome 10
5.1.2 Secondary Outcomes 10
5.1.3 Health Economic Outcomes 10
5.1.4 Rationale and details for outcome measures 11
5.2 Analysis Methods 13
5.2.1 Adjustment factors 13
5.2.2 Primary Outcome Analysis 13
5.2.3 Secondary outcome analysis 14
5.2.4 Safety reporting 14
5.2.5 Subgroup Analysis 15
5.2.6 Exploratory analysis 15

6 HEALTH ECONOMICS ANALYSIS 15

7 REVISION HISTORY 18

8 APPENDICES 19

8.1 Dummy Tables 19

9 REFERENCES 23

DRAFT

ABBREVIATIONS

AE	Adverse Event
AR	Adverse Reaction
BATOD	British Association of Teachers of the Deaf
BEARS	Both EARS training package
BPVS	British Picture Vocabulary Scale
CAP	Categories Auditory Perception
CEA	Cost-Effectiveness Analysis
CEAC	Cost-Effectiveness Acceptability Curve
CEP	Cost-effectiveness Plane
CHU9D	Child Health Utility 9 Dimensions
CCTU	Comprehensive Clinical Trials Unit at UCL
CI	Chief Investigator
CI	Cochlear Implant
CRF	Case Report Form
CSRI	Client Service Receipt Inventory
CTCAE	Common Terminology Criteria for Adverse Events
DHH	Deaf or Hard of Hearing
DMEC	Data Monitoring and Ethics Committee
EC	Ethics Committee
EDC	Electronic Data Capture
GCP	Good Clinical Practice
GP	General Practitioner
GSTT	Guy’s and St Thomas’ NHS Foundation Trust
HE	Health Economist
HEAP	Health Economics Analysis Plan
HRA	Health Research Authority
HUI-3	Health Utilities Index Mark 3
ICER	Incremental Cost-Effectiveness Ratio
ICH	International Conference on Harmonisation
ICF	Informed Consent Form
IMPD	Investigational Medicinal Product Dossier

IRAS	Integrated Research Application System
ISF	Investigator Site File
ISRCTN	International Standard Randomised Controlled Trial Number
ITT	Intention to Treat
MRC	Medical Research Council
NAE	Notifiable Adverse Event
NHS	National Health Service
NIHR	National Institute for Health and Care Research
NOMAD	Normalisation Measure Development questionnaire
NPT	Normalisation Process Theory
PI	Principal Investigator
PIN	Participant Identification Number
PIS	Participant Information Sheet
PPI	Participant and Public Involvement
PSC	Programme Steering Committee
PSS	Personal Social Services
QA	Quality Assurance
QALY	Quality-Adjusted Life-Years
QC	Quality Control
QMMP	Quality Management and Monitoring Plan
QOL	Quality of Life
R&D	Research and Development
REC	Research Ethics Committee
SAE	Serious Adverse Event
SAP	Statistical Analysis Plan
SAR	Serious Adverse Reaction
SDQ	Strengths and Difficulties Questionnaire
SHEAP	Statistical and Health Economics Analysis Plan
SIR	Speech Intelligibility Rating
SOP	Standard Operating Procedure

DRAFT

Sp-ASL	Spatial Adaptive Sentence List
SRM	Spatial Release from Masking
SRT	Speech Reception Threshold
SSiN- VA	Spatial speech in Noise - Virtual Acoustics
TMF	Trial Master File
TMG	Trial Management Group
ToR	Terms of Reference
UCL	University College London
VFS-C	Vanderbilt Fatigue Scale: Child self-report version
YBHRQL-Y	York Binaural Hearing-Related Quality of Life questionnaire for young people/youths

DRAFT

1 INTRODUCTION

1.1 Background and rationale

Cochlear Implants (CIs) are medical devices implanted in the ears that send electricity to the hearing nerve, allowing people with significant deafness to hear. There are approximately 10,400 children aged under 20 years with severe-to-profound deafness in the UK, with cochlear implantation being the primary intervention. The National Institute for Health and Care Excellence recommends deaf children have bilateral cochlear implantation, to provide the advantages of hearing through two ears rather than one. Currently, 5426 people have bilateral CIs and approximately 475 new children implanted annually in the UK[1].

Speech communication is crucial for cognitive, social and language development and often takes place in complex noisy environments. For normal-hearing listeners, subtle differences in timing and level of sounds reaching each ear provide directional cues which contribute to the separation of speech from noise and the ability to attend to a particular speaker among other abilities. Children and teenagers are the largest groups of those bilateral implanted and spend substantial time in noisy environments, impacting on their speech and language development, education, and social well-being. Hearing impairment puts older children and teenagers with CIs at a greater risk of emotional and behavioural difficulties that impacts on social relationships with their peers.

Although language development, sound localisation, tracking of moving sound sources speech-in-noise perception and listening effort are better for people with bilateral CIs than for those with a unilateral implant[2, 3] these skills remain far below those of normally-hearing children. People with bilateral CIs often struggle to understand speech in noise even with visual cues[4].

A large body of research demonstrates that sound localisation can improve with training, underpinned by plasticity-driven changes in the auditory pathways for children and adults[5-10]. Paediatric CI users report that computer-based rehabilitation assessments would better fit their lifestyles and support their learning. By providing engaging training using videogames that are relevant to young people, it is expected that positive brain changes can occur to maximise the use of spatial-hearing skills. Implementing training to improve spatial hearing in older children with bilateral CIs has the potential to improve auditory performance in real-life scenarios, which would have positive repercussions in social, emotional, and academic aspects of life. However, no training programme has been implemented and evaluated for young people with bilateral CIs.

The **B**oth **Ears** training package (BEARS) of virtual-reality games to train spatial hearing in young people with bilateral CIs. The BEARS training package comprises of three games addressing different hearing functions: speech-in-noise perception, music listening and sound-source localisation. Each game is based on an audio-visual task performed through a virtual-reality interface. Players are automatically guided through on-screen visual prompts to support the gameplay with feedback given on their performance. Players progress through levels of increasing difficulty.

DRAFT

1.2 Objectives

The primary objective of the BEARS study is to determine whether the use of spatial-listening training for 3-months delivered via the BEARS training package in addition to usual care, when compared to usual care alone, improves spatial speech-in-noise perception when assessed at three months (accounting for baseline).

1.3 Estimand framework (primary outcome)

**Characteristic of estimand (primary outcome)[11]**	**Definition and method of analysis[11]**
Population	Participant is a simultaneous or sequential bilateral cochlear implant user, has stable programmes, has had at least two usual care checks/clinical appointments with stable aided levels and aged 8-16 years (inclusive).
Treatment conditions	3 months of spatial-listening training delivered via the Both EARS training package (BEARS) in addition to usual care versus Usual Care (routine rehabilitation clinical care pathway).
Endpoint	Speech-in-noise perception score (% correct overall task) at 3 months.
Summary measure	Mean difference between arms accounting for the baseline score.
Intercurrent events	
➢ Withdrawal (temporary or definitive)	Treatment policy strategy
➢ Different levels of intervention exposure	Treatment policy strategy
➢ Failure to start intervention	Treatment policy strategy
➢ Out-of-window primary outcome visit	While-on-treatment strategy

DRAFT

2 STUDY METHODS

2.1 Trial design

BEARS is a multi-centre, open label, randomised controlled, phase III, superiority trial. Participants aged 8-16 years (inclusive) with bilateral cochlear implants will be randomised in a 1:1 ratio.

2.2 Randomisation

Participants will be randomised in a 1:1 ratio to either the BEARS or usual care intervention arm. A concealed minimisation process according to the minimisation factors (sex, school age, school setting, severe-to-profound congenital hearing loss and device allocation). This will ensure that balance across the two intervention groups will be achieved. The minimisation algorithm will incorporate a random element to maximise balance in the stratifying variables between the randomised groups.

Minimisation factors will include:

- Sex (female/male)
- School age group (primary/secondary)
- School setting (boarding during the week/mainstream)
- Severe-to-Profound Congenital Hearing Loss (Yes/No)
- Device allocation: Head Mounted Display /iPad (as decided by the PI or delegate)

2.3 Sample size

The Primary outcome for the BEARS trial is the difference between the intervention groups in speech-in-noise-perception score (% correct overall task) at 3 months, accounting for the participant's baseline score.

The original sample size was 153 evaluable participants per group required to detect the clinically important difference between treatment groups of 2.25 percentage points in change in speech-in-noise-perception score (obtained when the speech and noise separated by 30° compared to 0°), with 90% power and at the 5% level of significance (two-sided). The SD of change from baseline is estimated to be 6.05 percentage points, obtained from the Spatial-Speech-Test validation study. Mitigating loss to follow up or other challenges a 20% allowance raised this to a total of 192 participants per group totalling 384 participants.

The revised sample size of a total of 230 evaluable participants is based on detecting the clinically important difference between treatment groups of 2.25 percentage points in change in speech-in-noise-perception score with 80% power and at the 5% level of significance (two-sided), assuming the original SD in change from baseline of 6.05 percentage points. Based on observed withdrawal proportion in the trial, we revised the allowance for loss to follow-up to 15%. The revised required sample size is 136 per group, totalling 272 participants.

DRAFT

2.4 Framework

BEARS is a superiority trial, the primary purpose of which is to assess whether BEARS substantially improves hearing in participants with bilateral cochlear implants.

2.5 Statistical interim analyses and stopping guidance

There will be no interim analyses. Monitoring of the safety of the trial will be undertaken by the Data Monitoring and Ethics Committee (DMEC) which will have untrammelled access to the trial data, and whose work is governed by a separate charter.

2.6 Timing of final analysis

The last participant's primary endpoint data visit is expected in end of October 2026. All CRFs for primary endpoint data should be available within 7 days of this visit. Data query and cleaning will commence once this CRF is entered into the database.

The final analysis will start when all data for the primary endpoint is entered into the database and all corresponding queries are resolved.

2.7 Timing of outcome assessments

The timing of outcome assessments is provided in section 5.2 (Participant pathway) of the protocol.

3 STATISTICAL PRINCIPLES

3.1 Confidence intervals and p-values

All confidence intervals presented will be 95% and two-sided. The p-value of the statistical test will be reported at the 5% significance level, unless otherwise specified.

3.2 Analysis population and Missing Data

Analysis will be conducted on all participants randomised to either the BEARS or usual care intervention groups. Primary outcome analysis will follow the intention-to-treat (ITT) principle where all randomised participants are analysed in their allocated group, whether or not they receive their randomised intervention plan. All participants with reported outcome data within the time window defined in the protocol (-2 weeks/ +6 weeks) will be included in the primary analysis.

All efforts will be made to ensure that the primary outcome data are collected for all participants. In case of missing data, we will note the reasons for missingness and explore the

DRAFT

potential consequences of the loss through sensitivity analyses. There will be no imputation for missing data for any of the trial outcomes. An ITT analysis will be performed for all secondary outcomes. Outcomes at 12 months will be from a subgroup of participants who have reached the 12-month timepoint and have complete data only.

4 TRIAL POPULATION

4.1 Screening, recruitment, withdrawal/follow-up

Potential participants screened but not enrolled in the trial and reasons for exclusions will be reported, and recruitment will be presented by centre and month.

The number of participants who have been withdrawn or were unwilling to continue follow-up will be reported by treatment arm.

The throughput of participants from those screened to those who are enrolled and assessed for trial endpoints, and included in the analyses, will be summarised in a CONSORT flowchart.

4.2 Eligibility

Eligibility and inclusion/exclusion criteria are provided in section 3.1 and 3.2 of the protocol.

4.3 Baseline participant characteristics

The list of baseline characteristics to be summarised is provided in Table 1 of the Appendix.

Baseline characteristics will be summarised for all randomised participants in the study. Summary measures for the baseline characteristics will be presented as mean and standard deviation for continuous normally distributed (approximate) variables, medians and interquartile ranges for non-normally distributed continuous variables, and frequencies and percentages for categorical variables. We will plot histograms of continuous variables to assess normality.

Baseline characteristics will include the percentage of participants within each of the categories defined by the minimisation factors.

DRAFT

DRAFT

5 ANALYSIS

5.1 Outcome definitions

5.1.1 Primary Outcome

The Primary outcome for the BEARS trial is the difference between the intervention groups in speech-in-noise perception score (% correct overall task) at 3 months, accounting for the participant's baseline score. This is derived from the spatial speech in noise (SSiN-VA) test.

5.1.2 Secondary Outcomes

SSiN-VA test outcomes

- Speech-in-noise perception score (% correct of the overall task) at twelve months accounting for the baseline score.
- Relative localisation score (% correct) at three months and at twelve months accounting for the baseline score.
- Average reaction time for word 2 (measure of listening effort) for word identification selections at three months and at twelve months accounting for the baseline score.
- Average reaction time (measure of listening effort) for location shift selection at three months, and at twelve months accounting for the baseline score.
- Spatial index for word identification at three months and at twelve months accounting for the baseline score (measuring Spatial Release from Masking (SRM)). An average of the indexes across right and left ears will be used.
- Spatial index for relative localisation at three months and at twelve months accounting for the baseline score. An average of the indexes across right and left ears will be used.

Spatial ASL test outcomes

- Co-located SRT at three months and at twelve months, accounting for baseline.
- Average SRM (across right and left noise presentations) at three months and at twelve months, accounting for baseline.

British Picture Vocabulary Scale (BPVS) test outcome

Difference between arms in vocabulary age at twelve months, accounting for baseline vocabulary age, as per the BPVS standardised score and the difference between the chronological age and the age equivalent score.

VFS-C questionnaire outcome

- Difference between arms in listening-related fatigue score at three and 12 months, accounting for baseline.

5.1.3 Health Economic Outcomes

The health economic evaluation will calculate incremental cost per quality-adjusted life-year (QALY) gained by offering BEARS and usual care compared to usual care, from an NHS and Personal Social Services (PSS) and Local Education Provider perspective, over the twelve months of the trial.

Outcomes, along with the amounts of missing data, to be reported as part of this analysis are:

- Resource use in each arm, reported according to suitable categories from the CSRIs
- Mean cost per participant of delivering the intervention, in the BEARS arm only
- Mean pathway cost per participant in each arm (i.e., costs from CSRIs, plus intervention costs if applicable), adjusted for baseline values
- Mean utility values per participant calculated from the YBHRQL-Y self-complete responses, by timepoint and arm
- Mean utility values per participant calculated from the proxy HUI-3 responses, by timepoint and arm
- Mean utility values per participant calculated from the self-completed CHU-9D, by timepoint and arm
- Mean QALYs over 12-months per participant calculated from the YBHRQL-Y self-complete responses, by arm, adjusted for baseline values
- Mean QALYs over 12-months per participant calculated from the proxy HUI-3 responses, by arm, adjusted for baseline values
- Mean QALYs over 12-months per participant calculated from the self-complete CHU-9D responses, by arm, adjusted for baseline values
- Incremental cost-effectiveness ratio (ICER) for primary health economic analysis (i.e. using QALYs from YBHRQL-Y self-complete responses)
- Incremental cost-effectiveness ratio (ICER) for secondary health economic analysis (i.e., using QALYs from proxy HUI-3 responses)
- Incremental cost-effectiveness ratio (ICER) for secondary health economic analysis (i.e., using QALYs from self-complete CHU-9D responses)

DRAFT

5.1.4 Rationale and details for outcome measures

Speech-in-noise perception (*SSiN-VA)* score
The SSiN-VA is a version of the Spatial speech in noise test that is implemented in Virtual Acoustics. It was developed to simultaneously assess relative localisation and word identification performance. An overall percentage correct for word identification (speech perception) and relative localisation can be calculated from this.

Relative localisation score
This is a result obtained from the SSiN-VA. It is the score for the identification of location shift between the first and second word, presented as part of the SSiN-VA assessment. A % correct score can be obtained from each mean speaker location (location between the first and second word). The overall mean score would be obtained from averaging across all mean speaker locations.

Average reaction time (measure of listening effort) for word 2 identification and location shift

Derived from the SSiN-VA, participants will use a touchscreen to make their selections, with reaction times (in seconds) being recorded for each of the three responses (when identifying the two words and when identifying the location shift). Reaction times for word2 and for relative localisation will be used as a proxy for measuring listening effort. During the task, the words are presented from different speaker locations, and the background noise also varies by location.

Spatial index for word identification and relative localisation

This is a result obtained from the SSiN-VA, during the task, the words are presented from different speaker locations, and the background noise also varies by location. The listener's performance varies as a function of the distance between the speech and noise. For word identification, the participants may identify the first and second words on a touchscreen tablet, presented as part of the SSiN-VA assessment. A % correct score can be obtained from each speaker location. Overall mean score would be obtained from averaging across all locations.

Spatial ASL – Co-located Speech Reception Threshold (SRT)

During the Spatial ASL test, participants are presented with sentences in noise where the speech level is fixed but the level of noise varies every trial. Listeners are asked to repeat the sentences and are scored on the number of key words they repeat. If the listener identifies 2 or 3 key words correctly, the level of noise in the next trial increases to make the test harder. This adaptive staircase procedure continues until the Speech Reception Threshold (SRT) is found. The SRT is the minimum signal to noise ratio (SNR) that elicits 50% correct speech recognition, a low SRT is a better score. The Sp-ASL will be completed in three conditions:

a. When noise and speech is coming from the front (co-located SRT)
b. When noise is at the front and speech is on the right (or better ear if preference)
c. When noise is at the front and speech is on the left (or worse ear if preference)

The co-located SRT will be compared across conditions.

Spatial ASL - Spatial Release from Masking (SRM)

Spatial Release from Masking (SRM) is the term used to describe the improvement in masked speech recognition, when the target and masker are spatially separated on the horizontal plane compared to when they are co-located. The SRM will be measured during the Sp-ASL to look at changes in participants performance when separating the speech signal and location of the noise.
SRM is calculated for each noise location as the difference between the co-located SRT and the SRT obtained for each noise location. An average of these two SRM values will be used for analysis.

British Picture Vocabulary Scale (BPVS) test

The BPVS is a vocabulary assessment that can assess a child's receptive vocabulary. The assessment is carried out by the assessor saying a word, which the child responds to by selecting a picture from four options that best illustrates that word's meaning. To ensure the child only encounters items within their critical range, a basal set (where the child gets all or most items right) and a ceiling set (where the child makes a specific number of errors) are established during administration. The raw score is the total number of correct answers provided before reaching the ceiling. The raw score is translated into the age at which an average child would achieve that same score (i.e., an age equivalent score)[12]. We will use the difference between the child's chronological age and the age

equivalent score. In addition, we will use the standardised score, which reflects the relationship between an individual's score with their age-matched peers (scores of 100 are considered average).

Vanderbilt Fatigue Scale (VFS-C) questionnaire

The VFS-C is a unidimensional scale designed to assess listening-related fatigue in children ages 6-17 years. This self-reported measure quantifies listening-related fatigue from the child's own perspective. The scale has ten questions, which are answered on a numerical scale from 0-4. The sum of each item will give a total listening-related fatigue score. Higher scores indicate higher listening-related fatigue [13].

Strengths and Difficulties Questionnaire (SDQ)

The SDQ is a standardized questionnaire designed for children and adolescents aged 2 to 17 years to screen for emotional and behavioural difficulties, as well as strengths in prosocial behaviour[14]It consists of 25 items divided into five subscales: Emotional Symptoms, Conduct Problems, Hyperactivity/Inattention, Peer Relationship Problems, Prosocial Behaviour.

5.2 Analysis Methods

The results of the analyses will be reported following the principle of the ICH E3 and E9 guidelines on the Structure and Content of Clinical Study Reports and Statistical Principles for Clinical Trials[15]. All statistical analysis will be performed using Stata (StatCorp, College Station, TX, USA). Dummy tables are presented in the Appendix.

5.2.1 Adjustment factors

All models will be adjusted for the minimisation factors, unless stated otherwise.

5.2.2 Primary Outcome Analysis

A linear mixed model will be used to compare the speech-in-noise perception scores between the two arms at 3 months. The baseline and three-month measures will be included as response variables. The fixed-effect of the model will include an indicator variable to identify pre-and post-intervention measures, treatment, and the minimisation factors. Random intercept terms will be included to account for clustering within participants, and an additional random intercept may be included to account for clustering by site.

The model will be fitted using restricted maximum likelihood estimation (REML) and will be modelled with an identity covariance structure. The Kenward-Roger correction will be applied to protect against inflation of type I error[16]. Heterogeneity of site cluster sizes may lead to model convergence problems. If the primary analysis model fails to converge the model will be refitted excluding the random site effect.

Results will be presented as an adjusted treatment effect and the associated 95% CI. If the intervention is effective, we would expect to see a positive treatment effect.

The model assumptions will all be checked and if any assumptions are not met, then transformation of speech-in-noise scores may be required.

<u>Sensitivity analysis of primary outcome</u>

We plan to carry out the following sensitivity analyses on the primary outcome using Analysis of Covariance (ANCOVA), including the baseline score as an explanatory variable in the model and fixed effects for the other model components to assess the robustness of results:

1. A small number of participants are expected to have the primary outcome visit outside of the defined window in the protocol (-2 week/ +6 weeks). In such a situation we will carry out an additional analysis by repeating the primary model including participants with data on speech-in-noise measured outside the visit window.

2. If we observe high variation in training exposure based on average game play time per week in >10% of participants in the BEARS arm, an additional sensitivity analysis will be conducted using fixed-effects model with participant specific intercept.[17] This model accounts for participant-specific effects to be arbitrarily correlated with average game play time per week.

5.2.3 Secondary outcome analysis

We will fit similar models to that of the primary outcome to analyse the secondary outcomes, SSiN-VA test outcomes, spatial ASL test outcomes, and VFS at three and 12 months, and BPVS at 12 months.

Quality of life scores (also called “utility scores”) for each arm will be calculated using the newly determined preference-based value set for the YBHRQL-Y (i.e. as determined in work package 5 of the overall programme grant), the existing value set for the HUI-3 and the CHU-9D. They will be compared across the two arms, adjusting for baseline values.

The utility scores will be further used in the health economic analysis, as described in section 6.

5.2.4 Safety reporting

All adverse events (AEs) or serious adverse events (SAEs) occurring during the trial observed by the investigator or reported by the participant will be recorded in the participant’s medical records as per standard practice and on the appropriate BEARS CRFs. The number of participants experiencing at least one SAE, SAR and SUSAR and the number of events

DRAFT

reported during the treatment period will be presented by treatment arm and grade. Full listings of safety events will be provided in statistical reporting.

5.2.5 Subgroup Analysis

Using interaction terms, we will explore whether a differential treatment effect is observed according to the following baseline factors: sex (male vs female), school age group (primary vs secondary), school setting (boarding during the week vs main-stream), severe-to-profound congenital hearing loss (yes vs no), device allocation (iPads or Head Mounted Displays), vocabulary delay (no delay or moderate delay or severe delay), hearing age (used as a continuous outcome defined as the difference between chronological age and age at first implant), socio-economic status (IMD (Index of Multiple Deprivation) decile, education decile, employment decile, IDACI (Income Deprivation Affecting Children Index) decile), parental education (degree or higher degree, A level or HMD/HMC, and all other), parent's occupation,, family's main language spoken at home (English or other), cochlear-implant usage (daily average at baseline) and loudness imbalance between ears (balanced, imbalanced, undetermined).

5.2.6 Exploratory analysis

We will explore whether the effect of BEARS is influenced by levels of training exposure. We will use BEARS training data (total weeks played and game play compliance) to define levels of exposure. The primary outcome model will be fitted with an interaction term between training exposure and time as a continuous variable. In this model, Usual Care will be coded as 0 and training exposure as a continuous variable. This model will assess if the rate of change in outcome over time increases (or decreases) as training exposure increases.

6 HEALTH ECONOMICS ANALYSIS

The aim of the health economic analysis is to calculate the incremental cost per quality-adjusted life-year (QALY) gained when offering BEARS intervention and usual care compared to usual care alone over the twelve months from the NHS and PSS perspective and local education providers.
All analyses will follow intention-to-treat principle.

The primary health economic analysis will be a cost-utility analysis, where QALYs are estimated using YBHRQL-Y outcome measure. Secondary analyses will be cost-utility analyses, where QALYs are estimated using CHU-9D and HUI-3 outcome measures.

Cost of the intervention
The cost of the intervention will be calculated using information on the cost of development of games, licence fees, headset cost, tablet cost and cost of maintenance. We will report a mean cost per participant of delivering the intervention, for the BEARS arm only. Costs

DRAFT

incurred in the form of time spent by health care providers and other professionals in providing support to users of BEARS in using BEARS will also be included here.

Resource use

Resource use will be collected using three resource use measures (RUM) at baseline, 3 and 12 months, which were based on the Client Service Receipt Inventory (CSRI):

- Parents' RUM (primary and community health care service use, urgent or emergency care and hospital admissions and time off school).
- Implant Centres RUM (professionals in the implant centre including rehabilitationist, audiologist, clinical psychologist, surgical team, and others)
- Local education providers RUM (teachers of the deaf, multi-sensory impairment teacher, special educational needs co-ordinator, other communication or teaching assistants, speech and language therapist, other professionals provided by the school). The Local education providers will be sent a copy of the CSRI by a member of the participant's clinical implant centre research team via post or email, which they will complete and return. This will then be uploaded onto OpenClinica by a delegated member of the clinical implant centre research team.

Baseline resource use (covering the 3 months before baseline) will be collected to allow costs to be adjusted for baseline values. At 12 months, Parents' RUM will ask for the preceding 3 months of data (covering the period from 9 to 12 months post baseline). The IC and Local education providers will be asked to provide the preceding 9 months of resource use information at 12-month timepoint. We will report total resource use in each arm as well as according to suitable categories from the RUM.

Cost of resource use

Unit costs will come from routine published sources or mean salaries where applicable. We will report total healthcare resource use costs as well as by categories from the RUM, by arm for each time point.

To calculate the difference in costs between intervention and control arm we will use a linear mixed model. Fixed-effect of the model will include an indicator variable to identify pre- and post-intervention measures, treatment, baseline cost of resource use and the minimisation factors (sex, school age group, school setting, severe-to-profound congenital hearing loss and device allocation). Random intercept terms will be included to account for clustering within participants, and an additional random intercept may be included to account for clustering by site. The confidence intervals will be obtained using bootstrapping procedure with at least 1,000 bootstrap samples.

The analysis will be presented with just NHS+PSS costs, with just local education provider costs, and with NHS+PSS plus local education provider costs. Each of these breakdowns is likely to be of interest to different local and national decision makers that focus on health care provision, or education provision, or both.

Health-related quality of life (HRQL)

Health-related quality of life (HRQL) will be collected using three patient reported outcome measures:

- Self-complete version of the newly validated teenage version of the York Binaural Hearing-Related Quality of Life questionnaire for Young People (YBHRQL-Y)

YBHRQL-Y is a 3-item measure of hearing-related quality of life devised specifically for use with young people (children aged 8 to 16 years old) with hearing loss[18]. It is a preference-based measure. UK preference weights corresponding to participants' responses will be used to calculate the utility scores.

- The proxy completed version of the generic 15-question version of the Health Utilities Index Mark 3 (HUI-3)

The HUI-3 is a preference-based health-related quality of life questionnaire[19]. It uses 15 questions across 8 attributes (vision, hearing, speech, ambulation, dexterity, emotion, cognition and pain). Canadian community preference weights will be used as this is the only validated and standardised tariff.

- The self-complete version of the child specific CHU-9D

CHU-9D is a paediatric health-related quality of life measure that can be used in economic evaluations to calculate the QALY[20]. It contains 9 domains: worried, sad, pain, tired, annoyed, school work/homework, sleep, daily routine, able to join in activities. The measure is validated in the age range 7-17 years. UK preference weights corresponding to participants' responses will be used to calculate utility scores.

Quality-adjusted life years (QALYs)
For each of the three measures, QALYs will be calculated as area under the curve using utility scores obtained by each of the outcome measures described above at the different timepoints. To calculate the mean incremental QALYs between the BEARS intervention and usual care and usual care alone we will use a linear mixed model. Fixed-effect of the model will include an indicator variable to identify pre-and post-intervention measures, treatment, baseline utility score and the minimisation factors (sex, school age group, school setting, severe-to-profound congenital hearing loss and device allocation). Random intercept terms will be included to account for clustering within participants, and an additional random intercept may be included to account for clustering by site. The confidence intervals will be obtained using bootstrapping procedure with at least 1,000 bootstrap samples.

Health economic analysis
We will report the incremental cost-effectiveness ratio (ICER) between BEARS and usual care from an NHS, PSS and local education provider perspective using QALYs calculated using the self-completed YBHRQL-Y. To represent the uncertainty around the cost-effectiveness results, we will report bootstrapped results on a cost-effectiveness plane (CEP) and a cost-effectiveness acceptability curve (CEAC) showing the probability of BEARS being cost-effective compared to usual care at different threshold values.

Missing data
The primary analysis will be a complete case analysis unless >15% of participants are missing an ICER. We will examine the data for predictors of missingness and if predictors of

DRAFT

missingness can be identified these will be used to impute data using the multiple imputation by chained equations. The primary ICER and CEAC will be reported based on imputed results. We will also assess suitability of linear mixed model to account for the missing data.

Additional analyses

Responses collected via the newly validated self-complete version of the YBHRQL-Y questionnaire will be compared against each other and the proxy HUI and self-complete CHU9D to provide further information on how these new measures, previously validated in the preparatory work in Work Package 5 of this programme grant, perform within the larger trial context.

7 REVISION HISTORY

Version	Date	Edited by	Comments/Justification	Timing in relation to first unblinded interim monitoring	Timing in relation to unblinding of Trial Statistician(s)
0.1	13 October 2023	NA	First draft	Prior	Prior
0.2	19 May 2025	NA	Amendments following input from Deborah Vickers and Kashfia Chowdhury	Prior	Prior
0.3	30 October 2025	NA	Details of revised sample size updated in sample size sections	Prior	Prior
0.4	19 February 2026	NA	Amendments following input from Kashfia Chowdhury	Prior	Prior
0.5	21 April 2026	NA	Rationale and outcome scoring methods definitions added. Details of sensitivity analysis methods added.	Prior	Prior
0.6	30 April 2026	NA	Amendments following input from Nicholas Freemantle	Prior	Prior
0.7	15 June 2026	NA	Amendments following input and discussion with Deborah Vickers, Marina Salorio-Corbetto and Caroline Clarke, regarding outcomes and HE outcomes.	Prior	Prior

DRAFT

0.8	28 July 2026	NA	Addition of Health Economics sections edited by Caroline Clarke and Ekaterina Bordea	Prior	Prior

8 APPENDICES

8.1 Dummy Tables

Table A1. Baseline participant characteristics

	**BEARS + Usual Care (N=)**	**Usual Care (N=)**	**Total (N=)**
	Mean (sd)	Mean (sd)	Mean (sd)
**Age/years**			
**Age of deafness in right ear/Years**			
**Age of deafness in left ear/Years**			
**Hearing age**			
**Cochlear implant usage (daily average)**			
**British Picture Vocabulary Scale Standardised Score**			
	n (%)	n (%)	n (%)
**Sex**			
Female			
Male			
**Ethnicity**			
White			
Asian / Asian British			

DRAFT

Black / African / Caribbean / Black British			
Mixed / Multiple ethnic groups			
Other ethnic group			
**School Age Group**			
Primary			
Secondary			
**School Setting**			
Mainstream			
Boarding during the week			
**Primary language at home**			
English			
Other			
**Severe-to-Profound Congenital Hearing Loss**			
Yes			
No			
Unknown			
**Device Allocation**			
Head mounted display			
iPad			
**Vocabulary Delay**			
No delay			
Moderate delay			
Severe delay			
**Parental Education**			
Degree and higher degree			
A level and HMD/HMC			
Other			

Table A2. Primary Outcome

	BEARS + Usual Care (N=)	Usual Care (N=)	Adjusted Coefficient (95% CI)	p-value
**3 months**	Mean (sd)	Mean (sd)		
Speech-in-noise perception score				

Table A3. Secondary Outcomes at 3 months

	BEARS + Usual Care (N=)	Usual Care (N=)	Adjusted Coefficient (95% CI)	p-value
**3 months**	Mean (sd)	Mean (sd)		
SSiN-VA test outcomes				
Relative localisation score				
Average reaction time for word 2 identification				
Average reaction time for location shift selection				
Average (right-left) spatial index for word identification				
Spatial index for relative localization				
**Spatial ASL test outcomes**				
Co-located SRT				

DRAFT

SRM (average across right and left noise location)				
VFS-C questionnaire outcome				
SDQ outcome				

SSiN - VA (Speech-in-noise - Virtual Acoustics), SRT (Speech Reception Threshold), SRM (Spatial Release from Masking), VFS-C (Vanderbilt Fatigue Scale Child self-report version), SDQ (Strengths and Difficulties Questionnaire).

Table A4. Secondary Outcomes at 12 months

	**BEARS + Usual Care (N=)**	**Usual Care (N=)**	**Adjusted Coefficient (95% CI)**	**p-value**
**12 months**	Mean (sd)	Mean (sd)		
SSiN-VA test outcomes				
Relative localisation score				
Average reaction time for word 2 identification				
Average reaction time for location shift selection				
Average (right-left) spatial index for word identification				
Spatial index for relative localization				
**Spatial ASL test outcomes**				
Co-located SRT				
Average SRM (across right and left noise locations)				
**BPVS test difference between chronological age and age equivalent score.**				
**BPVS test standardized score**				

VFS-C questionnaire outcome				
SDQ outcome				

SSiN - VA (Speech-in-noise - Virtual Acoustics), SRT (Speech Reception Threshold), SRM (Spatial Release from Masking), BPVS (British Picture Vocabulary Scale test), VFS-C (Vanderbilt Fatigue Scale Child self-report version), SDQ (Strengths and Difficulties Questionnaire).

Table A5. Safety Outcome

**At least 1 reported event**	**BEARS + Usual Care (N=)**	**Usual Care (N=)**
SAE; n (%)		
SAR; n (%)		
SUSARS; n (%)		

9 REFERENCES

1. Group BCI. CI activity 2017. https://www.bcig.org.uk/wp-content/uploads/2018/02/Overall-data.pdf.2017.
2. Johnston JC, Durieux-Smith A, Angus D, O'Connor A, Fitzpatrick E. Bilateral paediatric cochlear implants: A critical review. International Journal of Audiology. 2009;48(9):601-17.
3. Lammers MJW, van der Heijden GJMG, Pourier VEC, Grolman W. Bilateral cochlear implantation in children: A systematic review and best-evidence synthesis. The Laryngoscope. 2014;124(7):1694-9.
4. Taitelbaum-Swead R, Fostick L. Audio-visual speech perception in noise: Implanted children and young adults versus normal hearing peers. International Journal of Pediatric Otorhinolaryngology. 2017;92:146-50.
5. Anderson S, Kraus N. Auditory Training: Evidence for Neural Plasticity in Older Adults. Perspect Hear Hear Disord Res Res Diagn. 2013;17:37-57.
6. Carlile S. The plastic ear and perceptual relearning in auditory spatial perception. Frontiers in Neuroscience. 2014;8.
7. Firszt JB, Reeder RM, Dwyer NY, Burton H, Holden LK. Localization training results in individuals with unilateral severe to profound hearing loss. Hearing Research. 2015;319:48-55.
8. Godar SP, Litovsky RY. Experience with bilateral cochlear implants improves sound localization acuity in children. Otol Neurotol. 2010;31(8):1287-92.

9. Hofman PM, Van Riswick JGA, Van Opstal AJ. Relearning sound localization with new ears. Nature Neuroscience. 1998;1(5):417-21.
10. Keating P, Dahmen JC, King AJ. Complementary adaptive processes contribute to the developmental plasticity of spatial hearing. Nature neuroscience. 2015;18(2):185-7.
11. Kahan BC, Hindley J, Edwards M, Cro S, Morris TP. The estimands framework: a primer on the ICH E9(R1) addendum. BMJ. 2024;384:e076316.
12. Dunn LMD, Douglas M. The British picture vocabulary scale. 3rd edition ed. UK: GL Assessment Limited; 2009.
13. Hornsby Benjamin WY, Camarata S, Cho S-J, Davis H, McGarrigle R, Bess Fred H. Development and Evaluation of Pediatric Versions of the Vanderbilt Fatigue Scale for Children With Hearing Loss. Journal of Speech, Language, and Hearing Research. 2022;65(6):2343-63.
14. Goodman R. The Strengths and Difficulties Questionnaire: a research note. J Child Psychol Psychiatry. 1997;38(5):581-6.
15. Baber N. International conference on harmonisation of technical requirements for registration of pharmaceuticals for human use (ICH). Br J Clin Pharmacol. 1994;37(5):401-4.
16. McNeish D, Stapleton LM. Modeling Clustered Data with Very Few Clusters. Multivariate Behavioral Research. 2016;51(4):495-518.
17. Hsiao C. Analysis of Panel Data. 3 ed. Cambridge: Cambridge University Press; 2014.
18. Pedley AJ, Somerset S, Vickers D, Jiang D, Kitterick P. Validation and Preference-Based Scoring of the York Binaural Hearing-Related Quality of Life Questionnaire for Young People. Ear Hear. 2026;47(1):202-9.
19. Feeny D, Furlong W, Torrance GW, Goldsmith CH, Zhu Z, DePauw S, et al. Multiattribute and single-attribute utility functions for the health utilities index mark 3 system. Med Care. 2002;40(2):113-28.
20. Stevens K. Assessing the performance of a new generic measure of health-related quality of life for children and refining it for use in health state valuation. Appl Health Econ Health Policy. 2011;9(3):157-69.

DRAFT
