# Supplementary material for: Multicentre phase III randomised controlled trial to evaluate effectiveness of the Both EARS (BEARS) virtual reality training package to maximise hearing abilities in children and young people with bilateral cochlear implants: the BEARS protocol
Source: BMJ Open. 2026 Oct 27;16(10):e128310. doi: 10.1136/bmjopen-2026-128310 (PMC13723878; doi:10.1136/bmjopen-2026-128310)
Supplement: Supplementary file 6 [file bmjopen-2026-128310.reviewer_comments.pdf]

PEER REVIEW HISTORY

BMJ Open publishes all reviews undertaken for accepted manuscripts. Reviewers are asked to complete a checklist review form and are provided with free text boxes to elaborate on their assessment. These free text comments are reproduced below.

ARTICLE DETAILS

Title (Provisional)

A multi-centre phase III randomised control trial to evaluate effectiveness of the Both EARS (BEARS) virtual reality training package to maximise hearing abilities in children and young people with bilateral cochlear implants: the BEARS protocol

Authors

Vickers, Deborah; Buelt, Lisabeth; Arram, Liz; Picinali, Lorenzo; Salorio Corbetto, Marina; Chowdhury, Kashfia; Clarke, Caroline S; Freemantle, Nick; Jiang, Dan; Parmar, Bhavisha; Early, Frances; Driver, Sandra; Bordea, Ekaterina; Hill, Teresa; Cullington, Helen; Kukiewicz, Fiona; Rocca, Christine; Kitterick, Padraig; Corbett, Fleur; Nightingale, Ruth; Blackstone, James; Ahmed, Norin; Somerset, Sarah; Zalk, Nejra; Mahon, Merle

---

This article was not externally reviewed at BMJ Open. Protocols that have been independently assessed prior to submission to BMJ Open are usually fast-tracked to publication on the grounds that further substantial changes will not be possible. This independent assessment will usually be external, independent review for both a substantial grant award from a non-commercial or government funder and ethics approval.
